# Comparative phylogenomic and structural analysis of canonical secretory PLA2 and novel PLA2-like family in plants

**DOI:** 10.3389/fpls.2023.1118670

**Published:** 2023-02-23

**Authors:** Ankush Ashok Saddhe, Martin Potocký

**Affiliations:** ^1^ Institute of Experimental Botany of the Czech Academy of Sciences, Prague, Czechia; ^2^ Department of Experimental Plant Biology, Faculty of Science, Charles University, Prague, Czechia

**Keywords:** sPLA_2_, plant, phylogeny, modelling, pollen, PLA_2_-like

## Abstract

Plant secretory phospholipase A_2_ (sPLA_2_) is a family of lipolytic enzymes involved in the *sn-2* hydrolysis of phospholipid carboxyester bonds, characterized by the presence of a conserved PA2c domain. PLA_2_ produces free fatty acids and lysophospholipids, which regulate several physiological functions, including lipid metabolism, plant growth and development, signal transduction, and response to various environmental stresses. In the present work, we have performed a comparative analysis of PA2c domain-containing genes across plants, focusing on gene distribution, phylogenetic analysis, tissue-specific expression, and homology modeling. Our data revealed the widespread occurrence of multiple sPLA_2_ in most land plants and documented single sPLA_2_ in multiple algal groups, indicating an ancestral origin of sPLA_2_. We described a novel PA2c-containing gene family present in all plant lineages and lacking secretory peptide, which we termed PLA_2_-like. Phylogenetic analysis revealed two independent clades in canonical sPLA_2_ genes referred to as α and β clades, whereas PLA_2_-like genes clustered independently as a third clade. Further, we have explored clade-specific gene expressions showing that while all three clades were expressed in vegetative and reproductive tissues, only sPLA_2_-β and PLA_2_-like members were expressed in the pollen and pollen tube. To get insight into the conservation of the gene regulatory network of sPLA_2_ and PLA_2_-like genes, we have analyzed the occurrence of various cis-acting promoter elements across the plant kingdom. The comparative 3D structure analysis revealed conserved and unique features within the PA2c domain for the three clades. Overall, this study will help to understand the evolutionary significance of the PA2c family and lay the foundation for future sPLA_2_ and PLA_2_-like characterization in plants.

## 1 Introduction

Phospholipases comprise an evolutionarily diverse group of lipolytic enzymes involved in membrane remodeling and hydrolysis of phospholipids into various bioactive lipid derivatives, including free fatty acids (FFA), phosphatidic acid (PA), diacylglycerol (DAG), and lysophospholipids (LPs) (Wang et al., 2012; Chen et al., 2013; Takáč et al., 2019). These lipid derivatives can regulate various physiological functions, including plant growth and development, cellular signaling, and stress management. Based on the site of phospholipid cleavage, phospholipases are classified into three main groups, phospholipase A (PLA), phospholipase C (PLC), and phospholipase D (PLD), each of them exhibiting further variations in the structure, regulation, and catalytic activity. PLA group, which is involved in the hydrolysis of carboxyester bonds from *sn-1*, *sn-2*, or both positions, can be further categorized according to the structural features, catalytic specificity, and calcium (Ca^2+^) requirement, as PLA_1_ (*sn-1* carboxyester bond hydrolysis), secretory PLA_2_ (*sn-2* carboxyester bond hydrolysis), and patatin-like PLA_2_ (hydrolyze both carboxyester bonds at *sn-1* and *sn-2* positions) (Chen et al., 2013; Takáč et al., 2019). PLA_1_ is calcium-independent, has molecular masses ranging from 45–50 kDa, consists of conserved GXSXG motif, and has a catalytic triad (Serine (S), Aspartic acid (D), and Histidine (H)) (Chen et al., 2013). The Arabidopsis genome encodes fourteen PLA_1_ gene members that regulate diverse functions such as plant growth and development, shoot gravitropism, production of jasmonic acid, senescence, and ultraviolet B (UV-B) defense signaling (Ishiguro et al., 2001; Kato et al., 2002; Lo et al., 2004; Hyun et al., 2008; Seo et al., 2008; Ellinger et al., 2010; Seo et al., 2011. Similarly, patatin-like PLA (pPLA) is a large enzyme with a patatin domain that serves as a prime active site and requires Ca^2+^ for catalytic activity (Chen et al., 2013). Arabidopsis encodes thirteen pPLA members involved in several physiological processes, including lipid metabolism, signal transduction, cell growth, and plant responses to biotic and abiotic stresses  (La Camera et al., 2005; Yang et al., 2007; La Camera et al., 2009; Li et al., 2011; Yang et al., 2012; Li et al., 2013). Patatin-like PLA_2_s share a resemblance with the cytosolic animal iPLA2s (group VI) (Balsinde and Balboa, 2005). The other type of phospholipase A_2_, secretory PLA_2_ (sPLA_2_) is a small class of enzymes with low molecular masses ranging from 13–18 kDa, N-terminal secretory signal peptide, and PA2c domain (phospholipase A2; EC 3.1.1.4; SMART accession SM00085) that comprises a calcium-binding motif (YGKYCGxxxxGC) and a catalytic site DACCxxHDxC motif (Lee et al., 2005). The secreted sPLA_2_ variants are the most abundant PLA_2_s across nature and are classified into several subgroups I–III, V, IX–XIV. In plants, sPLA_2_ is evolutionarily grouped into XI IA (PLA_2_-β) and XI IB (PLA_2_-α) clades (Six and Dennis, 2000). It is involved in diverse physiological functions such as plant growth and development, cell elongation, gravitropism, regulation of auxin, and cellular signaling, and it was also implicated in the responses to biotic and abiotic stress (Takáč et al., 2019; Mariani and Fidelio, 2019).

The first successful plant sPLA_2_ enzyme purification and characterization attempt has been reported in developing elm seeds (*Ulmus glabra*) (Ståhl et al., 1998). Afterward, sPLA_2_ encoding cDNAs were isolated from shoots of Oryza sativa and flowers of carnation (*Dianthus caryophyllus*) (Kim et al., 1999; Ståhl et al., 1999). Later, various sPLA_2_ cDNA, genes, and proteins have been isolated and characterized from several plant species, including *Arabidopsis thaliana*, *Solanum lycopersicum*, *Ricinus communis*, *Nicotiana tabacum*, *Triticum durum*, *Linum usitatissimum*, and *Citrus sinensis* (Dhondt et al., 2000; Fujikawa et al., 2005; Liao and Burns, 2010; Fujikawa et al., 2012; Verlotta et al., 2013; Gupta and Dash, 2017). Arabidopsis genome encodes four sPLA2 isoforms (sPLA2-α, β, γ, and δ), that are categorized into two groups, alpha clade (sPLA2-α) and beta clade (β, γ, and δ). All three members of the beta clade (sPLA2-β, γ, and δ) have been shown to play essential roles in pollen development and pollen tube growth (Kim et al., 2011). Arabidopsis sPLA2-α is required for the trafficking of PIN-FORMED auxin efflux transporters to the plasma membrane and negatively regulates the plant’s defense response by repressing the AtMYB30 transcription activity during pathogen infection (Lee et al., 2010; Froidure et al., 2010). AtsPLA_2_-β produces second messengers to enhance light-induced stomatal opening and contributes to cell elongation and shoot gravitropism through the auxin signaling pathway (Lee et al., 2003; Seo et al., 2008).

Despite considerable work on the plant PLA_2_, there is little information on comparative structural studies of plants XI IA (PLA_2_-β) and XI IB (PLA_2_-α) members and no data are available on deep evolutionary comparisons within the plant kingdom (Mansfeld, 2009). Several eukaryotic sPLA_2_ crystal structures have been elucidated, whereas in plants, only the rice sPLA_2_ crystal structure has been solved (Guy et al., 2009). It demonstrated that six disulfide bonds stabilize the rice sPLA*_2_* structure, where the N-terminus contains the conserved Ca^2+^-binding loop, which starts with a short 3_10_-helix and two short antiparallel *β*-strands (Guy et al., 2009). Moreover, the C-terminus is folded into three antiparallel α-helices and contains the conserved catalytic histidine and aspartate (HD) residues (Guy et al., 2009). In the present study, we have analyzed the global distribution of sPLA_2_ members across the plant kingdom. We have identified deep-branching, previously uncharacterized PA2c domain-containing subfamily, termed PLA_2_-like, in plants. We analyzed the evolutionary relationship among sPLA_2_ and PLA_2_-Like members, which separated sPLA2-α, β and PLA_2_-like into three distinct clades. To get more insight into the expression of the three clades, we have compiled tissue-specific expression data for several angiosperm species, which demonstrated the PLA_2_-β members are mainly expressed in the male reproductive tissues. Promoter analysis predicted the presence of tissue, hormone, light, and stress-responsive cis-acting motifs. To uncover conserved structural features in the PA2c fold, we have performed comparative homology modeling of *Amborella trichopoda*, *Arabidopsis thaliana*, and *Nicotiana tabacum* TN90 PLA_2_-α, β, and PLA_2_-like members. Collectively, this study sheds new light on the sequence and structural evolution of the plant sPLA_2_ family.

## 2 Materials and methods

### 2.1 Plant sPLA_2_ homologs identification and annotations

A well-annotated Arabidopsis, rice sPLA_2_-α and β protein sequences were retrieved from Phytozome v13 (https://phytozome.jgi.doe.gov/pz/portal.html) and compiled as initial query sequences. These AtsPLA_2_ query sequences were used to perform BlastP search against thirty-four plant genomes, including Chlorophyta, Bryophyta, Pteridophyta, Gymnosperm, and Angiosperm species in Phytozome v 13 (https://phytozome.jgi.doe.gov/pz/portal.html), ONEKP database (One Thousand Plant Transcriptomes Initiative, 2019) and NCBI database. Moreover, sPLA_2_ sequences of several *Nicotiana* species (*N. tabacum* TN90, *N. sylvestris*, and *N. tomentosiformis*) were searched in the SolGenomics database (Mueller et al., 2005). In subsequent rounds of blast searches, PLA2 sequences from bryophytes, and charophyte and chlorophyte algae were also used as queries. Full-length protein and nucleotide sequences were retrieved and manually checked for the presence of an N-terminal secretory peptide, highly conserved PA2c domain in the ScanProsite (https://prosite.expasy.org/scanprosite/), and Interproscan 5 (https://www.ebi.ac.uk/interpro). Further, partial and truncated sequences that suggested incomplete gene predictions were curated using the SoftBerry FGENESH+ gene prediction algorithm (https://www.softberry.com); short and dubious sequences were removed from the dataset. All sPLA_2_ and PLA_2_-like sequences were compiled in the table with their genomic information, including gene id, protein length, and chromosomal location (**Supplementary Table 1**).

### 2.2 Sequence alignment and phylogenetic analysis

Protein sequences were aligned using the MAFFT E-INS-I algorithm (Katoh and Standley, 2013) in Jalview (Waterhouse et al., 2009). All sequences were manually checked for gaps and non-conserved regions, which were eliminated from the alignment and exported in FASTA file format. The resulting alignment was used to build a sequence logo of the Ca^2+^ binding motif and catalytic motif (https://weblogo.berkeley.edu/logo.cgi) (Crooks et al., 2004). The evolutionary relationship between plant sPLA_2_ and PLA_2_-like was inferred using the IQ-TREE algorithm with Maximum likelihood (ML) supported by the ultrafast bootstrap method (1000 replicates). Model finder was performed and the Whelan and Goldman model with Invariable and gamma (WAG+I+G4) was selected as a best model based on the Bayesian information criterion (BIC) score (Minh et al., 2020). The Interactive Tree Of Life (iTOL v5) (https://itol.embl.de/) online tool was used to display and annotate the sPLA_2_ phylogenetic tree. Likewise, species trees were constructed in NCBI taxonomy and edited in the iTOL server.

### 2.3 Plant sPLA_2_ and PLA_2_-like genes cell and tissue-specific expression analysis

Tissue-specific expression of the sPLA_2_ and PLA_2_-like members have been searched in various vegetative (leaf, stem, root, and seeds) and reproductive (flower, anther, pollen, pollen tube, carpels, pistil, ovary, ovule, and egg cells) tissues of Arabidopsis, Amborella, tomato, grape, rice, and maize using the CoNekT database (https://conekt.sbs.ntu.edu.sg/) (Proost and Mutwil, 2018). Gene expression was represented in transcripts per kilobase million (TPM)-based normalization because it can be used for both gene count comparisons within a sample or between samples of the same sample group (Abrams et al., 2019). The expression values were analyzed in the CIMminer one matrix server (discover.nci.nih.gov/cimminer).

Total RNA was isolated from tobacco leaves, roots, buds, flowers, imbibed pollen, germinating pollen grains and growing pollen tubes using Qiagen RNAeasy Kit, and Turbo DNA-free Kit (Applied Biosystems, Waltham, MA, USA) was used for DNA removal. cDNA synthesis was carried out using Transcriptor High Fidelity cDNA Synthesis Kit (Roche, Penzberg, Germany) with anchored-oligo (DT)_18_ primer according to manufacturer’s instructions. Semi-quantitative RT-PCR was performed with NtPLA_2_ gene-specific oligonucleotides 1-6 (**Supplementary Table 2**) designed to span an intron in the corresponding genomic DNA sequence. Actin7 (Bosch et al., 2005) was used as load control. Amplification conditions were 94°C for 30 sec, 55°C for 30 sec, 68°C for 30 sec and final extension 68°C for 10 min for 28 or 34 cycles.

### 2.4 Cis-acting elements prediction in sPLA_2_ and PLA_2_-like promoters

The promoter regions of sPLA_2_ and PLA_2_-like genes (~ 2kbp upstream of the start codon) were retrieved from the Phytozome v13 database. Promoter sequences were then analyzed *via* the PlantCARE server with default parameters (http://bioinformatics.psb.ugent.be/webtools/plantcare/html). The obtained cis-acting elements data were processed using the CIMminer server.

### 2.5 Prediction of homology models of the sPLA2 and PLA2-like proteins

Homology models of N-terminal truncated peptides signals of *Amborella trichopoda* (ATR0564G112-α; 129 aa, ATR0789G151-β; 118 aa), *Arabidopsis thaliana* (AT2G06925-α; 128 aa, AT2G19690-β; 119 aa), and *Nicotiana tabacum* TN90 (Gene_60450(SS4740)-α; 127 aa, Gene_37244 (SS1768)-β; 117 aa) were obtained through AlphaFold server and also predicted using Robetta server (https://robetta.bakerlab.org/), and RaptorX server (http://raptorx.uchicago.edu/). Moreover, homology models of C-terminal regions (containing PA2c domain) of PLA2-like sequences were predicted for *Amborella trichopoda* (AmTr _scaffold00063; 168 aa), *Arabidopsis thaliana* (AT4G29070- 145 aa), and *Nicotiana tobacum*TN90 (mRNA_158062; 149 aa). For Robetta and RaptorX, five models of each protein were generated, and the model qualities were compared with the AlphaFold predictions using the SWISS-refinement server (https://swissmodel.expasy.org/assess). MolProbity Score, clash score, Ramachandran scores, and QMEAN were considered to select the best model among all generated predictions. Further, electrostatic potentials for the best models were calculated using APBS web-server (Adaptive Poisson-Boltzmann Solver, https://www.poissonboltzmann.org/) and plotted on the predicted protein structures (Unni et al., 2011). The conserved residues and non-conserved residues were analyzed using the Consurf web-server (https://consurf.tau.ac.il/) for the best models. All the generated models were visualized and edited using the CHIMERA (https://www.cgl.ucsf.edu/chimera/).

## 3 Results

### 3.1 Phylogenomic analysis of plant PA2c domain-containing genes revealed an ancient origin of sPLA_2_ and uncovered a widespread presence of the previously uncharacterized PLA_2_-like family

**Figure 1:**
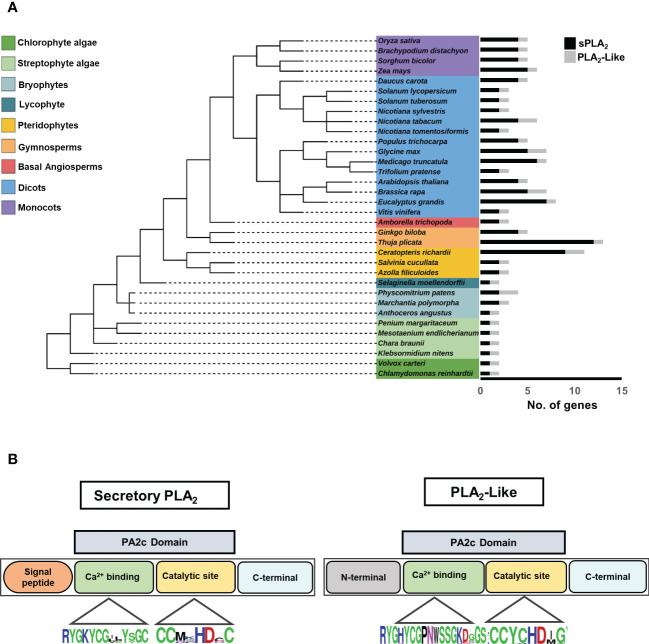
**(A)** Distribution of secretory PLA_2_ and PLA_2_-like genes in the different plant taxonomic groups, including Algae, Bryophytes, Pteridophytes, Gymnosperms, and Angiosperms. The species tree was elucidated using the NCBI taxonomy database and edited in the Interactive Tree of Life (iTOL) web tool. Each histogram corresponds to the number of sPLA_2_ and PLA_2_-like in each species. Black, histogram-sPLA_2_; Gray, histogram-PLA2-like genes. **(B)** Schematic representation of PLA_2_ protein with N and C terminal, conserved PA2c domain, Ca^2+^ binding motif, and HD catalytic dyad.

To get a deeper insight into the evolution of the plant sPLA2 family, we have selected thirty-four plant genomes representing a diverse and balanced sample of *Viridiplantae*, including algae, bryophytes, lycophytes, pteridophytes, gymnosperms, and angiosperms. We searched within this genome sample for the presence of the sPLA_2_ homologs, using known dicot and monocot sPLA_2_ as input and employing various homology-based searches like Blast and HMMER (**Figure 1A**). We ultimately identified 113 non-redundant canonical plant sPLA_2_ genes based on the following criteria: the presence of N-terminal signal peptide and PA2c domain with Ca^2+^ binding motif and catalytic HD dyad site. Surprisingly, we also uncovered 37 additional genes coding for proteins that lack the N-terminal signal peptide but contain a well-conserved PA2c domain, including the calcium-binding motif and the catalytic dyad (**Supplementary Figure 1**, **Supplementary Tables 1**, **3**). The data showed a ubiquitous distribution of both sPLA_2_ and PLA_2_-like genes in plants, from unicellular algae to multicellular flowering plants (**Figure 1A**). Remarkably, the two groups exhibit a clear difference in evolutionary dynamics. PLA_2_-like orthologs were found as single-copy genes in most diploid species and never exceeded two copies per genome. Two PLA2-like genes were found in either polyploid species (*N. tabacum)* or species that underwent relatively recent whole genome multiplications (*B. rapa*, *G. max*, *P. patens*). This suggests that PLA2-like gene number is under the purifying selection to remain singleton. On the other hand, sPLA_2_ genes display much wider genomic plasticity, showing gene numbers ranging from one (chlorophyte and streptophyte algae) to twelve (gymnosperm *T. plicata*). This higher evolutionary dynamics of sPLA_2_ is also apparent for individual plant clades like ferns (two to nine genes) and gymnosperms (four to twelve genes). Within angiosperms, Basal lineages (represented by *A. trichopoda*) seem to contain two sPLA_2_ genes, while distinct dynamics can be found between dicots (two to six genes) and monocots (four to five genes). Despite the general increase in sPLA_2_ gene complexity during plant evolution, reductive events can also be observed. In bryophytes, moss *P. patens* and liverwort *M. polymorpha* have two sPLA_2_ isoforms, whereas only one sPLA_2_ member was observed in hornwort *A. angustus*. Similarly, just one sPLA_2_ was retained in the lycophyte *S. moellendorffii* (**Figure 1A**).

To get more insight into the sequence characteristics of sPLA_2_ versus PLA_2_-like subfamilies, we performed multiple sequence alignments of the two groups and analyzed their overall protein sizes, motif occurrences, and the distribution of conserved residues. All sPLA_2_ sequences have N-terminal signal peptides and range between 90-191 residues, while PLA_2_-like members range between about 143-320 residues, have extended N- and C-terminal regions, and lack any recognizable signal peptide sequence.

The sequence analysis revealed other notable differences among sPLA_2_ and PLA_2_-like members at amino acid levels. Significantly, all sPLA_2_ members possess twelve cysteine residues forming six disulphide bridges and providing extra structural stability  (Mariani and Fidelio, 2019). In contrast, PLA_2_-like members have only six C residues, possibly forming up to three disulphide bridges (**Supplementary Table 3**). Moreover, although the highly conserved Ca^2+^ binding loop is present in all members within the PA2c domain, PLA_2_-like has a subtle variation in Ca^2+^ binding motif with the insertion of an extra residue (YGHYCGxxxxxGK vs YGKYCGxxxxGC) (**Figure 1B**). Another cysteine loss occurred near the catalytic site where sPLA_2_-α and β show invariable DxCCxxHDxC motif, whereas PLA_2_-like has DxCCxxHDxG.

Collectively, our data show a widespread and dynamic occurrence of canonical sPLA_2_ genes and document a highly conserved, evolutionary-constrained subfamily of non-characterized PLA_2_-like genes.

### 3.2 Phylogenetic analysis revealed that sPLA2 and PLA2-like sequences clustered into three deep-branching clades

**Figure 2:**
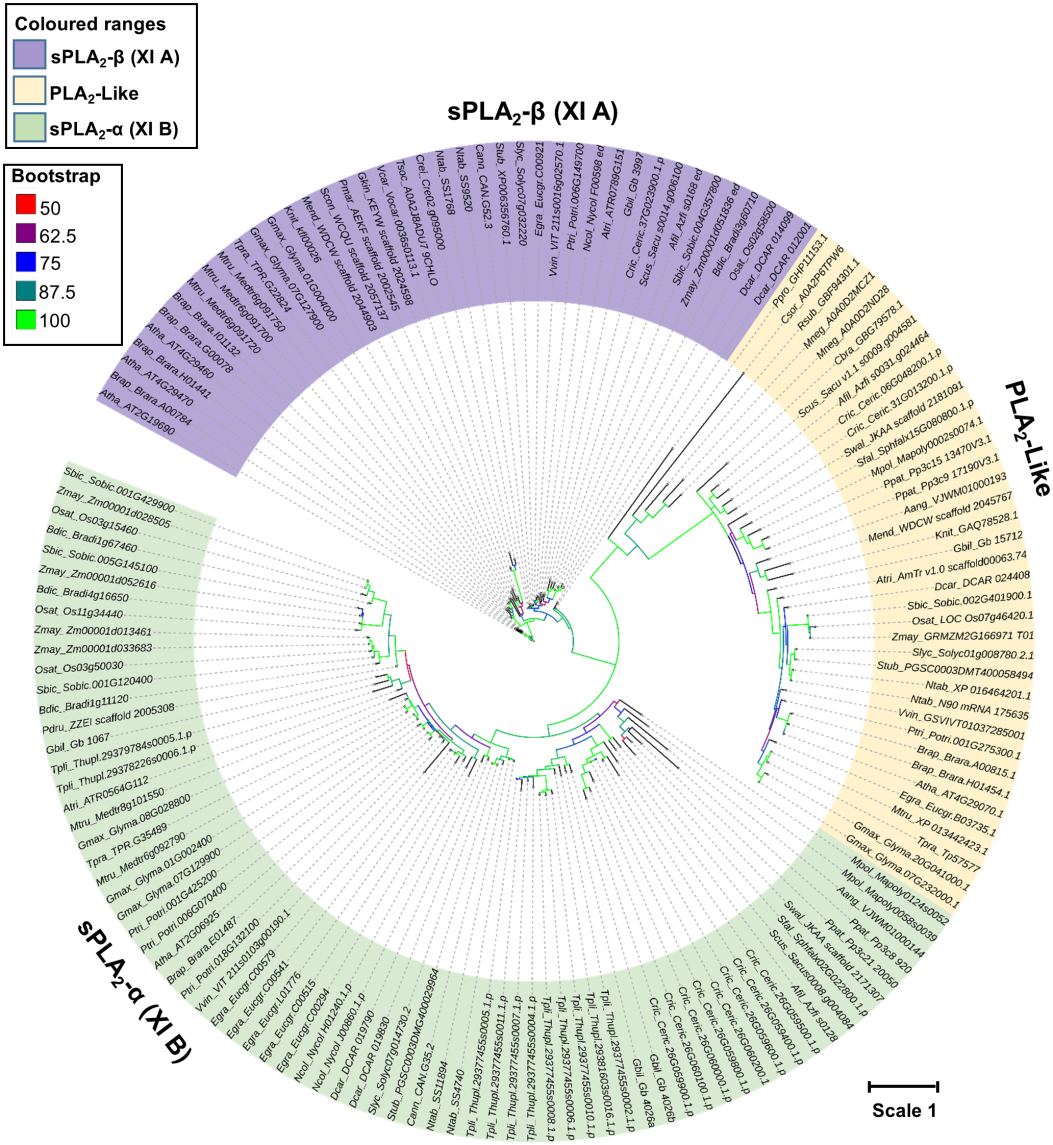
Phylogenetic relationships between sPLA_2_ and PLA_2_ -like members were constructed using the Maximum likelihood (ML) with the ultrafast bootstrap method. The evolution Whelan and Goldman model with Invariable and gamma (WAG+I+G4) was selected based on the Bayesian information criterion (BIC) score. Color nodes correspond to the bootstrap support. The scale bar indicates the rates of substitutions/site. Atha, *Arabidopsis thaliana*; Afil, *Azolla filiculoides*; Aang, *Anthocerus angustus*; Atri, *Amborella trichopoda*; Brap, *Brassica rapa*; Bdic, *Brachypodium distachyon*; Cann, *Capsicum annuum*; Crei, *Chlamydomonas reinhardtii*; Csor, *Chlorella sorokiniana*; Cric, *Ceratopteris_richardii*; Dcar, *Daucus carota*; Egra, *Eucalyptus grandis*; Gbil, *Ginkgo biloba*; Gmax, *Glycine max*; Gkin, *Gonatozygon_kinahanii*; Knit, *Klebsormidium nitens*; Mneg, *Monoraphidium neglectum;* Mpol, *Marchantia polymorpha*; Mtru, *Medicago truncatula*; Mend, *Mesotaenium endlicherianum*; Ntab, *Nicotiana tabacum*; Ncol, *Nymphaea colorata*; Osat, *Oryza sativa*; Pdru, *Phylloglossum drummondii*; Ptri, *Populus trichocarpa*; Pmar, *Penium margaritaceum*; Ppat, *Physcomitrium patens*; Ppro; *Pycnococcus provasolii*; Slyc, *Solanum lycopersicum*; Stub, *Solanum tuberosum*; Sbic, *Sorghum bicolor*; Tpli, *Thuja plicata*; Rsub, *Raphidocelis subcapitata*; Scon, *Staurodesmus_convergens*; Swal, *Selaginella_wallacei*; Sfal, *Sphagnumfallax*; Scus, *Salvinia cucullata*; Tsoc, Tetrabaena socialis; Tpra, *Trifolium pratense*; Vvin, *Vitis vinifera*; Vcar, *Volvox carteri*; Zmay, *Zea mays*.

To shed light on the evolutionary relationships of sPLA_2_ and PLA_2_-like families, a curated dataset of 113 sPLA_2_ and 38 PLA_2_-like genes was compiled from a diverse set of plants with sequenced genome drafts (**Figure 2**). It included evolutionary important chlorophytic and streptophytic algal species such as *Chlamydomonas reinhardtii, Volvox carteri, Penium margaritaceum, Klebsormidium nitens, Mesotaenium endlicherianum*, and *Chara braunii*. Moreover, we have included several bryophytes (moss *Physcomitrium patens*, liverwort *Marchantia polymorpha*, and hornwort *Anthoceros angustus*), lycophyte (*Selaginella moellendorffii*), pteridophytes (*Azolla filiculoides*, *Salvinia cucullata*, and *Ceratopteris richardii*), gymnosperms (*Thuja plicata* and *Ginkgo biloba*), Basal angiosperm (*Amborella trichopoda*), and multiple dicots, and monocots species (**Figure 2**). The sequences were aligned using the MAFFT E-INS-I algorithm, unalignable regions were removed, and a maximum likelihood (ML) tree was constructed using the IQ-tree software with ultra-fast bootstrap support (**Figure 2**).

Despite a limited evolutionary signal (due to relatively short sequence lengths), the resulting tree showed clearly that plant PA2c domain-containing genes separated into three well-supported clades. All sPLA_2_ sequences were clustered into two groups, referred to as sPLA2-α​ clade and β​ clade and corresponding to XI-A (sPLA_2_-β​) and XI-B (sPLA_2_-α​) classification of sPLA_2_ genes (Mansfeld, 2009; Mariani and Fidelio, 2019). Significantly, PLA_2_-like members formed a third independent clade, possibly separating sPLA_2_-α and sPLA_2_-β (**Figure 2**)​​. It should be noted that the two canonical clades seem to possess several specific enzymological characteristics, which were best explored in Arabidopsis and elm. Most importantly, sPLA_2_-α members are show optimal activation by millimolar calcium, while sPLA_2_-β show maximal activity already at micromolar calcium levels. Compared to sPLA_2_ members, newly identified PLA_2_-like members are large in length (>240 aa), high molecular weight, disordered N-terminal region (~100 aa) without signal peptide, and residual variation in Ca^2+^ binding loop. Moreover, the presence of clear sPLA_2_-α and sPLA_2_-β orthologs in gymnosperms and ferns clearly shows that the diversification and stable retention of an α- and β​-clade occurred relatively early in land plant evolution. Interestingly, the contrasting sPLA_2_ distribution in algae and bryophytes (all chlorophyte and streptophyte algae retain 1 sPLA_2_ isoform clustering in the β​-clade, while mosses, liverworts and hornworts possess only α​-clade members), suggests that the two clades may have emerged very early in plant sPLA_2_ evolution, and the single clades were lost in distinct lineages. On the whole, the evolution of the β​-clade is clearly under the evolutionary constraint, both in gene number (except for Brassicaceae) and mutation rate (**Figure 2**). On the other hand, rapidly evolving α​-clade is highly expanded in several lineages, including pteridophytes (*C. richardii*), gymnosperms (T*. plicata and G. biloba*), and angiosperms (*E. grandis, P. trichocarpa, G. max*, and *M. truncatula*).

As mentioned above, PLA_2_-like clade, which consists of primarily single-copy genes, branched-off from sPLA_2_ at the earliest stage of green plant evolution, although its presence in green algae was detected universally (**Supplementary Table 1**). Generally, PLA_2_-like phylogeny roughly follows the species evolution with a mutational rate similar to sPLA_2_-α, suggesting a gradual evolution constrained at the gene number level (**Figure 2**).

### 3.3 Global expression analysis shows the overlapping expression of sPLA_2_​ and PLA_2_-like genes in the sporophyte and suggests a dominant presence of sPLA_2_-β clade members in the male gametophyte

**Figure 3:**
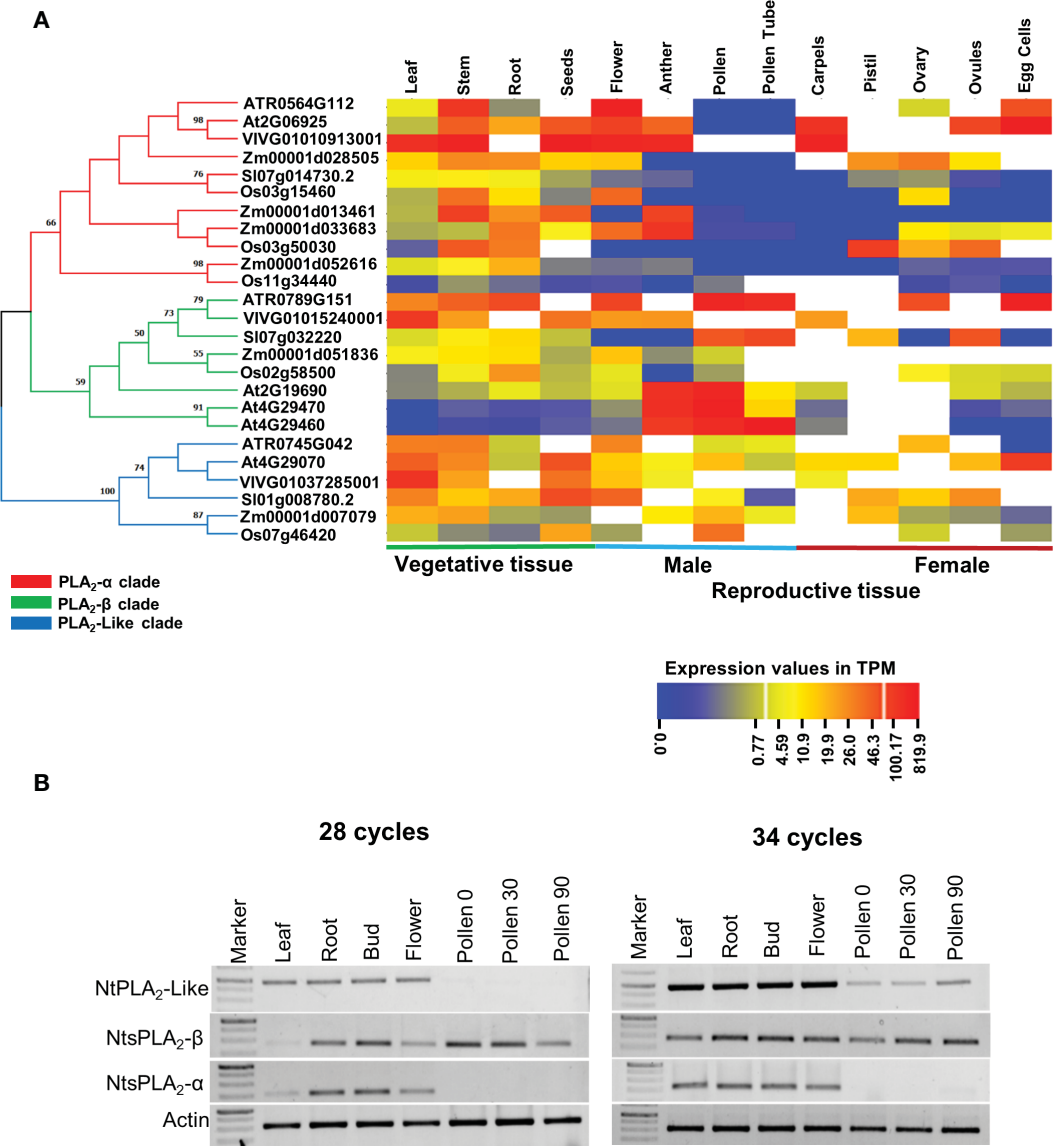
**(A)** Vegetative and reproductive tissue-specific gene expression data of sPLA_2_ and PLA_2_-like members were collected for the selected plant species and arranged on a phylogenetic tree. All collected data were exploited to generate the heatmap using the CIMminer web tool. The expression data was represented as a transcript per million (TPM). The phylogenetic relationships between sPLA2- α, β, and PLA_2_-like members were determined using an ML tree with 100 bootstrap replications in the MEGA X software with the default model. **(B)** Semi-quantitative reverse transcriptase-polymerase chain reaction (RT-PCR) analysis showing the expression of tobacco sPLA2 and PLA2-like members in different tissues and during pollen germination (Pollen30) and pollen tube elongation (Pollen90).

To get a comprehensive evolutionary insight into sPLA2 expression, publicly available tissue-specific RNA-Seq data of representative members of eudicots (*A. thaliana*, *S. lycopersicum*, and *V. vinifera*), monocots (*O. sativa* and *Z. mays*), and Basal angiosperms (*A. trichopoda*) have been extracted and arranged clade-specific on the phylogenetic tree (**Figure 3A**). In total, we analyzed the expression of eleven α​-clade members, eight β​-clade members, and six PLA_2_-like members in four vegetative tissues (leaves, stem, root, and seeds) and ten reproductive tissues (flower, anther, pollen, pollen tube, carpels, pistil, ovary, ovules, and egg cells). The data strongly suggest the existence of conserved clade-specific expression patterns across angiosperms. Members of the sPLA_2_-α clade in all six analyzed species exhibited significant expression in the vegetative tissues, including leaf, stem, root, and seeds. Notably, while most dicot, monocot and Amborella sPLA_2_-α members are expressed in the various maternal reproductive tissues, such as flowers, anther, carpels, ovules, and egg cells, they are all conspicuously absent from pollen and pollen tube.

On the other hand, sPLA_2_-β members across angiosperms showed significant (often the strongest) expression in male gametophytic tissues such as pollen, and pollen tube (and also anther), in addition to variable sporophytic expression. This is well illustrated on the three β-clade and one α-clade Arabidopsis members, where our global expression data analysis is corroborated by earlier RT-PCR and promoter studies  (Bahn et al., 2003; Wang et al., 2008). Thus, AtsPLA_2_-α​ and β​ show similar expression in all tissues except male gametophyte, where AtsPLA_2_-β​ is significantly expressed. Conversely, AtsPLA_2_-γ (and to a lesser extent also AtsPLA_2_-δ) displayed significant expression predominantly in the male gametophyte tissues, including anther, pollen and pollen tube, suggesting that these isoforms might play an essential role in plant reproduction (Bahn et al., 2003; Lee et al., 2005; Wang et al., 2008). The general expression profile of angiosperm PLA_2_-like then suggests a ubiquitous expression with the highest values typically occurring in the sporophyte and species-diversified expression in the male and female gametophytes (**Figure 3A**).

To experimentally corroborate these *in silico* studies, we performed a semiquantitative RT-PCR analysis of tobacco PA2c homologs (please note that the sequences of tobacco sPLA_2_ and PLA_2_-like homoeologous gene pairs are nearly identical (> 98% nucleotide identity) in tobacco amphidiploid genome and were considered as single cDNAs in the RT-PCR analysis). Analysis of leaf, root, bud, flower, dry pollen, germinating pollen and growing pollen tubes showed that tobacco PA2c genes show expression patterns strongly supporting the RNAseq data on other species, particularly the absence of AtsPLA_2_-α in pollen and pollen tubes, where PLA_2_-β is strongly present (**Figure 3B**). Similarly, tobacco PLA_2_-like was expressed predominantly in the sporophyte but could be clearly detectable also in the male gametophyte.

Taken together, our comprehensive examination of available transcriptomic data of six angiosperm species and RT-PCR analysis of sPLA_2_ and PLA_2_-like genes in tobacco showed evolutionarily conserved expression patterns for the three clades, highlighting their overlapping expression in most sporophytic tissues, and suggesting a significant role for the sPLA_2_-β clade in pollen.

### 3.4 Presence of light, stress, and hormone-responsive elements in sPLA_2_ and PLA_2_-like promoters across genomes

**Figure 4:**
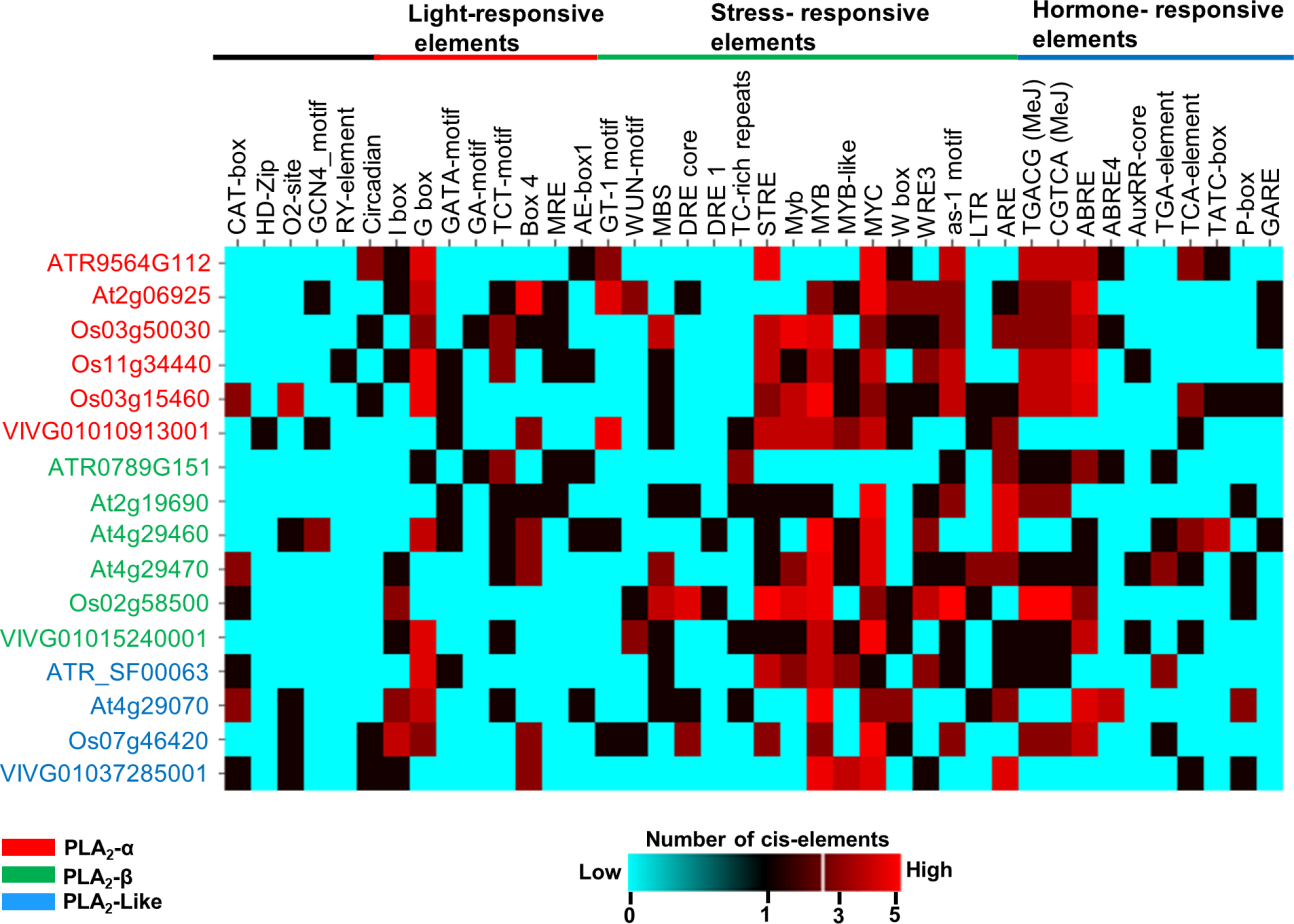
*In-silico* prediction of *cis*-acting elements in the promoter region (5’ upstream region about 2kbp) using PlantCARE database. The number of elements in the promoter regions was depicted by a heatmap using the CIMminer web tool. The color code represents the number of cis-elements in the promoter regions; Cyan- 0 (absent), Red- 5 copies of the element.

Having established conserved expression patterns for the three clades of the angiosperm PLA_2_ family, we next sought to understand their transcription regulatory networks. Therefore, we predicted cis-elements for the promoter sequences of sPLA_2_ and PLA_2_-like genes from *Arabidopsis*, wine, rice, and *Amborella* (**Figure 4**). Various categories of cis-elements were found, including common elements TATA and CAAT box and tissue-specific, light, stress, and hormone-responsive elements.

While no obvious pattern of evolutionarily conserved subset of cis-acting motifs could be attributed to individual sPLA_2_ or PLA_2_-like clades, the analysis strongly suggested that specific stress- and hormone-responsive elements are the most significantly overrepresented category in angiosperm sPLA_2_ and PLA_2_-like genes. These include abiotic stress-responsive elements, like dehydration-responsive element MBS (TAACTG), Myb (CAACTG), MYB (CAACAG), MYB recognition sites (CAACAG), MYB-like sequence (TAACCA) and MYC (CATTTG).

In addition to the abiotic stress-related elements, motifs implicated in biotic interactions, such as wound-responsive elements W-box (TTGACC), WRE3 (CCACCT), and as-1, were detected in most promoter regions for distinct species. Among those, the WRE3 motif is the most abundant and reported in almost all sPLA_2_-α and β gene members except *Amborella* and grape genes. W-boxes were found to interact with transcription factors belonging to the WRKY family, regulate defence-related genes, and play a vital role in biotic and biotic stress, senescence and seed dormancy.

Several hormone-responsive elements were documented in the promoter regions, among which abscisic acid-responsive element ABRE (TACGTG) and methyl jasmonate-responsive cis-elements (CGTCA and TGACG) are the most abundant and conserved, particularly within the sPLA_2_-α clade. On the other hand, ethylene-responsive element ERE (ATTTCAAA), gibberellic acid-responsive elements (GARE: AAACAGA, PA box, and TATC box), auxin responsiveness core element (auxRR: GGTCCAT, TGA elements), show much lower abundance and are scattered among species (**Figure 4**).

Lastly, many light-responsive cis-elements have been documented in the sPLA_2_ and PLA_2_-like promoter regions, including I-box, G box, GATA-motif, GA motif, TCT motif, Box 4, and MRE (MYB binding light responsive elements). The most abundant and conserved motif is G-box (CACGTG), which is involved in the light, abscisic acid, methyl-jasmonate, ethylene and anaerobiosis responses (Sibéril et al., 2001).

Collectively, the abundantly predicted *cis*-acting elements corroborate earlier functions experimentally attributed to selected sPLA_2_ members and suggest a conserved transcriptional control. Indeed, sPLA_2_ transcription was activated in response to blue light (Seo et al., 2008), auxin (Scherer, 2002; Lee et al., 2010), abiotic stresses  (Chapman, 1998), wound stress and pathogen elicitors  (Creelmen and Mullet, 1997; Lee et al., 1997; Laxalt and Munnik, 2002; Ellinger et al., 2010).

### 3.5 Comparative analysis of sPLA_2_ and PLA_2_-like structural models

**Figure 5:**
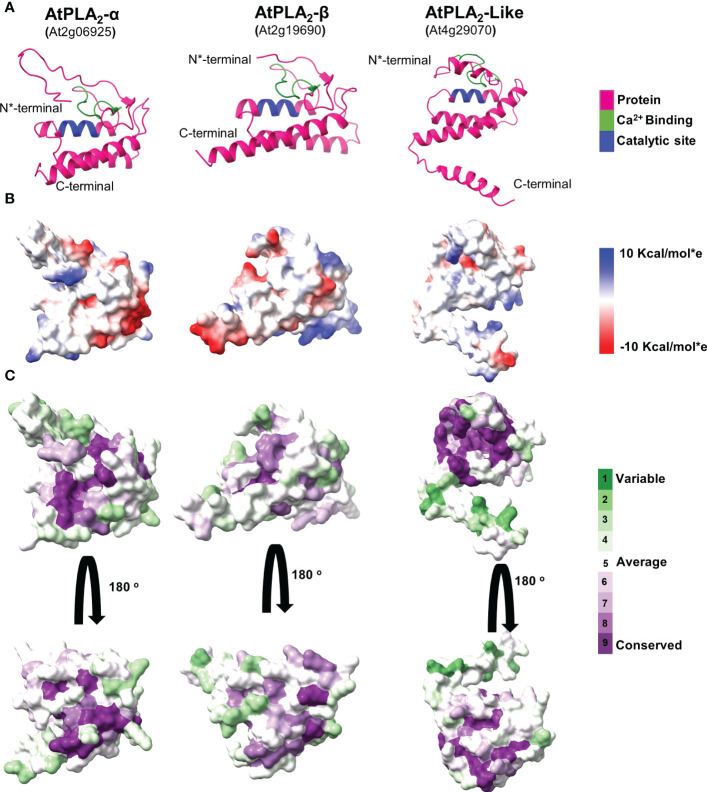
A comparative analysis of predicted *Arabidopsis* PLA_2_ structural models. **(A)** Cartoon representation of sPLA_2_-α, PLA_2_-β, and PLA_2_-like protein models. **(B)** A comparative analysis of the Coulombic electrostatic potential distribution among PLA_2_ members, calculated with the ChimeraX software. **(C)** Analysis of conserved residues on the sPLA_2_ and PLA_2_ surfaces, calculated using the Consurf server.

Despite a wealth of knowledge about PLA_2_ physiological function, there is limited structural data on the plant sPLA_2_-α​, β​, and PLA_2_-like members, thus impeding our understanding of sPLA_2_ substrate preferences, interfacial recognition surface (IRS), and catalytic sites. To get better insight into the structural features of sPLA_2_ and PLA_2_-like proteins, we thoroughly analyzed structural models predicted for representative members of sPLA_2_-α​, β, ​and PLA_2_-like from *Arabidopsis*, tobacco, *Amborella* (**Figure 5**; **Supplementary Figure 2**, **3**). Since the current databases of automatically-generated structural models include the signal peptide, we generated *de novo* models of the processed sPLA_2_ forms. Moreover, since the N-terminal extensions in PLA_2_-like sequences show the characteristics of an intrinsically disordered region, we analyzed only the C-terminal portions. In a pilot analysis, we generated the models using three top-ranked algorithms that are not constrained by existing experimental structures (AlphaFold2_mmseq2 *via* the ColabFold infrastructure, RoseTTAFold, and RaptorX). We assessed them for their folding accuracy based on the Molprobity score, Ramachandran score and Q means plus Z-score criteria (Wang et al., 2016; Waterhouse et al., 2018; Jumper et al., 2021; Baek et al., 2021). Almost universally, AlphaFold-generated models displayed the best criteria and were therefore selected for further analyses (**Supplementary Table 4**).

Comparative analysis of the final validated models revealed that all display a rather tightly-packed globular structure corresponding to the general sPLA_2_ fold (**Figure 5A**; **Supplementary Figure 2**, **3**). Two main structural regions are present across the analyzed species in all sPLA_2_ and PLA_2_-like models. The N-terminal part contains mainly a loop region, including a conserved Ca^2+^-binding loop, and is preceded by two antiparallel beta strands. The C-terminal segment is then represented by three antiparallel α-helices, of which the two first are also present in other secreted PLA_2_s and contain the conserved catalytic histidine and calcium-coordinating aspartate residues (Guy et al., 2009). Similar structural features were documented in PLA_2_-like sequences, including the N-terminal loop region with Ca^2+^ binding motif and three antiparallel helices at the C-terminal region with the extended region. Notably, in canonical sPLA_2_s, the calcium-binding loop and the N-terminal and C-terminal parts are held together by six disulfide cysteine bridges that stabilize the overall structure. However, not all cysteines are conserved in PLA_2_-like sequences, and the structural models suggest that only two cysteine bridges may be retained in PLA_2_-like structures, giving them higher conformational flexibility.

Since sPLA_2_ is an interfacial enzyme interacting with the phospholipid bilayer, we next sought to see the electrostatic potential distribution in all analyzed models that could indicate distinct membrane-binding patterns for separate clades. Our data show that sPLA_2_-β members in all analyzed species contain positively-charged pockets, which may be involved in anionic lipid binding. On the other hand, all PLA_2_-like models lack this feature and produce mostly neutral surfaces. The highest diversity was found within sPLA_2_-α​ clade, where significant charge differences can be found between *Arabidopsis*, *Nicotiana* and *Amborella* members (**Figure 5B**; **Supplementary Figure 2**, **3**). The low evolutionary conservation of the charge distribution was also corroborated by the visualization of evolutionarily conserved surface residues, suggesting that among sPLA_2_-α​, β​, and PLA_2_-like members, there is rather low conservation beyond the Ca^2+^ binding motif and the catalytic region (**Figure 5C**).

## 4 Discussion

### 4.1 Ubiquitous distribution and ancient divergence record of sPLA2 in plants

Despite the critical importance of PLA_2_ documented in higher plants and the availability of detailed molecular information for phospholipases from non-plant organisms, evolution-based knowledge of phospholipase A2 from plants is still meagre. To fill this gap, we have performed a comparative phylogenomic, sequence and structural analysis of the plant’s secretory PLA_2_ family, along with previously uncharacterized PLA_2_-like members. This is the first systematic analysis of the plant family of PA2c domain-containing proteins, covering chlorophyte and streptophyte algal species, bryophytes, lycophyte, pteridophytes, gymnosperms, Basal angiosperm, dicots and monocots. However, the detailed evolutionary history and clade separation of the plant PA2c domain superfamily is murky. Our data demonstrated that one genuine sPLA_2_ can already be documented in multiple algae species (chlorophytes, charophytes and zygnematophytes), and all canonical algal sPLA_2_ fall into the XI-A (sPLA2-β) clade. On the other hand, all canonical sPLA_2_ genes from three main bryophyte groups (hornworts, liverworts, and mosses) belong to the XIB (sPLA_2_-α) clade, and the simultaneous presence of both clades can be first documented in ferns. Three evolutionary scenarios can explain this diverse distribution: (*i*) sPLA_2_-β is the ancestral plant form of secretory phospholipase A_2_, which through gene duplication and subsequent diversification, gave rise to the sPLA_2_-α clade during the plant colonization of earth but was secondarily lost in bryophytes and lycophytes; (*ii*) both clades were present already in the common ancestor of chlorophyte and streptophyte algae and were sometimes lost in particular groups; (*iii*) individual sPLA_2_ clades were subject to the independent horizontal gene transfer events during the evolution of separate Viridiplantae groups. These scenarios are not necessarily mutually exclusive, especially considering the widespread occurrence of horizontal gene transfer in green algae (Ma et al., 2022).

One notable element of plant sPLA_2_ evolution is the diversity in gene copy numbers for the two clades. The terminal duplication and diversification events of sPLA_2_ may be attributed to whole-genome duplication (eg. in some bryophyte species), polyploidization or amphidiploidization events (eg. *N. tabacum *or* Z. mays*)*. *In addition,* *in some pteridophyte and gymnosperm species, sPLA_2_ underwent massive multiplication. Several hypotheses explain plant gene family expansion besides the whole-genome duplications and hybridization events. Gene families can expand through either segmental, tandem, or retro-transposition (RT) mechanism, but segmental and tandem duplication events were more predominant than RT (Kondrashov, 2012; Panchy et al., 2016). These new paralogs may perform an existing gene function (sub-functionalization) or acquire a novel role (neo-functionalization)  (Panchy et al., 2016). These gene duplication mechanisms may often co-occur, as evidenced in the *Eucalyptus* *grandis, *which* *genome (n=11) has been shaped by lineage-specific genome duplication events and a high rate of tandem gene duplication  (Myburg et al., 2014), leading to the highest number of sPLA_2_ members found in the analyzed angiosperms. As noted above, most of these within-family multiplication events occurred in the sPLA_2_-α clade, which is evolving considerably faster than the sPLA_2_-β clade. The notable exception is Arabidopsis (and the whole *Brassicaceae* clade, **Supplementary Table 5**, **Supplementary Figure 5**), where the expansion occurred predominantly in the sPLA_2_-β clade.

### 4.2 Is there an evolutionarily conserved role for the sPLA2-β clade in the male gametophyte endomembranes?

Our analysis of the sPLA2 expression revealed that while both -α​ and -β clades are present in various sporophytic tissues, sPLA2-α​ genes seem to be absent in the male gametophyte in dicots, monocots and Basal angiosperms (**Figure 3A**). This contrasts with the expression of the angiosperm sPLA_2_-β clade members, expressed either exclusively in the pollen and pollen tubes (*Arabidopsis* sPLA2-γ and -δ), or showing the highest expression in the male gametophyte (sPLA_2_-β from *Arabidopsis*, tomato, monocots, and *Amborella*). Significantly, our high-throughput data analysis is corroborated by the RT-PCR analysis of tobacco sPLA_2_ members (**Figure 3B**) and by multiple reports on *Arabidopsis* sPLA_2_ members (Lee et al., 2005; Kim et al., 2011). Although the split between the two sPLA2 clades occurred before the emergence of sexual reproduction *via* pollen, the presence of only sPLA2-β ortholog already in Amborella pollen and pollen tube suggests that the β-clade is the major sPLA2 in all angiosperms. Indeed, Kim et al. (2011) showed that when all three Arabidopsis PLA2-β clade members were suppressed by RNA interference, pollen development and germination were severely affected. Moreover, lysophosphatidylethanolamine, the product of sPLA2 activity, likely plays a vital role in pollen germination and pollen tube growth (Kim et al., 2011). Significant changes in lysophospholipid levels, including plasma membrane lysophosphatidylethanolamine and lysophosphatidylcholine, were recently described during tobacco pollen germination (Serrano et al., 2022). These findings further corroborate the conserved role of sPLA2 in the male gametophyte. Interestingly, compared to other beta-clade members, *Arabidopsis* sPLA2-γ shows the highest expression in the growing pollen tubes, suggesting that the three different β-clade members in Brassicaceae may have distinct roles during pollen development, germination, and tube growth.

A feature reportedly distinguishing plant sPLA2-α​ and -β clades is their distinct localization, suggesting that sPLA2-β may act primarily inside the endomembrane system and are not secreted to the apoplast (Fujikawa et al., 2012);. Three Arabidopsis sPLA2-β clade paralogs (β, γ, and δ) localized mainly to the ER and/or Golgi (Seo et al., 2008; Kim et al., 2011) while sPLA_2_-α localizes either to the apoplast or to the nucleus, depending on the plant status (Froidure et al., 2010; Jung et al., 2012). However, a canonical C-terminal ER-retention signal (KxEL) can be found only in 14 angiosperm sPLA_2_-β members and is missing even in *Arabidopsis* sPLA_2_-γ, and -δ. Therefore, the subcellular localization of sPLA_2_s, particularly the β-clade, might be variable and needs to be determined in other angiosperms.

### 4.3 The widespread presence of novel, evolutionary-constrained, PLA2-like gene family in plants

While annotating the plant’s sPLA_2_ sequences, we came across a subfamily of proteins with unusually long sequences (~250 aa), that possess a conserved PA2c domain containing both Ca^2+^ binding motif and HD catalytic dyad but lacking a signal peptide, which we termed PLA_2_-like. While the presence of the PLA_2_-like subfamily was briefly noticed before (Gupta et al., 2017), its phylogenomic distribution and sequence-structural properties were not explored. Our genome-wide analysis demonstrated that at least one PLA_2_-like member is consistently present from charophyte algae to higher plants, highlighting an ancient origin. It should be noted that PLA_2_-like genes can also be found in chlorophyte algae, but their distribution is patchy, suggesting either frequent losses or horizontal gene transfer in the plant group. Phylogenetically, PLA_2_-like sequences clustered into one clade, representing the independent evolutionary history of PLA2-like members and separating XI-A and XI-B sPLA_2_s. As noted above, the low gene-copy number of PLA_2_-like genes is under strong selection, effectively keeping PLA_2_-like as a single-copy gene in most species. While the putative enzymatic activity of PLA_2_-like remains obscure, the single-copy status is typically linked with genes often involved in essential metabolic processes and/or the formation of macromolecular complexes. Interestingly, the N-termini of PLA_2_-like proteins show intrinsically disordered region features, which function in protein-, DNA-, or RNA- binding (Han et al., 2014).

### 4.4 The angiosperm-conserved involvement of sPLA2 and PLA2-like genes in stress responses and developmental processes

Our analysis of *cis*-acting elements in promoters of evolutionarily-distinct angiosperms species suggested that the transcription of both sPLA_2_ and PLA_2_-like genes is regulated by various biotic, abiotic, and developmental stimuli (**Figure 4**). We have reported different cis-elements (W-box, WRE2, and WUN motif) involved in biotic stress responses, and found several methyl-jasmonate responsive elements in the sPLA_2_ promoters, further suggesting their active involvement during plant reactions to biotic stresses. The transcriptional activation of sPLA_2_ genes after a pathogen attack was shown in diverse species such as Arabidopsis (Froidure et al., 2010) and grapevine (Laureano et al., 2018), supporting our* in-silico* analysis. Analogously, sPLA_2_ promoters have multiple abiotic stress-responsive, abscisic acid-responsive and ethylene-responsive elements in the promoter region, suggesting that sPLA_2_ may have some regulatory roles during abiotic stresses. Verlotta et al., 2013 characterized durum wheat sPLA_2_s and assessed their involvement in drought stress (Verlotta et al., 2013). Similarly, Rice sPLA_2_ also showed a differential expression pattern in response to abiotic stress (Singh et al., 2012).

Besides the involvement in the stress responses, several case studies from distinct species corroborate the conserved transcriptional regulation of sPLA_2_ after developmental cues (**Figure 4**). Arabidopsis sPLA_2_-β is upregulated by auxin and in the curved regions of the peduncle, which were undergoing the gravitropic response (Lee et al., 2003). In *Citrus sinensis*, the sPLA2-α and β genes expression and enzyme activity in leaves and fruits exhibited diurnal rhythmic changes and light regulation, which suggested that diurnal fluctuations in lipophilic second messengers are involved in the regulation of physiological functions (Liao and Burns, 2010).

Very few high-throughput studies described the transcriptional regulation of the* Arabidopsis* PLA_2_-like gene. Interestingly it was demonstrated among genes with expression changes between pollen germination and tube growth  (Wang et al., 2008), and listed among genes involved in *Arabidopsis* acyl lipid metabolism  (Beisson et al., 2003). In the present study, we have predicted that PLA2-like members exhibited ubiquitous expression throughout the vegetative and reproductive tissues. In addition, it has light, stress, and hormonal-responsive elements in the promoter region that may be involved in diverse regulatory mechanisms and cellular functions.

### 4.5 Structural determinants of plant sPLA2 and PLA2-like superfamily

The comparative analysis of structural models for diverse plant sPLA_2_ and PLA_2_-like members strongly suggested that there are only subtle differences in the catalytic sites among typical members of sPLA_2_-α and -β clades. All tested models displayed characteristic sPLA_2_ features, shared with the experimentally-determined structures of non-plant sPLA_2_s from groups I, II, X and also rice XI-B sPLA_2_- namely three α-helices, a short two-stranded β-sheet, and a conserved calcium-binding loop  (Dijkstra et al., 1981; Holland et al., 1990; White et al., 1990; Jabeen et al., 2006; Guy et al., 2009). On the other hand, the total conformation (due to the flexibility of N-terminal and C-terminal regions) and charge distribution differ substantially between the two clades or between different phylogenetic groups. For example, the electrostatic charge differences would affect the membrane-binding and protein-binding properties and thus may impact the sPLA_2_ catalytic functions. Notably, surface charge differences affecting the interactions with negatively-charged phospholipids were also described for non-plant sPLA_2_ members from groups IIA and X (Quach et al., 2014).

Importantly, template-free modelling of the C-terminal half of PLA_2_-like proteins suggested a fold similar to canonical sPLA_2_ structures, including a calcium-binding loop and catalytic dyad. However, the striking difference between the sPLA_2_ and PLA_2_-like is the loss of the disulfide bridges, where only four out of twelve Cys residues - forming two disulfide bridges - are conserved in PLA_2_-like sequences. On the other hand, the two retained disulfide bridges (C140-C167, C166-C192) - which connect helix 1 with the calcium loop and the helices 1 and 2 together - structurally correspond to those that are vital for the PLA_2_ catalytic activity of porcine PLA_2_ (C29-C45, C44-C105, Zhu et al., 1995). Therefore, despite the lower number of disulfide bridges retained in PLA_2_-like structures, PLA_2_-like members may still possess an enzyme catalytic activity towards lipidic substrates analogous to canonical sPLA_2_ (Mansfeld, 2009). In addition to the phylogenomic survey of canonical sPLA_2_ genes, our study may also serve as a call for further enzymological and physiological characterization of the enigmatic PLA_2_-like gene subfamily.

## 5 Conclusions

The widespread distribution of canonical sPLA_2_ and an unexpected presence of novel PLA_2_-like genes throughout the plant kingdom reflects an ancient sPLA_2_ origin and possible early split into two separate clades. This points to the conserved functional importance of plant sPLA_2_. The diverse evolutionary dynamic among the two sPLA_2_ clades and PLA_2_-like clade calls for future functional studies, which will be required to shed light on the functional importance of non-*Arabidopsis* β-clade members in plant reproduction, as well as the molecular characterization of PLA_2_-like genes and their involvement in plant cell physiology.

## Data availability statement

The original contributions presented in the study are included in the article/**Supplementary Material**. Further inquiries can be directed to the corresponding author.

## Author contributions

MP conceived and guided the study. AAS and MP carried out the analyses. AAS and MP wrote the manuscript. All authors contributed to the article and approved the submitted version.

## Supplementary material

Supplementary Figure 1 Sequence alignment of PA2c domain from all analyzed PLA2 proteins - shown separately for sPLA2- α, β **(A)** and PLA2-like **(B)** sequences. Calcium binding motif (red box) and highly conserved catalytic dyad Histidine and aspartic acid (HD) (blue box) are highlighted.

Supplementary Figure 2 Predicted models of Nicotiana tabacum sPLA2-α, β, and PLA2-like. **(A)** Cartoon representation of structures. **(B)** Distribution of electrostatic potential on the PLA2 surface.

Supplementary Figure 3 Predicted models of Amborella trichopoda PLA2-α, β, and PLA2-like. **(A)** Cartoon representation of structures. **(B)** Distribution of electrostatic potential on the PLA2 surface.

Supplementary Figure 4 The positional conservation of cysteine disulfide bridges among canonical sPLA_2_ members from the -α and -β clade and PLA_2_-like proteins. Cysteine residues participating in disulfide bridges are shown in red. Arabidopsis sPLA2 and PLA2-like structural models are shown alongside experimental structure of sPLA2-α from rice.

Supplementary Figure 5 Phylogenetic analysis of selected Brassicaceae family sPLA_2_ members. Phylogenetic tree using *Oryza sativa* sPLA_2_ members as an outgroup was constructed using the Maximum likelihood (PhyML) with the bootstrap method. The tree analysis was performed using the webserver (https://www.Phylogeny.fr: “One Click” Mode) with default parameters. The scale bar indicates the rates of substitutions/site. Abbreviations: AT, *Arabidopsis thaliana*; AL, *Arabidopsis lyrata;* Ah, *Arabidopsis halleri;* Carub*, Capsella rubella;* Cagra*, Capsella grandiflora;* Brara, *Brassica rapa;* Bol, *Brassica oleracea;* Camar, *Cekile maritima;* Sp, *Schrenkiella parvula.*

Supplementary Table 1 List of identified secretory PLA2-α, β, and PLA2-like sequences with their protein length and chromosome locations. (ND – No data, AA – Amino acids).

Supplementary Table 2 Primers used for the RT-PCR analysis.

Supplementary Table 3 Comparison between *Nicotiana tabacum* Phospholipase A_2_ members.

Supplementary Table 4 Assessment of sPLA2 and PLA2 structural models.

Supplementary Table 5 Analysis of sPLA_2_ gene family in several *Brassicaceae* species.

Supplementary Data Sheet 1 Protein sequences of sPLA_2_ and PLA_2_-like analyzed in this study.

Supplementary Data Sheet 2 Promoter sequences of selected sPLA_2_ and PLA_2_-like genes analyzed in this study.
