# Supplementary material for: Comparative phylogenomic and structural analysis of canonical secretory PLA2 and novel PLA2-like family in plants
Source: Front Plant Sci. 2023 Feb 23;14:1118670. doi: 10.3389/fpls.2023.1118670 (PMC9995887; doi:10.3389/fpls.2023.1118670)
Supplement: Supplementary Figure 1 — Sequence alignment of PA2c domain from all analyzed PLA2 proteins - shown separately for sPLA2- α, β (A) and PLA2-like (B) sequences. Calcium binding motif (red box) and highly conserved catalytic dyad Histidine and aspartic acid (HD) (blue box) are highlighted. [file DataSheet_1.docx]

Supplementary Material

# Supplementary Figures and Tables

## Supplementary Figures

**Supplementary Figure 1:** Sequence alignment of PA2c domain from all analyzed PLA2 proteins - shown separately for sPLA2- α, β (**A**) and PLA2-like (**B**) sequences. Calcium binding motif (red box) and highly conserved catalytic dyad Histidine and aspartic acid (HD) (blue box) are highlighted.

**A**


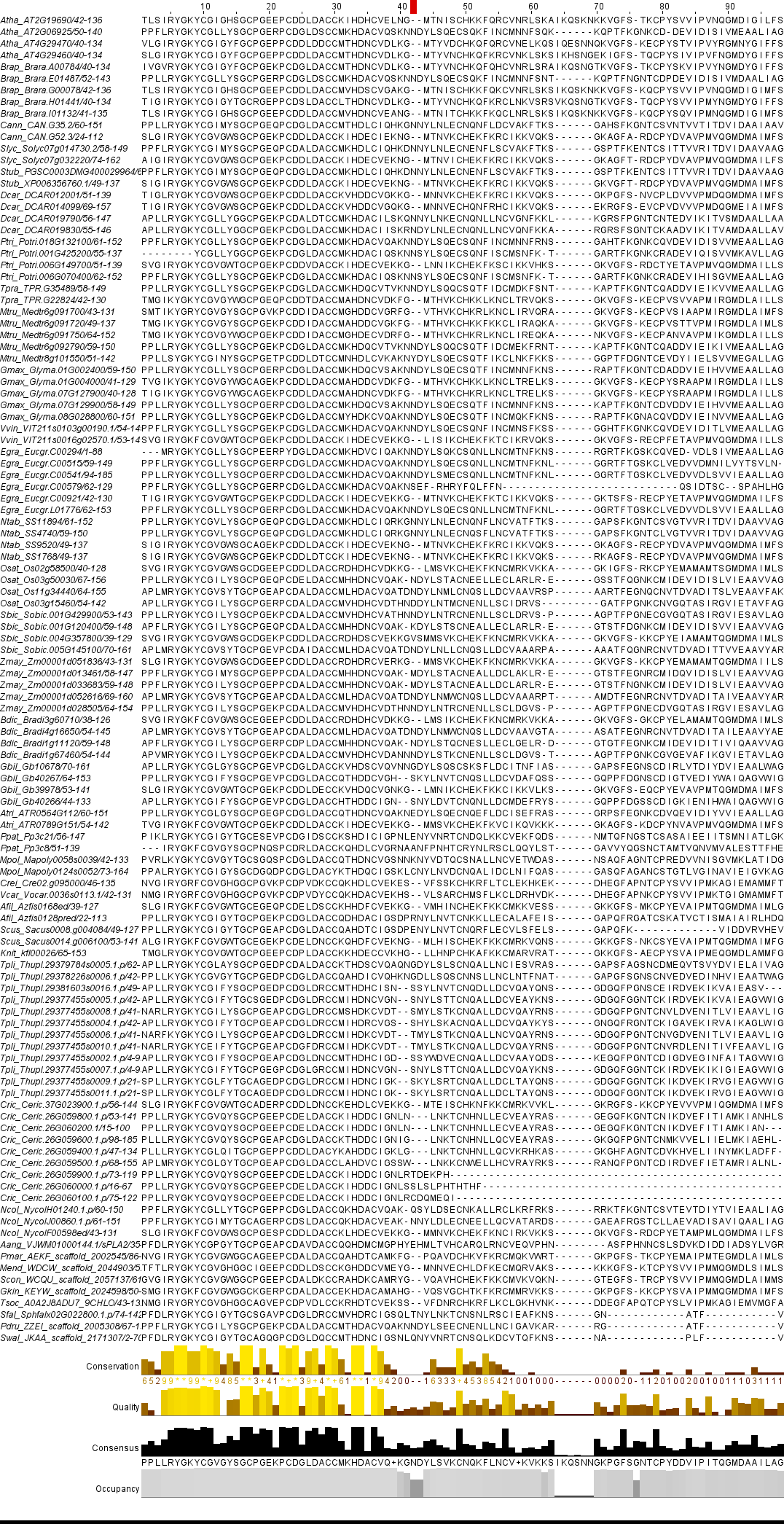


**B**


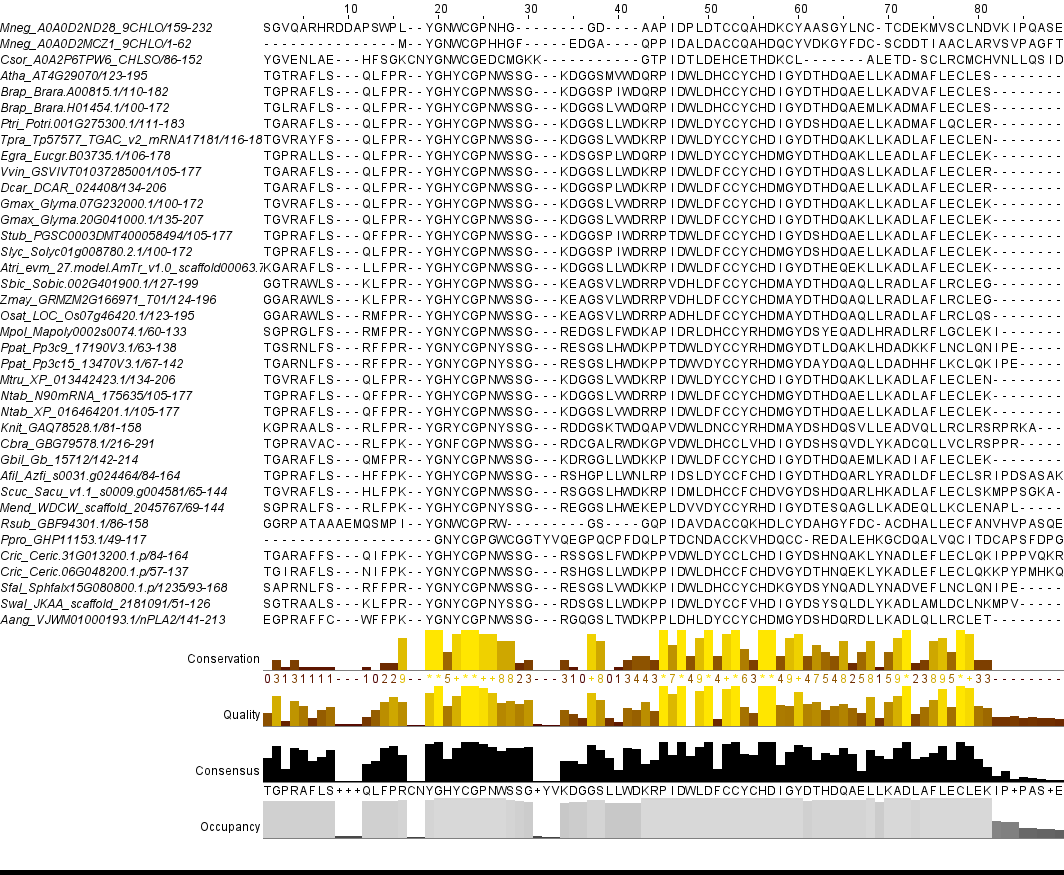


**Supplementary Figure 2:** Predicted models of *Nicotiana tabacum* sPLA_2_-α, β, and PLA_2_-like. (**A**) Cartoon representation of structures. (**B**) Distribution of electrostatic potential on the PLA_2_ surface.


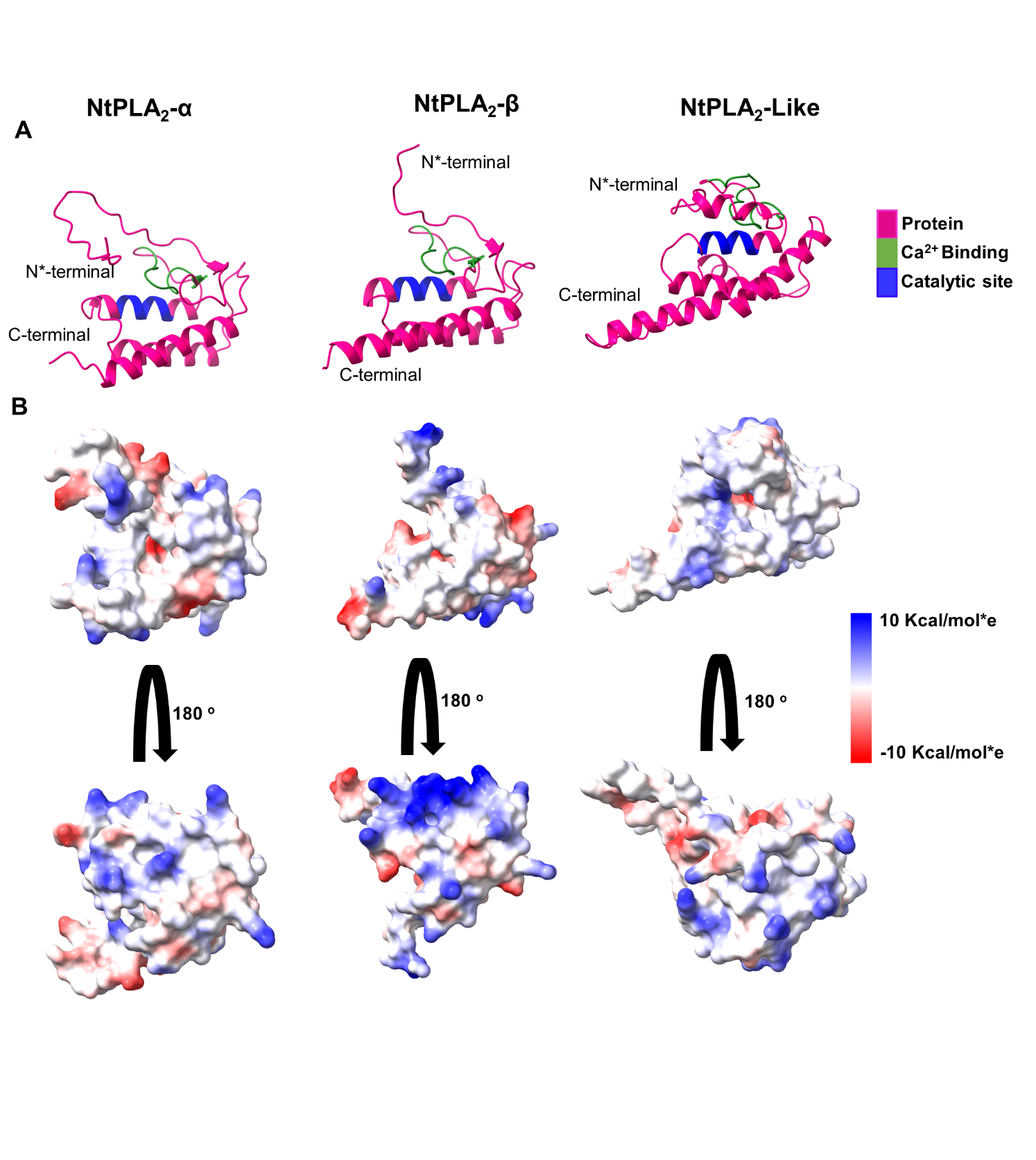


**Supplementary Figure 3:** Predicted models of *Amborella trichopoda* PLA_2_-α, β, and PLA_2_-like. (**A**) Cartoon representation of structures. (**B**) Distribution of electrostatic potential on the PLA_2_ surface.


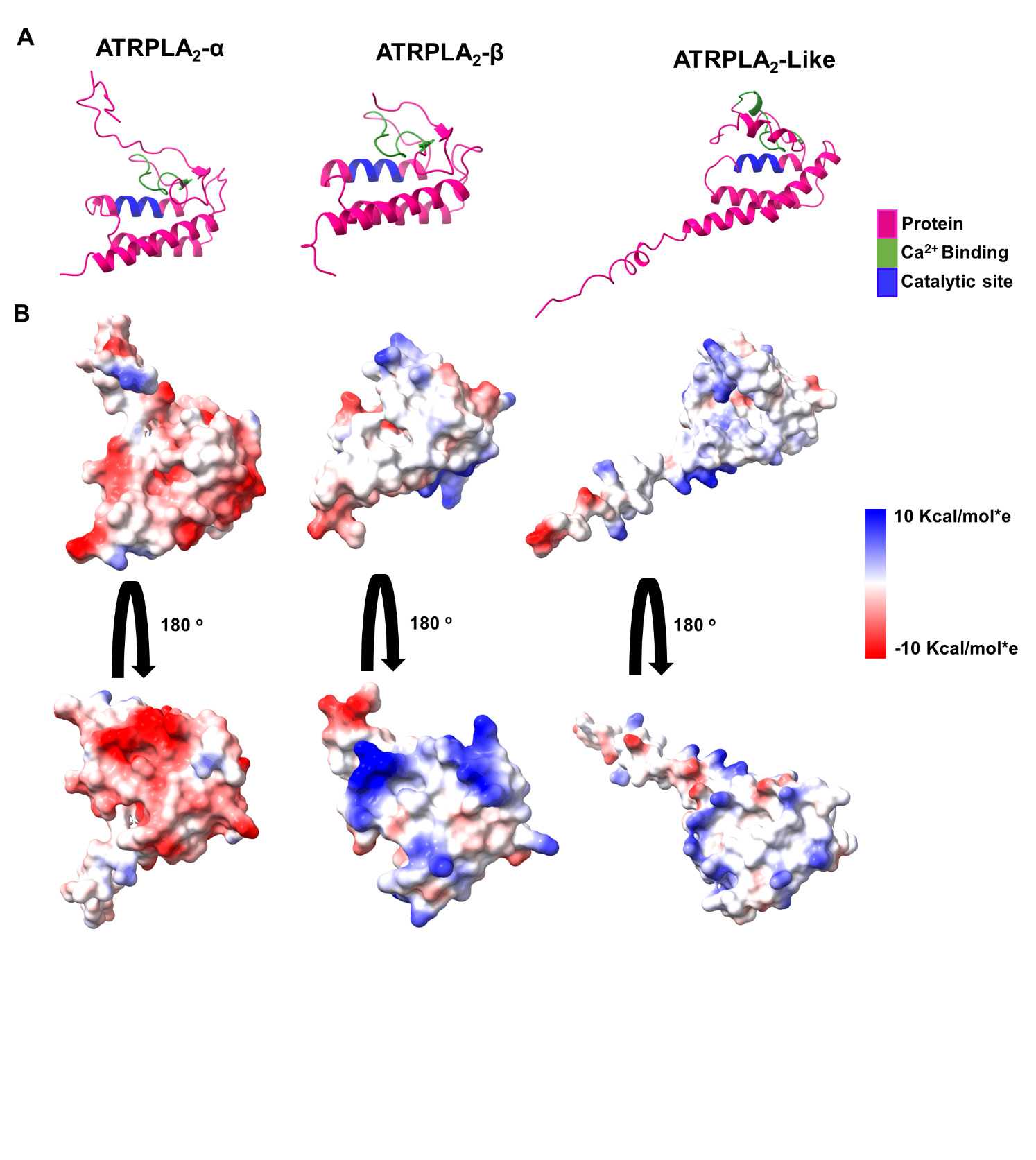


**Supplementary Figure 4:** The positional conservation of cysteine disulfide bridges among canonical sPLA_2_ members from the -α and -β clade and PLA_2_-like proteins. Cysteine residues participating in disulfide bridges are shown in red. Arabidopsis sPLA_2_ and PLA_2_-like structural models are shown alongside experimental structure of sPLA_2_-α from rice.


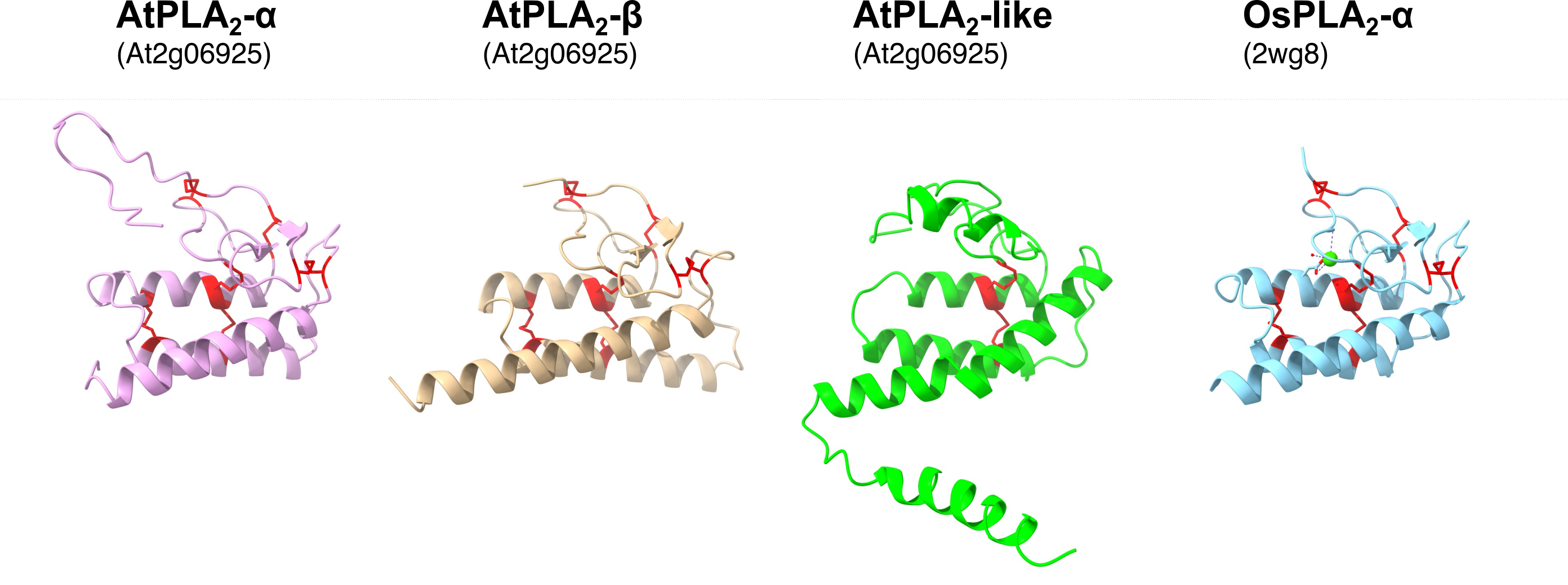


**Supplementary Figure 5:** Phylogenetic analysis of selected Brassicaceae family sPLA_2_ members. Phylogenetic tree using *Oryza sativa* sPLA_2_ members as an outgroup was constructed using the Maximum likelihood (PhyML) with the bootstrap method. The tree analysis was performed using the webserver ([https://www.Phylogeny.fr](https://www.phylogeny.fr/): "One Click" Mode) with default parameters. The scale bar indicates the rates of substitutions/site. Abbreviations: AT, *Arabidopsis thaliana*; AL, *Arabidopsis lyrata;* Ah, *Arabidopsis halleri;* Carub*, Capsella rubella;* Cagra*, Capsella grandiflora;* Brara, *Brassica rapa;* Bol, *Brassica oleracea;* Camar, *Cekile maritima;* Sp, *Schrenkiella parvula.*


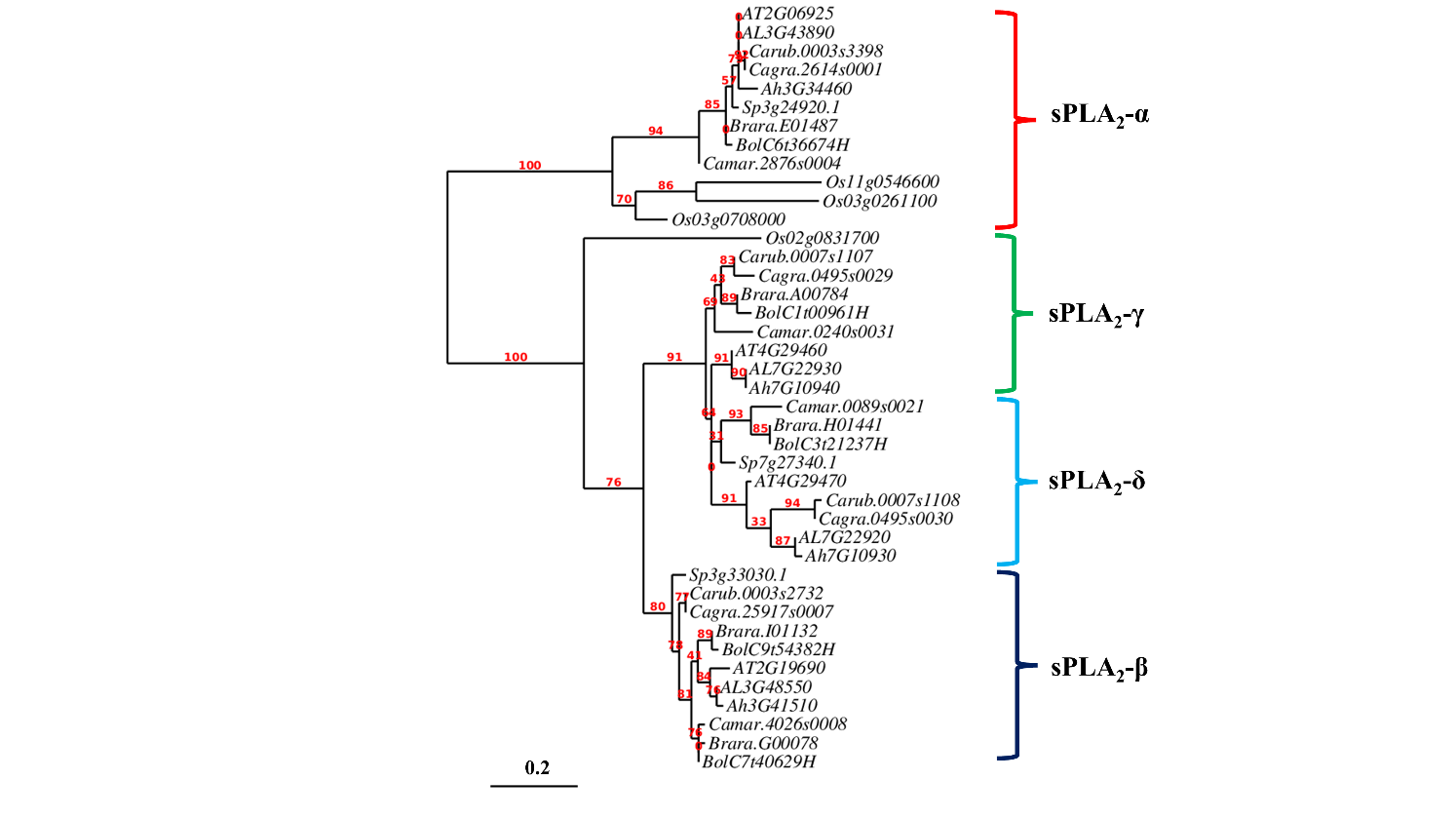


## Supplementary Tables

**Supplementary Table 1:** List of identified secretory PLA_2_-α, β, and PLA_2_-like sequences with their protein length and chromosome locations. (ND – No data, AA – Amino acids).

| **Species Name** | **Gene ID** | **AA** | **Chromosome Location** |
| --- | --- | --- | --- |
| [*Arabidopsis thaliana*](https://bioinformatics.psb.ugent.be/plaza/versions/plaza_v4_dicots/organism/view/Arabidopsis%2Bthaliana) | AT2G19690 | 147 | Chr2 : 8503326-8504549 |
|  | AT4G29470 | 191 | Chr4 :14484591-14485714 |
|  | AT4G29460 | 187 | Chr4 :14483066-14483930 |
|  | AT2G06925 | 148 | Chr2 : 2842475-2843212 |
| *Brassica rapa* | Brara.G00078 | 147 | A07 : 588731-589701 |
|  | Brara.A00784 | 187 | A01 : 3899064-3900003 |
|  | Brara.H01441 | 186 | A08 : 15154659-15155647 |
|  | Brara.E01487 | 150 | A05 : 9663194-9664075 |
|  | Brara.I01132 | 146 | A09 : 6669970-6670761 |
| *Capsicum annuum* | CAN.G35.2 | 158 | 7: 53,840,444-53,850,217 |
|  | CAN.G52.3 | 122 | 7 184,422,302-184,427,046 |
| *Solanum lycopersicum* | Solyc07g014730 | 156 | SL2.50ch07 : 5197657-5200924 |
|  | Solyc07g032220 | 173 | SL4.0ch07:36103775..36117762 |
| *Solanum tuberosum* | PGSC0003DMG400029964 Stub.DM.07G005750 | 162 | ST4.03ch07 : 8357105-8360960 |
|  | XP_006356760 | 148 | ND |
| *Daucus carota* | DCAR_014099 | 167 | Chr4 : 24859017-24861935 |
|  | DCAR_012001 | 149 | Chr3 : 42862056-42864794 |
|  | DCAR_019790 | 154 | Chr5 : 41747365-41748346 |
|  | DCAR_019830 | 153 | Chr6 : 36427574-36428629 |
| *Populus trichocarpa* | Potri.018G132100 | 159 | Chr18 : 15304916-15306428 |
|  | Potri.001G425200 | 144 | Chr01 : 44961679-44962326 |
|  | Potri.006G149700 | 149 | Chr06 : 13007478-13009010 |
|  | Potri.006G070400 | 159 | Chr06 : 5246988-5248046 |
| *Trifolium pratense* | TPR.G35489 | 156 | Tp57577_TGAC_v2_LG5 : 2584769-2586442 |
|  | TPR.G22824 | 140 | Tp57577_TGAC_v2_scaf_967 : 30168-32220 |
| *Medicago truncatula* | Medtr6g092790 | 157 | chr6 : 34944484-34945902 |
|  | Medtr6g091750 | 166 | chr6 : 34553662-34555817 |
|  | Medtr8g101550 | 158 | chr8 : 42680364-42681181 |
|  | Medtr6g091720 | 147 | chr6 : 34546126-34547727 |
|  | Medtr6g091700 | 141 | chr6 : 34541039-34542208 |
| *Glycine max* | Glyma.01G002400 | 157 | Chr01 : 329205-331465 |
|  | Glyma.01G004000 | 138 | Chr01 : 415366-417308 |
|  | Glyma.07G127900 | 138 | Chr07 : 15298152-15299260 |
|  | Glyma.07G129900 | 156 | Chr07 : 15420273-15421798 |
|  | Glyma.08G028800 | 158 | Chr08 : 2307778-2310463 |
| *Vitis vinifera* | VIT_211s0016g02570 | 151 | chr11:2056916..2059450 |
|  | VIT_211s0103g00190 | 152 | chr11:15800725..15801787 |
| *Eucalyptus grandis* | Eucgr.C00294 | 95 | Chr03 : 9955123-9956030 |
|  | Eucgr.C00515 | 174 | Chr03 : 8058611-8060295 |
|  | Eucgr.C00541 | 192 | Chr03 : 8530162-8531611 |
|  | Eucgr.C00579 | 143 | Chr03 : 11255290-11255961 |
|  | Eucgr.C00921 | 140 | Chr03 : 14690940-14692955 |
|  | Eucgr.L01776 | 160 | scaffold_354 : 52790-54133 |
| *N tabacum TN90* | Gene_9932 mRNA_21470 | 160 | Ntab-TN90_AYMY-SS11894:102695..107036 |
|  | Gene_60450 mRNA_129185 | 158 | Ntab-TN90_AYMY-SS4740:566699..570921 |
|  | Gene_85467 mRNA_183309 | 148 | Ntab-TN90_AYMY-SS9520:83126..88740 |
|  | Gene_37244 mRNA_80145 | 148 | Ntab-TN90_AYMY-SS1768:140340..151566 |
| *Oryza sativa* | LOC_Os02g58500  (Os02g0831700) | 138 | Chr2 : 35771130-35772083 |
|  | LOC_Os03g50030  (Os03g0708000) | 163 | Chr3 : 28548725-28549536 |
|  | LOC_Os11g34440  (Os11g0546600) | 164 | Chr11 : 20174792-20176299 |
|  | [LOC_Os03g15460](http://rice.plantbiology.msu.edu/cgi-bin/ORF_infopage.cgi?orf=LOC_Os03g15460.1) (Os3g261100) | 154 | [Chr 3: 8487523-8489366](http://ensembl.gramene.org/Oryza_sativa/Location/View?db=core;g=Os03g0261100;r=3:8487523-8489366;t=Os03t0261100-01;tl=Pha1NkEI4fJ9azIT-168135-114812888) |
| *Sorghum bicolor* | Sobic.001G429900 | 154 | Chr01 : 70912108-70913556 |
|  | Sobic.001G120400 | 155 | Chr01 : 9343880-9344748 |
|  | Sobic.004G357800 | 157 | Chr04 : 68461268-68462439 |
|  | Sobic.005G145100 | 169 | Chr05 : 61031878-61033111 |
| *Zea mays* | Zm00001d051836 | 141 | 4:171675786..171677085 |
|  | Zm00001d013461 | 153 | 5:12111432..12113050 |
|  | Zm00001d033683 | 155 | 1:271130285..271131944 |
|  | Zm00001d052616 | 168 | 4:194836861..194838079 |
|  | Zm00001d028505 | 165 | 1:37343076..37345202 |
| *Brachypodium distachyon* | Bradi3g60710 | 136 | Bd3 : 59335878-59336695 |
|  | Bradi4g16650 | 167 | Bd4 : 17519026-17519875 |
|  | Bradi1g11120 | 155 | Bd1 : 8227498-8228364 |
|  | Bradi1g67460 | 155 | Bd1 : 66350829-66352051 |
| *Amborella trichopoda* | ATR0564G112  (AMTR_s00010p00131910) | 158 | AmTr_v1.0_scaffold00010 : 1820887-1824314 |
|  | ATR0789G151  (AMTR_s00036p00233510) | 152 | AmTr_v1.0_scaffold00036 : 5102492-5106050 |
| *Ginkgo biloba* | Gb_39978 | 151 | ND |
|  | Gb_40266 | 155 | ND |
|  | Gb_40267 | 163 | ND |
|  | Gb_10678 | 168 | ND |
| *Thuja plicata* | Thupl.29379784s0005 | 160 | 29379784:698559..700433 |
|  | Thupl.29378226s0006 | 140 | 29378226:799953..802096 |
|  | Thupl.29381603s0016 | 136 | 29381603:5335663..5336929 |
|  | Thupl.29377455s0005 | 151 | 29377455:461683..462713 |
|  | Thupl.29377455s0008 | 149 | 29377455:484408..485358 |
|  | Thupl.29377455s0004 | 151 | 29377455:457166..458237 |
|  | Thupl.29377455s0006 | 149 | 29377455:469087..470064 |
|  | Thupl.29377455s0010 | 149 | 29377455:504923..505942 |
|  | Thupl.29377455s0002 | 125 | 29377455:234712..235090 |
|  | Thupl.29377455s0007 | 105 | 29377455:481235..481553 |
|  | Thupl.29377455s0009 | 142 | 29377455:486976..487882 |
|  | Thupl.29377455s0011 | 142 | 29377455:520079..520985 |
| *Ceratopteris*  *richardii* | Ceric.26G059600 | 188 | 26:133059667..133075839 |
|  | Ceric.37G023900 | 55 | 37:53879783..53959898 |
|  | Ceric.26G059500 | 159 | 26:132841559..132844953 |
|  | Ceric.26G059400 | 137 | 26:132685772..132696627 |
|  | Ceric.26G059900 | 121 | 26:133789124..133790299 |
|  | Ceric.26G059800 | 144 | 26:133472517..133474765 |
|  | Ceric.26G060000 | 71 | 26:133854686..133855034 |
|  | Ceric.26G060100 | 124 | 26:133856649..133857710 |
|  | Ceric.26G060200 | 103 | 26:133971449..134044752 |
| *Nymphaea colorata* | NycolH01240.1.p | 157 | GWHAAYW00000008:19955032..19957438 |
|  | NycolJ00860.1.p | 157 | GWHAAYW00000010:15243155..15245588 |
|  | NycolF00598 | 141 | GWHAAYW00000006:6364696..6372758 |
| *Azolla filiculoides* | Azfis0168 | 137 | ND |
|  | Azfis0128 | 147 | ND |
| *Salvinia cucullata* | Sacus0008.g004084 | 127 | ND |
|  | Sacus0014.g006100 | 149 | ND |
| *Physcomitrella patens* | Pp3c21_20050 | 178 | Chr21 : 13003245-13004749 |
|  | Pp3c8_920 | 192 | Chr08 : 445024-446263 |
| *Marchantia polymorpha* | Mapoly0058s0039  (Mp5g20610) | 166 | scaffold_58 : 378313-379826 |
|  | Mapoly0124s0052  (Mp5g02710) | 174 | scaffold_124 : 413207-414770 |
| *Volvox carteri f. nagariensis* | Vocar.0036s0113 | 144 | scaffold_36:886682..891174 |
| *Chlamydomonas reinhardtii* | Cre02.g095000 | 142 | Chr2 : 3691035-3692318 |
| *Klebsormidium nitens* | _kfl00026 0180 | 164 | ND |
| *Anthoceros angustus* | VJWM01000144.1 | 145 | ND |
| *Penium_margaritaceum* | AEKF_scaffold_2002545 | 188 | AEKF_scaffold_2002545 |
| *Mesotaenium endlicherianum* | WDCW_scaffold_2044903 | 145 | WDCW_scaffold_2044903 |
| *Staurodesmus_convergens* | WCQU_scaffold_2057137 | 158 | WCQU_scaffold_2057137 |
| *Gonatozygon_kinahanii* | KEYW_scaffold_2024598 | 145 | KEYW_scaffold_2024598 |
| *Tetrabaena socialis* | A0A2J8ADU7_9 | 139 | A0A2J8ADU7_9 |
| *Sphagnum fallax* | Sphfalx02G022800.1.p | 204 | LG02:3462130..3464571 |
| *Phylloglossum drummondii* | ZZEI_scaffold_2005308 | 165 | ZZEI_scaffold_2005308 |
| *Selaginella_wallacei* | JKAA_scaffold_2171307 | 122 | JKAA_scaffold_2171307 |

**List of PLA_2_-like members**

| **Species Name** | **Gene ID** | **AA** | **Chromosome Location** |  |  |
| --- | --- | --- | --- | --- | --- |
| *Arabidopsis thaliana* | AT4G29070 | 259 | 14321807 - 14323336 bp (+) |  |  |
| *Brassica rapa* | Brara.A00815.1 | 246 | A01:4137639..4139140 (-) |  |  |
|  | Brara.H01454.1 | 230 | A08:15236045..15237136 |  |  |
| *Populus trichocarpa* | Potri.001G275300 | 245 | Chr01:28883858..28887881 |  |  |
| *Trifolium pratense* | Tp57577_TGAC_mRNA17181 | 252 | Tp57577_TGAC_scaf_2676:3192..4391 |  |  |
| *Eucalyptus grandis* | Eucgr.B03735 | 240 | Chr02:56939383..56941608 |  |  |
| *Vitis vinifera* | GSVIVT01037285001  (VIT_206s0009g03260) | 240 | chr6:16485011..16485896 |  |  |
| *Daucus carota* | DCAR_024408 | 268 | Chr7:13381266..13382249 |  |  |
| *Glycine max* | Glyma.07G232000 | 247 | Gm07:41605419..41609277 |  |  |
|  | Glyma.20G041000 | 282 | Gm20:7197868..7201534 |  |  |
| *Solanum tuberosum* | PGSC0003DMT400058494  (Soltu.DM.01G005580) | 245 | chr01:5725135..5729796 |  |  |
| *Solanum lycopersicum* | Solyc01g008780.2.1 | 240 | SL4.0ch01:2765246..2770450 |  |  |
| *Nicotiana tabacum* | mRNA_175635 | 245 | AYMY-SS7315:3800..11759 |  |  |
|  | XP_016464201 | 245 | SS8795:58787..66778 |  |  |
| *Medicago truncatula* | XP_013442423.1  (Medtr0180s0050) | 270 | Scaffold0180:15862..17984 |  |  |
| *Amborella trichopoda* | AmTr_scaffold00063.74  **(AMTR_s00063p00194880)** | 268 | Scaffold00063:3500829..3503848 |  |  |
| *Sorghum bicolor* | Sobic.002G401900 | 270 | Chr02:75247481..75248894 |  |  |
| *Zea mays* | Zm00001d007079 | 266 | 2:221781853..221783251 |  |  |
| *Oryza sativa* | LOC_Os07g46420.1 | 266 | Chr7:27699931..27701407 |  |  |
| *Brachypodium distachyon* | Bradi1g19130 | 281 | Bd1:15335489..15336903 |  |  |
| *Gingko biloba* | Gb_15712 | 275 | ND |  |  |
| *Thuja plicata* | Thupl.29382280s0002 | 320 | 29382280:47431..49682 |  |  |
| *Ceratopteris_*  *richardii* | Ceric.31G013200.1.p | 219 | Chr31:24043033..24047386 |  |  |
|  | Ceric.06G048200.1.p | 186 | Chr06:120820655..120823115 |  |  |
| *Azolla filiculoides* | Azfi_s0031.g024464 | 226 | ND |  |  |
| *Salvinia cucullata* | Sacu_v1.1_s0009.g004581 | 193 | ND |  |  |
| *Anthoceros angustus* | VJWM01000193.1 | 220 | ND |  |  |
| *Physcomitrium patens* | Pp3c9_17190V3.1 | 202 | Chr09:11613379..11616983 |  |  |
|  | Pp3c15_13470V3.1 | 224 | Chr15:8946552..8949634 |  |  |
| *Marchantia polymorpha* | Mapoly0002s0074.1 | 196 | scaffold_2:950092..952358 |  |  |
| *Selaginella wallacei* | JKAA_scaffold_2181091 | 179 | scaffold_2181091 |  |  |
| *Volvox carteri* | Vocar.0036s0113 | 143 | scaffold_36:886681..891174 |  |  |
| *Chlamydomonas reinhardtii* | Cre02.g095000 | 142 | 2:3690530..3692451 |  |  |
| *Klebsormidium nitens* | GAQ78528.1 | 224 | ND |  |  |
| *Chara braunii* | Cbra_GBG79578.1 | 391 | ND |  |  |
| *Sphagnum fallax* | Sphfalx15G080800.1.p | 235 | ND |  |  |
| *Pycnococcus provasolii* | Ppro_GHP11153.1 | 313 | PPROV_000988300 |  |  |
| *Raphidocelis subcapitata* | Rsub_GBF94301.1 | 276 | Rsub_06923 |  |  |
| *Mesotaenium endlicherianum* | DCW_scaffold_2045767 | 259 | scaffold_2045767 |  |  |
| *Chlorella sorokiniana* | A0A2P6TPW6_CHLSO | 461 | ND |  |  |
| *Monoraphidium neglectum* | A0A0D2MCZ1_9CHLO | 101 | ND |  |  |
|  | A0A0D2ND28_9CHLO | 274 | ND |  |  |

**Supplementary Table 2**: Primers used for the RT-PCR analysis.

| **Primer label** | **Sequence 5’-3’** |
| --- | --- |
| AS1_NtPLA2 alpha_EXP_F | GCATCATAGCCCTCAGGTT |
| AS2_NtPLA2 alpha_EXP-R | GCAAGCATCAAGTCCATCAC |
| AS3_NtPLA2 beta_EXP_F | GTGTGGCAGAGAACTGTAACT |
| AS4_NtPLA2 beta_EXP_R | CATGTCCATACCCTGTACCATC |
| AS5_NtPLA2-Like_EXP_F | GCCAACAACTGTCAACAAGAAG |
| AS6_NtPLA2-Like_EXP_R | AGCCATCCAAATCCGAAAGATA |
| NtACT7-F | TGCCTATGTTGGTGATGAAGC |
| NtACT7-R | ACCATCACCAGAGTCCAACAC |

**Supplementary Table 3:** Comparison between *Nicotiana tabacum* Phospholipase A_2_ members.

| **Parameters** | **sPLA_2_-α** | **sPLA_2_-β** | **PLA_2_-Like** |
| --- | --- | --- | --- |
| Protein length (AA) | 148 | 158 | 245 |
| Signal Peptide | Present | Present | Absent |
| Mol Wt. (Kd) | ~13-18 | ~16-20 | ~29-30 |
| Domain Features | Ca^2+^ binding, HD catalytic dyad | Ca^2+^ binding, HD catalytic dyad | Ca^2+^ binding, HD catalytic dyad |
| Subcellular Localization | Extracellular region Secreted/Apoplast | Extracellular region Secreted/Apoplast | Nucleus/ Cytoplasm |
| No of cysteine residues and Disulfide bonds | 12 and 6 | 12 and 6 | 6 and 2 |

**Supplementary Table 4** Assessment of sPLA_2_ and PLA_2_ structural models.

|  | **Gene ID** |  | MolProbity Score | Clash Score | Ram- Favoured | Ram- Outliers | QMEAN |
| --- | --- | --- | --- | --- | --- | --- | --- |
| ***Arabidopsis thaliana*** | | | | | | | |
| **RaptorX** | At2g06925_Model_1 | 148 | 3.35 | 216.35 | 89.68% | 2.38% | -0.91 |
|  | model_2 |  | 3.16 | 209.36 | 94.44% | 1.59% | -1.72 |
|  | model_3 |  | 3.32 | 210.64 | 90.84% | 1.59% | -2.65 |
|  | model_4 |  | 3.22 | 201.87 | 92.86% | 1.59% | -0.83 |
|  | model_5 |  | 3.36 | 230.97 | 90.48% | 2.38% | -2.71 |
| **Robetta** | At2g06925_Model_1 | 148 | 3.13 | 197.44 | 94.44% | 1.59% | -0.39 |
|  | model_2 |  | 3.2 | 193.42 | 92.86% | 1.59% | -0.44 |
|  | model_3 |  | 3.16 | 193.42 | 93.65% | 2.38% | -1.06 |
|  | model_4 |  | 3.29 | 185.42 | 89.68% | 1.59% | -2.03 |
|  | model_5 |  | 3.41 | 201.54 | 85.71% | 6.35% | -2.72 |
| **Alfa Fold** | At2g06925_Model1 | 148 | 1.39 | 0.51 | 90.48% | 3.17% | -1.39 |
|  | Truncated |  | 1.72 | 0.44 | 86.99% | 2.74% |  |
| **RaptorX** | At2g019690_Model_1 | 147 | 3.16 | 181.82 | 93.16% | 1.71% | -1.99 |
|  | model_2 |  | 3.31 | 208.12 | 90.60% | 0.85% | -0.74 |
|  | model_3 |  | 3.04 | 190.68 | 95.73% | 1.71% | -1.68 |
|  | model_4 |  | 3.06 | 175.68 | 94.87% | 0 | -0.96 |
|  | model_5 |  | 3.08 | 186.01 | 94.87% | 3.42% | -1.55 |
| **Robetta Server** | At2g019690_Model_1 | 147 | 3.21 | 176.8 | 91.45% | 1.71% | 0.06 |
|  | model_2 |  | 2.93 | 176.03 | 96.58% | 0.85% | 0.26 |
|  | model_3 |  | 2.7 | 174.63 | 98.29% | 0.85% | 0.25 |
|  | model_4 |  | 3.16 | 167.52 | 92.31% | 1.71% | -0.71 |
|  | model_5 |  | 3.21 | 188.67 | 92.31% | 0.85% | -2.17 |
| **AlphaFold** | At2g019690_full length | 147 | 0.88 | 0.44 | 96.55% | 0.69% | -0.92 |
|  | Truncated |  | 0.69 | 0.56 | 99.15% | 0.85% | -0.14 |
| **Robetta** | At4g29070 Model 1 | 259 | 2.69 | 143.33 | 97.67% | 0.78% | -1.26 |
|  | Model 2 |  | 2.97 | 152.01 | 95.33% | 2.72% | -3.1 |
|  | Model 3 |  | 3.01 | 150.31 | 94.55% | 1.17% | -2.13 |
|  | Model 4 |  | 3.17 | 159.82 | 91.44% | 3.50% | -3.42 |
|  | Model 5 |  | 3.11 | 152.63 | 92.61% | 1.95% | -2.9 |
| **RaptorX** | At4g29070 Model 1 | 259 | 3.38 | 204.23 | 87.55% | 3.50% | -3.65 |
|  | Model 2 |  | 3.3 | 196.02 | 89.88% | 1.17% | -2.8 |
|  | Model 3 |  | 3.47 | 205.38 | 87.55% | 5.45% | -4.54 |
|  | Model 4 |  | 3.24 | 167.97 | 89.88% | 2.33% | -2.18 |
|  | Model 5 |  | 3.42 | 197.9 | 84.44% | 3.89% | -5.37 |
| **AlphaFold** | Full length | 259 | 2.45 | 3.86 | 80.54% | 8.95% | -7.75 |
|  | Truncated | 115 | 1.52 | 4.37 | 97.16% | 0.00% | -0.87 |
| ***Nicotiana tabacum*** | | | | | | | |
| **Robetta** | TN90-SS11894_model 1 | 157 | 2.73 | 157.1 | 97.64% | 0.79% | 0.27 |
|  | model 2 |  | 2.91 | 152.2 | 96.06% | 0.00% | -0.5 |
|  | model_3 |  | 2.93 | 156.79 | 96.06% | 0.00% | -0.07 |
|  | model_4 |  | 3.2 | 161.26 | 90.55% | 3.15% | -2.51 |
|  | model_5 | 157 | 3.03 | 156.51 | 94.49% | 0.00% | -1.5 |
| **Raptor X** | SS11894_model 1 |  | 3.33 | 176.94 | 93.70% | 1.57% | -0.97 |
|  | model 2 |  | 3.18 | 200 | 93.70% | 0.79% | -1.52 |
|  | model_3 |  | 3.2 | 196.63 | 92.91% | 1.57% | -1.45 |
|  | model_4 |  | 3.26 | 185.89 | 90.55% | 3.15% | -1.52 |
|  | model_5 |  | 3.27 | 171.47 | 88.98% | 0.79% | -0.65 |
| **AlphaFold** | Full length | 157 | 1.78 | 0.84 | 88.39% | 3.87% | -3.06 |
|  | Truncated | 111 | 0.50 | 0.00 | 98.17% | 0.00% | 0.18 |
| **Robetta** | TN90-SS1768_model 1 |  | 3.41 | 206.46 | 86.09% | 4.35% | -3.08 |
|  | model_2 |  | 3.29 | 194.85 | 90.43% | 0.00% | -0.43 |
|  | model_3 |  | 3.1 | 191.39 | 94.78% | 0.00% | 0.41 |
|  | model_4 |  | 3.38 | 198.97 | 86.96% | 5.22% | -3.05 |
|  | model_5 |  | 3.42 | 203.65 | 85.22% | 4.35% | -2.12 |
| **Raptor X** | SS1768_model 1 | 147 | 2.89 | 206.99 | 97.39% | 0.87% | -0.86 |
|  | model_2 |  | 3.14 | 210.48 | 94.78% | 0 | -0.99 |
|  | model_3 |  | 3.15 | 215.59 | 94.78% | 2.61% | -0.27 |
|  | model_4 |  | 3.17 | 202.05 | 93.91% | 1.74% | -0.09 |
|  | model_5 |  | 3.36 | 218.59 | 89.57% | 1.74% | -1.34 |
| **Alphfold** | Full length | 147 | 1.48 | 0.45 | 90.34% | 3.45% | -2.48 |
|  | truncated | 118 | 1.05 | 0.57 | 94.78% | 1.74% | -0.6 |
| **Robetta** | Ntab_175635 Model 1 |  | 3.03 | 170.76 | 95.06% | 0.00% | -0.87 |
|  | Model_2 |  | 3.04 | 164.83 | 94.65% | 0.82% | -1.11 |
|  | Model 3 |  | 3.02 | 156.93 | 94.65% | 1.65% | -1.87 |
|  | Model 4 |  | 2.97 | 156.29 | 95.47% | 0.41% | -1.29 |
|  | Model 5 |  | 3.16 | 164.34 | 92.18% | 1.23% | -1.31 |
| **RaptorX** | Model 1 |  | 3.36 | 211.47 | 88.89% | 2.88% | -3.74 |
|  | Model_2 |  | 3.36 | 207.38 | 88.80% | 2.90% | -3.5 |
|  | Model 3 |  | 3.39 | 201.27 | 86.42% | 4.94% | -5.04 |
|  | Model 4 |  | 3.43 | 204.49 | 85.19% | 4.53% | -4.42 |
|  | Model 5 |  | 3.51 | 215.91 | 81.07% | 7.00% | -5.73 |
| **AlphaFold** | Full length | 245 | 2.11 | 1.51 | 83.95% | 2.06% | -5.38 |
|  | Truncated | 146 | 1.76 | 2.12 | 91.72% | 0.69% | -1.47 |
| ***Amborella trichopoda*** | | | | | | | |
| **Robetta** | ATR112_model1 |  | 2.9 | 149.06 | 96.06% | 1.57% | 0.39 |
|  | model2 |  | 3.07 | 169.65 | 94.49% | 0.79% | -0.87 |
|  | model3 |  | 2.94 | 162.63 | 96.06% | 0.79% | 0.2 |
|  | model4 |  | 3.08 | 159.21 | 93.70% | 1.57% | -1.36 |
|  | model5 |  | 2.96 | 168.91 | 96.06% | 0.79% | -1.33 |
| **RaptorX** | ATR112_model1 |  | 3.1 | 181.7 | 94.49% | 0.79% | -0.23 |
|  | model2 |  | 3.11 | 169.94 | 93.70% | 0.79% | -0.97 |
|  | model3 |  | 3.19 | 178.57 | 92.13% | 0.79% | -0.72 |
|  | model4 |  | 3.19 | 178.05 | 92.13% | 1.57% | -1.36 |
|  | model5 |  | 3.29 | 178.86 | 88.98% | 0.79% | -1.62 |
| **AlphaFold** | Full length | 158 | 1.83 | 0.43 | 83.97% | 7.05% | -5.07 |
|  | Truncated | 129 | 1.07 | 0.53 | 96.85% | 0.79% | -0.90 |
| **Robetta** | ATR151_model1 |  | 3.19 | 214.72 | 93.97% | 0.86% | 0.32 |
|  | model2 |  | 3.24 | 216.38 | 93.10% | 2.59% | 0.07 |
|  | model3 |  | 3.26 | 210.06 | 92.24% | 3.45% | 1.1 |
|  | model4 |  | 3.43 | 215.71 | 86.21% | 1.72% | -1.77 |
|  | model5 |  | 3.19 | 214.05 | 93.97% | 1.72% | -0.81 |
| **RaptorX** | ATR151_model1 | 152 | 3.14 | 210.21 | 94.83% | 0.00% | 0.4 |
|  | model2 |  | 3.19 | 193.32 | 93.10% | 0.00% | 0.24 |
|  | model3 |  | 2.92 | 220.88 | 97.41% | 0.00% | -0.15 |
|  | model4 |  | 3.17 | 205.47 | 93.97% | 0.86% | -0.73 |
|  | model5 |  | 2.92 | 225.06 | 97.41% | 1.72% | -0.81 |
| **AlphaFold** | Full length | 152 | 1.97 | 0.43 | 81.33% | 7.33% | -4.30 |
|  | Truncated | 111 | 0.95 | 0.60 | 100.00% | 0.00% |  |
| **Robetta** | ATR880 Model1 | 268 | 2.93 | 165.52 | 96.24% | 1.50% | -0.68 |
|  | model2 |  | 3.03 | 168.89 | 95.11% | 1.88% | -1.67 |
|  | model3 |  | 3.04 | 166.86 | 94.74% | 1.13% | -1.07 |
|  | model4 |  | 3.09 | 167.62 | 93.98% | 1.13% | -1.83 |
|  | model5 |  | 3.1 | 164.38 | 93.61% | 2.63% | -1.07 |
| **AlphaFold** | Full length | 268 | 2.18 | 1.81 | 84.96% | 4.51% | -5.56 |
|  | Truncated | 140 | 1.83 | 0.88 | 92.03% | 2.17% | -2.35 |

**Supplementary Table 5:** Analysis of sPLA_2_ gene family in several *Brassicaceae* species.

| **Species** | **sPLA_2_-α** | **sPLA_2_-β** | **sPLA_2_-γ** | **sPLA_2_-δ** | **Total** |
| --- | --- | --- | --- | --- | --- |
| *Arabidopsis thaliana* | AT2G06925 | AT2G19690 | AT4G29460 | AT4G29470 | 4 |
| *Arabidopsis lyrata* | AL3G43890 | AL3G48550 | AL7G22930 | AL7G22920 | 4 |
| *Arabidopsis halleri* | Ah3G34460.1 | Ah3G41510.1 | Ah7G10940.1 | Ah7G10930.1 | 4 |
| *Capsella rubella* | Carub.0003s2732.1 | Carub.0003s3398.1 | Carub.0007s1108.1 | Carub.0007s1107.1 | 4 |
| *Capsella grandiflora* | Cagra.2614s0001.1 | Cagra.25917s0007.1 | Cagra.0495s0030.1 | Cagra.0495s0029.1 | 4 |
| *Brassica rapa* | Brara.E01487.1 | Brara.G00078.1  Brara.I01132.1 | Brara.H01441.1 | Brara.A00784.1 | 5 |
| *Brassica oleracea* | BolC6t36674H | BolC7t40629H  BolC9t54382H | BolC3t21237H | BolC1t00961H | 5 |
| *Cekile maritima* | Camar.2876s0004 | Camar.4026s0008 | Camar.0089s0021 | Camar.0240s0031 | 4 |
| *Schrenkiella parvula* | Sp3g24920.1 | Sp3g33030.1 | Not present | Sp7g27340.1 | 3 |

# Supplementary Data

**Supplementary Data S1:** Protein sequences of sPLA_2_ and PLA_2_-like analyzed in this study.

>Atha_AT2G19690

MMFRTSLMRFAAAFFAIVFVVLVGVARSEECTRTCIAQNCDTLSIRYGKYCGIGHSGCPGEEPCDDLDACCKIHDHCVELNGMTNISCHKKFQRCVNRLSKAIKQSKNKKVGFSTKCPYSVVIPTVNQGMDIGILFSQLGNDMKTEL

>Atha_AT2G06925

MAAPIILFSFLLFFSVSVSALNVGVQLIHPSISLTKECSRKCESEFCSVPPFLRYGKYCGLLYSGCPGERPCDGLDSCCMKHDACVQSKNNDYLSQECSQKFINCMNNFSQKKQPTFKGNKCDD EVIDVISIVMEAALIAGKVLKKP

>Atha_AT4G29470

MIRGGALTHVALGLTVFLLLAVVHSQEKCSKTCIAQKCNVLGIRYGKYCGIGYFGCPGEPPCDDLDDCCMTHDNCVDLKGMTYVDCHKQFQRCVNELKQSIQESNNQKVGFSKECPYSTVIPTVYRGMNYGIFFSGIGNIIIPKKPASAGPVVEVDLARSKADTKDGLGTNQGPQTKDGSKVSVPMNPSPS

>Atha_AT4G29460

MITGLALSRVAFGLTAFLLLAVVSSQEKCSNTCIAQNCNSLGIRYGKYCGIGYFGCPGEPPCDDLDACCMTHDNCVDLKGMTYVNCHKQFKRCVNKLSKSIKHSNGEKIGFSTQCPYSIVIPTVFNGMDYGIFFSGIGNIFNPPVLGSVPVVEVDLARSKVDTKDGLGTKLGLQTKEGSKVSASLNI

>Brap_Brara.A00784

MVYGGALSRFSFGLATFLLFTFVLSKEKCSKTCIAKNCNIVGVRYGKYCGIGYFGCPGEKPCDGLDACCMTHDNCVDLKGMTYVNCHKQFQHCVNRLSRAIKQSNGTKVGFSTKCPYSKVIPTVYNGMDYGIFFSKIGNIFKPRVPGKAPRVEVNLARSKADTKDGLGTKVALQRKQGSKKVSATLS

>Brap_Brara.E01487

MAAPIILSCFLFLFFFSVSVSALNVGVQLTHPTVSLSKECSRKCESEFCSVPPLLRYGKYCGLLYSGCPGERPCDGLDSCCMKHDACVQSKNNDYLSQECSQKFINCMNNFSNTKQPTFNGNTCDPDEVIDVISIVMDAALIAGRVFRKP

>Brap_Brara.G00078

MMMVGSALTRFVGAFFLVIFLLADVVRCEECTRTCIAQNCDTLSIRYGKYCGIGHSGCPGEEPCDDLDACCMVHDSCVGAKGMTNISCHKKFQKCVNRLSKSIKQSKNKKVGFSKQCPYSVVIPTVNQGMDIGIMFSQLGNDMRTEL

>Brap_Brara.H01441

MMNGGALTRFTFSVATFLLLTVVRSQEPCSKTCIAQDCATIGIRYGKYCGIGYTGCRGEPPCDSLDACCLTHDNCVDLKGMTYVNCHKQFKRCLNKVSRSVKQSNGTKVGFSTQCPYSVVIPTMYNGMDYGIFFSGIGNILEPPAPGKGPVVEVNLAQSGADTKGGLGTKVDIQKKEGSKVSASLN

>Brap_Brara.I01132

MMISSSSMRVAAALLLVLLFLVDVVCSEECTRTCIAQNCDTLSIRYGKYCGIGHSGCPGEEPCDDLDACCMVHDNCVEANGMTNITCHKKFKQCLNRLSKSIKQSKNKKVGFSKQCPYSQVIPTMNQGMDIGIMFSQLGNDLRTEL

>Cann_CAN.G35.2

MASLQSLKLSLQLLAFSIIAFCINSPISIHALNIGIETNAGLSLEKECSRTCESKFCIVPPLLRYGKYCGIMYSGCPGEQPCDGLDACCMTHDLCIQHKGNNYLNLECNQNFLDCVAKFTKSGAHSFKGNTCSVNTVVTVITDVIDAAIAAVKIFKKP

>Cann_CAN.G52.3

EKQRREQHKPVRCSKTCVAENCHSLGIRYGKYCGVGWSGCPGEKPCDDLDACCKIHDECIEKNGMTNVKCHEKFKRCIKKVQKSGKAGFARDCPYDVAVPTMVQGMDMAIMFSQLGNSKLEL

>Slyc_Solyc07g014730.2

MVKLSLHFLAFCIIAIFTNLFNSPISIHALNVGVETNAGLSLEKECSRTCESKFCAVPPFLRYGKYCGIMYSGCPGEQPCDALDACCMKHDLCIQHKDNNYLNLECNENFLSCVAKFTKSGSPTFKENTCSITTVVRVITDVIDAAVAAGKIFKKP

>Slyc_Solyc07g032220

MLHGDKSPMGKWVASSFILTILLFFSIAESTNNSQVRCSKTCVAENCNCMSSLSFTLLNSFSHNAYLIYFFFSAIGIRYGKYCGVGWSGCPGEKPCDDLDACCKIHDECVEKNGMTNVICHEKFKRCIKKVQKSGKAGFTRDCPYDVAVPTMVQGMDMAILFSQLGNSKLELI

>Stub_PGSC0003DMG400029964

MASSFQSLKLSLHFLVFCIIAIFTNLINSPISIHALNVGVETNAGLSLEKECSRTCESKFCAVPPFLRYGKYCGIMYSGCPGEQPCDGLDACCMKHDLCIQHKDNNYLNLECNENFLSCVAKFTKSGSPTFKENTCSITTVVRVITDVIDAAVAAGKIFKKP

>Stub_XP006356760.1

MLQGDKSPMGKWVASSFILTIFLFFSIAESTNNSQVRCSKTCVAENCNSIGIRYGKYCGVGWSGCPGEKPCDDLDACCKIHDECVEKNGMTNVKCHEKFKRCIKKVQKSGKVGFTRDCPYDVAVPTMVQGMDMAIMFSQLGNSKLELV

>Dcar_DCAR012001

MLRRTFPAASLHVSIITVIVIFSSLLEIGQSNDNSQEVTCSTTCVAQNCNTIGLRYGKYCGVGWSGCPGEKPCDDLDACCKVHDDCVGKKGMNNVKCHEKFKRCIKKVQKSGKPGFSNVCPLDVVVPVMDQGMDMAIMFSQFGNSKVEL

>Dcar_DCAR014099

MAINCKFAYPARRTEQDRRMLSRNFPARSSVVFIPTILIFSILFQIGESNDNSQEVTCSTTCVAQNCDTIGIRYGKYCGVGWSGCPGEKPCDDLDACCKVHDDCVGQKGMNNVECHQNFRHCIKKVQKSEKRGFSEVCPVDVVVPTMDQGMEIAIMFSEFGNSKLEL

>Dcar_DCAR019790

MAPPAASNTLFLQSFVLMIMCAFHVHTLDLGLHANVDLSLAKQCSKTCESSFCSVAPLLRYGKYCGLLYGGCPGEKPCDALDTCCMKHDACILSKQNNYLNKECNQNLLNCVGNFKKLKGRSFPGNTCNTEDVIKVITVSMDAALLAAGRLPKP

>Dcar_DCAR019830

MPPFKSLLVSFYLIHQLLFFYAPACALDIGIGAKARLSLGKHCSRTCESSFCSVAPLLRYGKYCGLLYGGCPGEKPCDGLDACCMKHDACIISKRNDYLNLECNQNLLNCVQNFKKARGRSFSGNTCKAADVIKVITVAMDAALLAVGHHPKP

>Ptri_Potri.018G132100

MAKECRNSLKLALLVSCSLLVLAFSSFSVQALNIGVQTTDSAISLSKDCSRKCESEFCSVPPFLRYGKYCGLLYSGCPGEKPCDGLDACCMKHDACVQAKNNDYLSQECSQNFINCMNNFRNSGAHTFKGNKCQVDEVIDVISVVMEAALLAGRALHKP

>Ptri_Potri.001G425200

MKCSTELLIQVIIFALFVSHVYIITTSPSLVFLGITFTNSPSIGVQTVDSAISLYCGLLYGGCPGEKPCDGLDACCMKHDACIQSKNNSYLSQECSQNFISCMSNFKTGARTFKGNKCRADEVIQVISVVMKAVLLAGRALHKP

>Ptri_Potri.006G149700

MFVGVRFSVGARVAVALASVLIFLSLFADCANNNDSQEKCSRTCVAQNCNSVGIRYGKYCGVGWTGCPGEKPCDDVDACCKIHDECVEKKGLNNIKCHEKFKSCIKKVHKSGKVGFSRDCTYETAVPTMVQGMDMAILLSQLGSSKIEL

>Ptri_Potri.006G070400

MAKEHQFYSLKLALLVSCSLLVLPFSSFYVQALNIGVQTADSAISLGKDCSRKCESEFCSVPPFLRYGKYCGLLYSGCPGEKPCDGLDACCMKHDACIQSKNNSYLSQECSQNFISCMSNFKTGARTFKGNKCRADEVIHVISGVMEAALLAGRALHKP

>Tpra_TPR.G35489

MVLTQSIKYFVLLCFTFGFNLLTTSVYALNIGAETTGVAVSVSKDCSRQCESSFCSVPPLLRYGKYCGLLYSGCPGEKPCDGLDACCMKHDQCVTVKNNDYLSQQCSQTFIDCMDKFSNTKAPTFKGNTCQADDVIEVIKVVMEAALLAGRVLHKP

>Tpra_TPR.G22824

MTSRNISRATAALALLSLLLSAVADSSSANCSRKCIAEQCDTMGIKYGKYCGVGYWGCPGEQPCDDTDACCMAHDNCVDKFGMTHVKCHKKLKNCLTRVQKSGKVGFSKECPVSVVAPTMIRGMDLAILLSQLGDSVHDL

>Mtru_Medtr6g091700

MTSHINILRATAAFALLTVLLSAVFPSSSANCGRDCIVEQCNSMTIKYGRYCGVGYSGCPGVKPCDDIDACCMGHDDCVGRFGVTHVKCHKRLKNCLIRVQRAGKVGFSKECPVSIAAPTMIRGMDLAIMFSSIGKWQGWP

>Mtru_Medtr6g091720

MTSHSCIISRATAAFALLSLLFSAAFHDTLADSSSANCSKKCIAELCDTMGIKYGKYCGVGYSGCPGEKPCDDIDACCMAHDDCVGKFGMTHVKCHKKFKKCLIRAQKAGKVGFSKECPVSTTVPTMIRGMDLAIMLSDLGDNFHEL

>Mtru_Medtr6g091750

MASSINISRVTASFALFSLLLSTVADSSVANSPVANAPVAANAVVADSSLANCSRTCKAELCDTMGIKYGKYCGVGYWGCPGEKPCDDIDACCMGHDECVDRFGMTHVKCHKRLKNCLIREQKANKVGFSKECPANVAVPTMIKGMDLAILLSELGGNMPDIEKFI

>Mtru_Medtr6g092790

MKINMVLIKYFALLFFTFEFNLLTNHVYALNIGAETTGVAVSVSKECSRQCESSFCSVPPLLRYGKYCGLLYSGCPGEKPCDGLDACCMKHDQCVTVKNNDYLSQQCSQTFIDCMEKFRNTKAPTFKGNTCQADDVIEVIKIVMEAALLAGRVLHKP

>Mtru_Medtr8g101550

MLPVLVFLFFYCTFLSIPVSALNIGVQTTGVTISMNETCSRKCESNFCSVPPLLSYGKYCGINYSGCPGETPCDDLDTCCMNHDLCVKAKNYDYLSQECSQTFIKCLNKFKKSGGPTFDGNTCEVDYIIELLSVVMEGALLAGSLKQALEYWLVFSRN

>Gmax_Glyma.01G002400

MVPTPQLKYVLLLFYCTFAFNLLSTPACALNIGAETTGVAVSVSKECSRQCESSFCSVPPLLRYGKYCGLLYSGCPGERPCDGLDACCMKHDQCVSAKNNDYLSQECSQTFINCMNNFKNSRAPTFKGNTCDADDVIEVIHVVMEAALLAGRVLHKP

>Gmax_Glyma.01G004000

MSRAAASFGILLCLFLAAAAVVNCSDQANCSTTCIAEQCDTVGIKYGKYCGVGYWGCAGEKPCDDLDACCMAHDDCVDKFGMTHVKCHKKLKNCLTRELKSGKVGFSKECPYSRAAPTMIRGMDLAILLSQLGDSVPH

>Gmax_Glyma.07G127900

MSRAAASFGILLCLLLLVAAVNCSDQGNCSTTCIVEQCDTIGIKYGKYCGVGYWGCAGEKPCDDLDACCMAHDNCVDKFGMTHVKCHKRLKNCLTRELKSGKVGFSKECPYSRAAPTMIRGMDLAILLSQLGDFSVPH

>Gmax_Glyma.07G129900

MVPAPSFKYVLFFCCTFAFNLLSTPVRALNIGAETTGVAVSVGKECSRQCESSFCSVPPLLRYGKYCGLLYSGCPGERPCDGLDACCMKHDQCVSAKNNDYLSQECSQTFINCMNNFKNSKAPTFKGNTCDVDDVIEVIHVVMEAALLAGRVLHKP

>Gmax_Glyma.08G028800

MVPSQLSKYGLLFISCTFFLINFLTIPISSLNIGVETTGITVSVSKECSRTCESSFCSVPPLLRYGKYCGLLYSGCPGEKPCDGLDACCMYHDKCVQAKNNDYLSQECSQTFINCMQKFKNSRAPTFKGNACQVDDVIEVINVVMEAALLAGRVLHKP

>Vvin_VIT211s0103g00190.1

MKLAMTLLLCSLIGLIFSATPTLALNIGVQATDGSVTLSKECSRKCESEFCSVPPFLRYGKYCGLLYSGCPGEKPCDGLDACCMKHDACVQAKNNDYLSQECSQNFINCMNSFKSSGGHTFKGNKCQVDEVIDVITLVMEAALLAGRYLHKP

>Vvin_VIT211s0016g02570.1

MLRRCANRTSIAASLATSVVVVVVVAFFFAVADSSNNSQIKCSKACVAENCNSVGIRYGKFCGVGWTGCPGEKPCDDLDACCKIHDECVEKKGLISIKCHEKFKTCIKRVQKSGKVGFSRECPFETAVPTMVQGMDMAILLSQLGSSKLEK

>Egra_Eucgr.C00294

MRYGKYCGLLYSGCPEERPYDGLDACCMKHDVCIQAKNNDYLSQKCSQNLLNCMTNFKNSRGRTFKGSKCQVEDVDVLSIVMEAALLAGRYFHKP

>Egra_Eucgr.C00515

MTMVSPQPSKLALVITLTPLFVLCFSGVPARALNVGVQAADAAISVTSRKCESEFCSVPPFLRYGKYCGLLYSGCPGERPCDGLDACCMKHDACVQAKNNDYLSQECSQNLLNCMTNFKNLGGRTFTGSKCLVEDVVDRQEPEVAMEFKTKDPCTNLECQVMRMNILVYTSVLN

>Egra_Eucgr.C00541

MCSLSLSLSLSLFFVNQCLEAKTKLPGFSASLRPAMTMVSPQPSKLALVITLTPLFVLCFSGVPARALNVGVQAADAAIPVTSRKCESDFCSVPPLLRYGKYCGLLYSGCPGERPCDGLDACCMKHDACVQAKNNDYLSMECSQNLLNCMTNFKNLGGRTFTGSKCLVEDVVDVLSVVIEAALLAGRYLHKP

>Egra_Eucgr.C00579

MTMVSPQPSKLALVITLIPLFVLCFSGVPARALNIGVQAADAAISVGKDCSRKCESEFCSVPPFLRYGKYCGLLYSGCPGERPCDGLDACCMKHDACVQAKNSEFRHRYFQLFFNQSIDITSCSPAHLHGHNLMRMMRKHFHW

>Egra_Eucgr.C00921

MLRPVAAFAVVIAASIAALADCSGDGSQVRCSRTCAAENCDTIGIRYGKYCGVGWTGCPGEKPCDDLDACCKIHDECVEKKGMTNVKCHEKFKTCIKKVQKSGKTSFSRECPYETAVPTMVQGMDMAILFSQIGNSKLEL

>Egra_Eucgr.L01776

MTMVCPQPSKLALVITLIPLFVLCFSGIPARALNIGVQAADAAISVGKDCSRKCESEFCSVPPFLRYGKYCGLLYSGCPGERPCDGLDACCMKHDACVQAKNNDYLSQECSQNLLNCMTNFKNLGGRTFTGSKCLVEDVVDVLSVVIEAALLAGRYLHKP

>Ntab_SS11894

MAFLQSLKLFSLQVLLAFCIIALRFSPISVHALNIGIETNAGISLEKECSRTCESKFCAVPPLLRYGKYCGVLYSGCPGEQPCDGLDACCMKHDLCIQRKGNNYLNLECNQNFLNCVATFTKSGAPSFKGNTCSVGTVVRVITDVIDAAVVAGNIFKKPP

>Ntab_SS4740

MAFLQSLKFSLQLLSFCIIALRFSPISIHALNIGIETNAGISLEKECSRTCESKFCVVPPLLRYGKYCGVLYSGCPGEQPCDGLDACCMKHDLCIQRKGNNYLNLECNQSFLNCVATFTKSGAPSFKGNTCLVGTVVRVITDVIDAAVVAGKIFKKPK

>Ntab_SS9520

MQGGDNSLTSSFILTTLVFSFFLFAIAESTNNSQGVRCSKTCVAENCNSIGIRYGKYCGVGWSGCAGEKPCDDLDACCKIHDECVEKNGMTNVKCHEKFKRCIKKVQKSGKAGFSRECPYDVAVPTMVQGMDMAIMFSQLGNSKLELV

>Ntab_SS1768

MQGGDNSLTSSFILTTLVFSFFLFAIAESTNNSQGVRCSKTCVAENCNSIGIRYGKYCGVGWSGCPGEKPCDDLDTCCKIHDECVEKNGMTNVKCHEKFKRCIKKVQKSRKAGFSRECPYDVAVPTMVQGMDMAIMFSQLGNSKLELV

>Osat_Os02g58500

MPPRSPLLALVFLAAGVLSSATSPPPPPCSRSCAALNCDSVGIRYGKYCGVGWSGCDGEEPCDDLDACCRDHDHCVDKKGLMSVKCHEKFKNCMRKVKKAGKIGFSRKCPYEMAMATMTSGMDMAIMLSQLGTQKLEL

>Osat_Os03g50030

MARGGSFSRLRLRAGVVVAAAAAALLLFAVVAPPAAALNIGLQSAGDGASKAGLCSRTCESDHCTTPPLLRYGKYCGILYSGCPGEQPCDELDACCMHHDNCVQAKNDYLSTACNEELLECLARLREGSSTFQGNKCMIDEVIDVISLVIEAAVVAGRLLHKP

>Osat_Os11g34440

MGDAQRRQLLLVALMLAAAADHSLAGFFGGAPPASGPAAAAADNDEKCSRTCESEHCLGTYAQAPLMRYGKYCGVSYTGCPGEAPCDALDACCMLHDACVQATDNDYLNMLCNQSLLDCVAAVRSPAARIRTFEGNQCNVTDVADEITSLVEAAVFAKRILHRP

>Osat_Os03g15460

MRFFLKLAPRCSVLLLLLLVTASRGLNIGDLLGSTPAKDQGCSRTCESQFCTIAPLLRYGKYCGILYSGCPGERPCDALDACCMVHDHCVDTHNDDYLNTMCNENLLSCIDRVSGATFPGNKCNVGQTASVIRGVIETAVFAGKILHKRDDGQA

>Sbic_Sobic.001G429900

MASVLAFSRCSSLLLLLLATASQALNVGDLLGTAPSGSKDCSRTCESSFCIVPPLLRYGKYCGILYSGCPGEKPCDALDACCMVHDHCVATHNNDYLNTRCNENLLSCLDRVSPAGPTFPGNECGVGQTASVIRGVIESAVLAGKILHKRDDGP

>Sbic_Sobic.001G120400

MERGSSWRRLTVVVGILVCAAVFSPPAAALNIGIQSAGDGASKQQACSRTCESDHCTTAPFLRYGKYCGILYSGCPGEQPCDALDACCMHHDNCVQAKKDYLSTSCNEALLECLARLREGTSTFDGNKCMIDEVIDVISVVIEAAVVAGRVLHKP

>Sbic_Sobic.004G357800

MSPRRALLAILLAVVLASASAAPQPPPCSRSCAALNCDSVGIRYGKYCGVGWSGCDGEKPCDDLDACCRDHDSCVEKKGLHLLACSACVPVVCLVVLVSMMSVKCHEKFKNCMRKVKKAGKVGFSKKCPYEIAMATMTQGMDMAIMLSQLGSQKVEL

>Sbic_Sobic.005G145100

MDGRRRELAVGRHPLQRRCSRRRLLAPLLILLLAVASSQSPTAAGSIFGGGDDDSVSDCSRECESQHCTAPLMRYGKYCGVSYTGCPGEVPCDAIDACCMLHDACVQATDNDYLNLLCNQSLLDCVAAARPAAAAATFQGNRCNVTDVADEITTVVEAAVYARGILHKP

>Zmay_Zm00001d051836

MYEAMSPRRALLAILLAVALASASSAPQPPPCSRSCAALNCDSLGIRYGKYCGVGWSGCDGEEPCDDLDACCRDHDRCVERKGMMSVKCHEKFKNCMRKVKKAGKVGFSKKCPYEMAMATMTQGMDMAIILSQLGTQKVEL

>Zmay_Zm00001d013461

MERGSSWRLTVVAGTLVCASLFSPPAAALNVGVQSAGDDASKQQACSRTCESDHCTTPPFLRYGKYCGIMYSGCPGEPPCDALDACCMHHDNCVQAKMDYLSTACNEALLDCLAKLREGTSTFEGNRCMIDQVIDVISLVIEAAVVAGRVLHT

>Zmay_Zm00001d033683

MERGSSWRLPAVVVGILVCAALFSPPAAALNIGIQSAGDGVSKQQACSRTCESDHCTTPPFLRYGKYCGILYSGCPGEPPCDALDACCMHHDNCVQAKMDYLSTACNEALLDCLARLREGTSTFNGNKCMIDEVIDVISLVIEAAVVAGRVLHKP

>Zmay_Zm00001d052616

MGGGRELAAGRYFPLQRRRSSSRRRPLLVILLLAVVASGQASAASGSIFGGDSASSECSRACESQRCTAPLMRYGKYCGVSYTGCPGEAPCDALDACCMLHDACVQATDNDYLNMWCNQSLLDCVAAARPTAMADTFEGNRCNVTDVADEITAIVEAAVYARGILHKP

>Zmay_Zm00001d028505

MMMASVLAVSRWSSLLLPLLLLLLLLLVATASQALNVGDLLGTAAPSGSQGCSRTCESSFCIVPPLLRYGKYCGILYSGCPGEKPCDALDACCMVHDHCVDTHNNDYLNTRCNENLLSCLDGVSPAGPTFPGNECDVGQTASVIRGVIESAVLAGKILHKRDDGQ

>Bdic_Bradi3g60710

MALLVVFLLLAVVVDLASAVAPPPPPCSRSCATLNCDSVGIRYGKFCGVGWSGCEGEEPCDDLDACCRDHDHCVDKKGLMSIKCHEKFKNCMRKVKKAGKVGFSGKCPYELAMATMTQGMDMAIMLSQLGSQKMEL

>Bdic_Bradi4g16650

MHTGRLLPLLLLLLAAADRSLALGIFGAPPPSSDQDSSCSRTCESVYCSGTIEAPLMRYGKYCGVSYTGCPGEAPCDALDACCMLHDACVQATDNDYLNMWCNQSLLDCVAAVGTAASSAAAAGGAAVVWATFEGNRCNVTDVADEITAILEAAVYAERILHHRSAP

>Bdic_Bradi1g11120

MAHARSGSGRLRVHLLAAIIGLLACCAAALDVGLQYAGDDVSKQQACSRTCESDHCTTAPFLRYGKYCGILYSGCPGERPCDPLDACCMHHDNCVQAKNDYLSTQCNESLLECLGELRDGTGTFEGNKCMIDEVIDVITIVIQAAVVAGRVLHKP

>Bdic_Bradi1g67460

MASFLVASPRCSLLLLLLLLVTASRGLKTGDLVSQGKASADCSRTCESKYCTVAPVMRYGKYCGILYSGCPGEKPCDALDACCMVHDHCVDANNNDYLSTKCNENLLSCLDGVSTAGPTFPGNKCGVGEVAFVIKGVIETAVLAGKILHRRDIGQ

>Gbil_Gb10678

MSQKYCRRLLAAMDLIIPTIVIAISFLSITSIPQAEALNIGLGPVNVTTAPRYILSEYIATDFNFQLKFAPLLRYGKYCGLGYSGCPGEVPCDGLDACCKVHDSCVQVNNGDYLSQSCSKSFLDCITNFIASGAPSFEGNSCDIRLVTDLIYDVIEAALWAGRTFHDP

>Gbil_Gb40267

MLISLGWAHSYAHREIYAANAVAAPYNWLNVQDGSPATAINWFSSVSSSRVACSSCLQLCIMVPPLLRYGKYCGIFYSGCPGEVPCDGLDACCQTHDDCVGHSKYLNVTCNQSLLDCVDAFQSSGQPPFDGNSCDIGTVEDTIYWAIQAGVWIGEGIGDGSSP

>Gbil_Gb39978

MDFGSQMIFIFIFILVISPNAFHSFAFASSSHKTNKTNEPNCSRVCVVQECNSLGIRYGKYCGVGWTGCPGEKPCDDLDECCKVHDQCVGNKGLMNIKCHEKFKKCIKKVLKSGKVGFSEQCPYEVAVPTMTQGMDMAIMFSQFSSPGYEL

>Gbil_Gb40266

MATLRLPFGFQLSLLLVLLAPPACNSASWGDCSTTCKSKFCGEAPLLRYGKYCGIFYSGCPGEVPCDGLDACCHTHDDCIGNSNYLDVTCNQNLLDCMDEFRYSGQPPFDGSSCDIGKIENTIHWAIQAGVWIGEGAGVDPFVEKQLLVTENKTP

>Atri_ATR0564G112

MGLDQASNLASLFLFIAPFAFTFAPNVMALQVDLQTSGLSATLSRRECSSVCESDYCSVPPLLRYGKYCGLGYSGCPGEGPCDGLDACCQTHDNCVQAKNEDYLSQECNQEFLDCISEFRASGRPSFEGNKCDVQEVIDVIYVVIEAALLAGRALHKP

>Atri_ATR0789G151

MQEHKEMSSLQRWRCASPLLISTFVLFLLPLSNALQGPPQPACSKACVAENCNTVGIRYGKFCGVGWTGCPGEKPCDDLDACCKIHDECVEKKGMMSVKCHEKFKVCIKQVKKKGKAGFSKDCPYNVAVPTMVQGMDMAIMFSQLSNPGYEL

>Ppat_Pp3c21 20050

MATLQRRRCPRILAHAGTIVMLLLLELLSPALSLVVNGTIFKGCSRECESRHCQDPIKLRYGKYCGIGYTGCESEVPCDGIDSCCKSHDICIGPNLENYVNRTCNDQLKKCVEKFQDSNMTQFNGSTCSASAIEEVIITSMNIATLGKSVGIPSSLPNTRSYTALVFNSFMVLLLGGW

>Ppat_Pp3c8 920

MGRTMGAAVMFQTFFIAILLPTTASALILNSTTRSCSKHCESINCQSVHKIRYGKFCGVGYSGCPNQSPCDRLDACCKQHDLCVGRNAANFPNHTCRYNLRSCLQQYLSTGAVVYQGSNCTAMTVQNTVMVALESTTFHEHVAPGSAPQAGGAPNPSSGMLIDRITSIVLICGVWASLLWAHELSPALRSVG

>Mpol_Mapoly0058s0039

MEKTLFVAAFCLTLLPLTQALYFNTTPTTCKTVCESEHCKDPVRLKYGKYCGVGYTGCSGQSPCDGLDACCQTHDNCVGSNNLKNYVDTQCSNALLNCVETWDASNSAQFAGNTCPREDVVNTISGVMKLATIDGRLGNAATTATTILSPISILGAVLFIHLFVYQ

>Mpol_Mapoly0124s0052

MKGRYRINSEIQALKSGTMTTMKTLQWVLTFSAAYAAVCIGDDSSYMDVDTVDLLSTHKLCSTDCESKWCYVPPALRYGKYCGIGYSGCDGQDPCDGLDACYKTHDQCIGSKLCNYLNVDCNQALIDCLNIFQASGASQFAGANCSTGTLVGVINAVIEIGVKAGEALNRTGKC

>Crei_Cre02.g095000

MSTYSVLALAALVLLLSGNRAVDAVDAPGREEQPCARSCHTINCDNVGIRYGRFCGVGHGGCPGVKPCDPVDKCCQKHDLCVEKESVFSSKCHKRFLTCLEKHKEKDHEGFAPNTCPYSVVIPTMKAGIEMAMMFTGGLEEL

>Vcar_Vocar.0036s0113.1

MVRPFLWTSILLAFCTMTGATDAPGKEETPCAKSCHTINCNNMGIRYGRFCGVGHGGCPGVKPCDPVDYCCQKHDACVEKHSVLSARCHMSFLKCLDRHVDKDHEGFAPNKCPYSVVIPTMKTGIGMAMMFTSAIKAKAGSEFN

>Afil_Azfis0168ed

MAMAMAFLLLLLPSLLLAVSSHEESSSCSRTCAEDHCNSLGIRYGKFCGVGWTGCEGEQPCDELDSCCKHHDFCVEKKGVMHINCHEKFKKCMKKVESSGKKGFSMKCPYEVAIPLMTQGMDMAIMLGQLGSQTSMV

>Afil_Azfis0128pred

SEGGGQGGECSRECESKDCLFPPLLRYGKYCGIGYTGCPGEKPCDGLDACCQDHDACIGSDPRNYLNVTCNKKLLECALAFEISGAPQFRGATCSKATVCTSMEISMAIAIRLHDQNSKHHNNNNNSTSKSTISASSTTTPLYQLEP

>Scus_Sacus0008.g004084

MAAMAALLLPLFLSILCFCCSSPAHALIVNTTDLSECSRTCEAKDCLFPPLLRYGKYCGIGYTGCPGEAPCDGLDACCQAHDTCIGSDPENYLNVTCNQRFLECVLSFELSGAPQFKVIDDVRVHEV

>Scus_Sacus0014.g006100

MGAALLLFLLSFIAAAESRHQYNSSWLHKKDNEDMNSRFLFLGMVVPEESPTALGIRYGKFCGVGWTGCEGEKPCDELDNCCKQHDFCVEKNGMLHISCHEKFKKCMRKVQNSGKKGFSNKCSYEVAIPLMTQGMDMAIMFGQLGSQSD

>Knit_kfl00026 0180 v1.1 Protein

MGTFSQTTLVCCLLVAGICCGRGVDGAVGTSDKKPRRQPVDSGGLGTGDHGPDCSKVCVAKDCNTMGLRYGKYCGVGWTGCPGEEPCDPLDACCKKHDECCVKHGLLHNPCHKAFKKCMARVRATGKKGFSAECPYSVAIPAMEQGMDLAMMFGQGGMQQFAAM

>Tpli_Thupl.29379784s0005.1.p

MTEIQRFDSLIILSITTIVAVSLSCNGKIPGAQALNIGPVNITSSLASGCSSQCESSFCTVAPLLRYGKYCGLAYSGCPGEDPCDALDACCKTHDSCVQAQNGDYLSLSCNQALLNCIESVRASGAPSFAGSNCDMEQVTSLVYDVIELAIVAGRALHQP

>Tpli_Thupl.29378226s0006.1.p

MNRLVFLVLFLQIVLPYSKRVGSEHGDNGCSRHCQSVYCSVPPLLKYGKYCGVGYTGCPGEDPCDGLDACCQAHDICVQHKNGDLLSQSCNNSLLNCLNTFNATGAPGFSGNSCNVEDVEDLINHVIEAATWAGNIINHP

>Tpli_Thupl.29381603s0016.1.p

MAAALRTQFWPMLFSIVLLSPSCYSFTWGECSSACESKFCSGFDSYIVAPLLRYGKYCGIFYSGCPGEDPCDGLDRCCMTHDHCISNSSYLNVTCNQDLLDCVQAYQNSGDGQFPGNSCEIRDVEKIIKVAIEASV

>Tpli_Thupl.29377455s0005.1.p

MAALRSQFLHMLLFLALLSPYCYALTWGKCSTRCKSKFCKEAPLLRYGKYCGIFYTGCSGEDPCDGLDRCCMIHDNCVGNSNYLSTTCNQALLDCVEAYKNSGDGQFGGNTCKIRDVEKTIKVAIEAGVWIGDGDGSALTSSHPDHTTPNP

>Tpli_Thupl.29377455s0008.1.p

MAALKSQFLHMLLLMAVLCPCCYAVRGTSCSTTCESMDCTNARLRYGKYCGILYSGCPGEAPCDGLDRCCMSHDKCVDTSMYLSTKCNQALLDCVQAYRNSGDGQFPGNTCNVLDVENTITLVIEAAVLIGEVIGDSNYLQHGSALPPI

>Tpli_Thupl.29377455s0004.1.p

MAALRLQFLHMLLSIALFSPCCYALTWGKCSTRCKSKFCKEAPLLRYGKYCGIFYTGCPGEAPCDGLDRCCMIHDRCVGSSHYLSKACNQALLDCVQAYKYSGNGQFRGNTCKIGAVEKTIRVAIKAGLWIGGGAGDGSDLDHPLSSSPNP

>Tpli_Thupl.29377455s0006.1.p

MASLKSQFLHMLLWMALLCSCCYAVPVTSCSTTCESMDCTNARFKYGKYCGILYSGCPGEAPCDGLDRCCMIHDKCVDTTMYLSTKCNQALLNCVQAYRNSGDGQFPGNTCNVGDVENTITLVIEAAVLIGEVIGDSNCLQHGSALPPI

>Tpli_Thupl.29377455s0010.1.p

MAALKSQFLHMLLLMALLCPCCYAVPGTSCSTTCESMDCTNARLRYGKYCEIFYTGCPGEAPCDGFDRCCMIHDKCVDTTMYLSTKCNQALLDCVQAYRNSGDGQFPGNTCNVRDLENTITIVFEAAVLIGEVIGDSNCLQHGSALPPI

>Tpli_Thupl.29377455s0002.1.p

MTVAPLLRYGKYCGIFYSGCPGEAPCDGLDNCCMTHDHCIGDSSYWDVECNQALLDCVAAYQDSKEGQFPGNTCDIGDVEGTINFAITAGVWIGIGIGDGPSPPASEDPVSEDPSSFTPTPTSDP

>Tpli_Thupl.29377455s0007.1.p

MAEAPLLRYGKYCGIFYTGCPGEGPCDGLGRCCMIHDNCVGNSNYLSTTCNQALLDCVQAYKNSGDGQFGGNTCKIRDVEKTIKIGIEAGVWIGDGDGSALTPNT

>Tpli_Thupl.29377455s0009.1.p

MAALREQFFHTLLLILLVSPSPLLRYGKYCGLFYTGCAGEDPCDGLDRCCMIHDNCIGKSKYLSRTCNQALLDCLTAYQNSGDGQFGGNTCKIKDVEKRIRVGIEAGVWIGDGNVFLNFRRIRIPLFEGLMSLLCKLVSIRH

>Tpli_Thupl.29377455s0011.1.p

MAALREQFFHTLLLILLVSPSPLLRYGKYCGLFYTGCAGEDPCDGLDRCCMIHDNCIGKSKYLSRTCNQALLDCLTAYQNSGDGQFGGNTCKIKDVEKRIRVGIEAGVWIGDGNVFLNFRRIRIPLFEGLMSLLCKLVSIRH

>Cric_Ceric.37G023900.1.p

MAGIVQEMRASYKPMRRTAFYIVLFLIASIIPCIYSETAEFQAACSRECAEVQCNSLGIRYGKFCGVGWTGCADERPCDDLDNCCKEHDLCVEKKGMTEISCHKNFKKCMRKVVKLGKRGFSKKCPYEVVVPLMIQGMDMAIMFSELGGLSSTLR

>Cric_Ceric.26G059800.1.p

MALDVIRQPTYNTTFLVFLSLFMGFFSVGLVMDQSELLSRCSTVCESRFCSFPPLLRYGKYCGVQYSGCPGEEPCDELDACCKIHDDCIGNLNNYLNKTCNHNLLECVEAYRASGEGQFKGNTCNIKDVEFSITIAMKIANHLS

>Cric_Ceric.26G060200.1

SRCSTVCESRFCSFPPLLRYGKYCGVQYSGCPGEEPCDELDACCKIHDDCIGNLNNYLNKTCNHNLLECVEAYRASGEGQFKGNTCNIKDVEFSITIAMKIAN

>Cric_Ceric.26G059600.1.p

MFNTLLNMRAGQPQKSLSLSLSLSLSLSLSLWRAAVPAPGHRLTTTCDGMATTTSQALTGVAAFGAAFFVCCAAIILDQSKLLGKCSTVCESKFCDVPLLLRYGKYCGVQYSGCPGEAPCDGLDACCKTHDDCIGNIGNYLNKTCNHNLLQCVEAYRASGKGQFPGNTCNMKVVELSIIELMKIAEHL

>Cric_Ceric.26G059400.1.p

MVVLRVLITLATLSAAFLISCFAIIMYQSEMLGKCSTVCESKFCDVPLLLRYGKYCGLQITGCPGEPPCDDLDACYKAHDDCIGKLGNYLNKTCNHNLLQCVKRHKASGKGHFAGNTCDVKHVELFIINYMKLADFF

>Cric_Ceric.26G059500.1.p

MAPGTRTVLPLLVLLLSAEWFLQALHPSQALLVDPKTAKSLIYGLSSAAVEAHIGCSTVCESEFCSVAPLMRYGKYCGLGYTGCPGEAPCDGLDACCLAHDVCIGSSWENLLNKKCNWELLHCVRAYRKSRANQFPGNTCDIRDVEFNIETAMRIALNL

>Cric_Ceric.26G059900.1.p

MALDVIRQPTYNTTFLVFLSLFMGFFSVGLVIDQSELVSLSLSLSLSLSLAHTTHAHNEVCSTVCESRFCSFPPLLRYGKYCGVQYSGCPGEEPCDELDACCKIHDDCIGNLRFRTDEKPH

>Cric_Ceric.26G060000.1.p

LSRCSTVCESRFCSFPPLLRYGKYCGVQYSGCPGEEPCDELDACCKIHDDCIGNLSTHSLSLSLPHTHTHF

>Cric_Ceric.26G060100.1.p

MALDVIRQPTYNTTFLVFLSLFMGFFSVGLVIDQSELVSLSLSLSLSLSLSLAHTTHAHNEVCSTVCESRFCSFPPLLRYGKYCGVQYSGCPGEEPCDELDACCKIHDDCIGNLRNRCDQMEQI

>Ncol_NycolH01240.1.p

MKPWRSGLLILVIVSPLVVSCLLKSVEGLNIGLQNTALHVSSLSNTGCSRTCESAFCSVPPFLRYGKYCGLLYTGCPGEPPCDGLDACCQKHDACVQAKQSYLDSECNKALLRCLKRFRKSRRKTFKGNTCSVTEVTDIIYTVIEAALIAGGIIHHQ

>Ncol_NycolJ00860.1.p

MTGCCGFNVVIMVMVSSLSLSSLLHRVQGLNLGFQDSALQVYSNAHTGCSRTCHSEFCSVPPFLRYGKYCGIMYTGCAGERPCDSLDACCQKHDACVEAKNNYLDLECNEELLQCVATARDSGAEAFRGSTCLLAEVADVISAVIQAALIAGGVIHH

>Ncol_NycolF00598ed

MDRRVVCGLLAWVALVAVAECLSSQSHEEIGCSKTCVAENCNSLGIRYGKFCGVGWSGCPGESPCDDLDACCKLHDECVEKKGMMNVKCHEKFKNCIRKVKKSGKVGFSRDCPYETAMPAMLQGMDMAILFSQISSPSYEL

>Aang_VJWM01000144.1/sPLA2

MGALALASLLLAIMLPASTALLTDGHPSVSWQRYPFDLRYGKYCGPGYTGCPGEAPCDAVDACCQQHDMCMGPHYEHMLTVHCARQLRNCVEQVPHNAASFPHNNCSLSDVKDLIDDIADSYLVGRADTTSSLADSTKHTTKIL

>Pmar_AEKF_scaffold_2002545

MASRAPTGSSAVACTVMPTPLRLLPLPVLCLLLLLLVAPFACDVAGAPVGARGHDEEVYREKARLAKEQMETEQCARVCAEENCDNVGIRYGKYCGVGWGGCAGEEPCDALDACCQAHDTCAMKFGPQAVDCHKVFKRCMQKVWRTGKPGFSTKCPYEMAIPTMTEGMDLAIMLSEFATGGGGASAFG

>Mend_WDCW_scaffold_2044903

MLPSRCLAYLMVVFTSIHAVTAVTVKKGQGSTQFTQDFAQCSRTCVAKDCNTFTLRYGKYCGVGHGGCIGEEPCDDLDSCCKGHDDCVMEYGMNNVECHLDFKECMQRVAKSKKKGFSKTCPYSVVIPTMMQGMDLSIMLSQAMQ

>Scon_WCQU_scaffold_2057137

MGLQLVRMARVLRLSKIPALSLLICLLGSRICCADEVEARRDTRTEDEGCARVCAEENCDGVGIRYGKYCGVGWGGCPGEEPCDALDKCCRAHDKCAMRYGVQAVHCHEKFKKCMVKVQKNGTEGFSTRCPYSVVIPTMMQGMDLAIMMSQFAGLAQA

>Gkin_KEYW_scaffold_2024598

MRGLSSTSHMLLLLVVVASFSSLSFALSSTQRKGEREDCARECAEIECNSMGIRYGKFCGVGWGGCKGERPCDALDACCEKHDACAMAHGVQSVGCHTKFKRCMMRVKKSGKPGFSKKCPYSVAIPTMMQGMDMAIMFSQFAADH

>Tsoc_A0A2J8ADU7_9CHLO

MGAIWLCALVVLACWAAPGKAVDAPGREETPCAKSCHTINCDNMGIRYGRYCGVGHGGCAGVEPCDPVDLCCKRHDTCVEKSSVFDNRCHKRFLKCLGKHVNKDDEGFAPQTCPYSLVIPTMKAGIEMVMGFAGGGGEL

>Sfal_Sphfalx02G022800.1.p

MAARIISGGVGDVRGSNIIVFRLTLLSIIVLLQQFLLLLLLLLLSFCTSSSALIFNGTVGSCSNTCESQNCDDPFDLRYGKYCGIGYTGCSGAVPCDGLDRCCMVHDRCIGSQLTNYLNKTCNSNLRSCIEAFKNSGNATFAGANCSASDVETVAITAMDIATGSTGFSPPMPFSFGWVTLSVCGLSVLLLLWPSLMVNQFRS

>Pdru_ZZEI_scaffold_2005308

MGGRRRRQLQQSSKLAMFFFHALHALFFSAAPVHGLNVGIQSANSDLTTNKDQNCSRKCESENCAVPPFLRYGKYCGILYSGCPGEKPCDALDACCMTHDACVQAKNNDYLSEECNENLLNCIGAVKARRGATFKGNKCLVEEVVDVITVVIEAAVIAGRVLHKP

>Swal_JKAA_scaffold_2171307

DPFDLRYGKYCGIGYTGCAGQGPCDGLDQCCMTHDNCIGSNLQNYVNRTCNSQLKDCVTQFKNSNAPLFTGSNCSATDVETVIIASMDTATLGSPGSPSPPINLRWIEAIVFVLSVVLCFGW

>Mneg_A0A0D2ND28_9CHLO

MPINADATKLIGKTPTVCFAHWLCCPVLNLPGDMRAAAAQAHDKCYDKNGYFNCDCDKNILECLQGVFISNGPRREDQNAFKTAAMLYFRNAPCRSSGGSWSFFRLSVRGTTCQGPEAQLNNGEQQPGSGGGGRQTRSIMPFRPRALLAWAVLVVLMLSGVQARHRDDAPSWPLYGNWCGPNHGGDAAPIDPLDTCCQAHDKCYAASGYLNCTCDEKMVSCLNDVKIPQASEFKGQRIFRAVAVMYFKLSLCRTAGAWTWMRLATIASSAVASR

>Mneg_A0A0D2MCZ1_9CHLO

MYGNWCGPHHGFEDGAQPPIDALDACCQAHDQCYVDKGYFDCSCDDTIAACLARVSVPAGFTYNEQRLFKGAAMMYFQNSLCRSDGQWVLEHAFQKLRRKL

>Csor_A0A2P6TPW6_CHLSO

MSTRRFHSPAKALLVALVSAALVAAGAAPVREQGHKAAAAAAALSRRNLRGMESSIATPAGGNLTTKGLAEIVDSVLTGAANAWTYGVENLAEHFSGKCNYGNWCGEDCMGKKGTPIDTLDEHCETHDKCLALETDSCLRCMCHVNLLQSIDQMLADKGCAIDSSNWASDSCQSDESVKEAPTIAMGIQYRMSQDSCDSYAWKDSCNTAPASDNYNSEAMYRYEVTIGTSCNSGAGTDGYVQAKFTDETGAYISTGDLDNSGNDRQTCSVDTYAVGLFKTKVLMNGNTKLEVYFRPAGLFPDWEPEYVQVVRDDGSDAVSAKFCSSGVIAQEGWSSAASAAAAAAAVTRRGLRGIETSVELDNNVTTKGFSESVDSFVTSLGNDLTYGLSNVFEETTATKVLMNCDTTCQVYFKPKWAFPDWQPEYVRVSRSSNNDQTYSVRYCMDGILSEEGWYTFYCCD

>Atha_AT4G29070

MNFGLPSISWFGSISTKKDVAMIDSVTPTTTSLLEQPEQEKATTFLLKQPEKEKGLFDIKIWTWSSFSSVLPWSANASDGKQKPTTINRGLKRHALSRRSSRSNGVNTVYRFRPYVSKVPWHTGTRAFLSQLFPRYGHYCGPNWSSGKDGGSMVWDQRPIDWLDHCCYCHDIGYDTHDQAELLKADMAFLECLESNKRVVTRGDAQVAHFYKTMCITGLKSILIPYRSYLVKIQYGQNLLDFGWIVSNLSKRSWSFQKN

>Brap_Brara.A00815.1

MNFGLPSIPWFNSNSSKKDVTMVETVTSTTSLLEQQDDQGQSLFGIKIWTFSLGSVFPWAATSRDGKQQKPTTINRRLKRHAVSRRSSRVNTVTTVHRFRPYVSKVPWHTGPRAFLSQLFPRYGHYCGPNWSSGKDGGSPIWDQRPIDWLDHCCYCHDIGYDTHDQAELLKADVAFLECLESNKRVVTRGDAQVAHFYKTMCITGLKNILIPYRSYLVKIQYGQNLLDFGWLVNGLSKRSWNFQKN

>Brap_Brara.H01454.1

MKFVLPSVSWFNSNSSKKDVAMVETVTSTASLDQGKGLFGIKIGTWSLGSVITSGDRKQKPTTINRGLKRLAVSRKSSRLNSVNTVYRFRPYVSKVPWHTGLRAFLSQLFPRYGHYCGPNWSSGKDGGSLVWDQRPIDWLDHCCYCHDIGYDTHDQAEMLKADMAFLECLESNKHVVTRGDAHVAFFYKTMCITGLKSILIPYRSYLVKIQYGQNLLDFGWIMSNFSKRS

>Ptri_Potri.001G275300.1

MNFEEFKVNTPWFKMTSNKDSGTNPKPATAVLSMGCMMGQPGQQPRFDIKFWGWSLLAIVPWAINAKDKIRAPDTINKKLKRHAQSRGVVDSGRGNPLRFRPYVSKVPWHTGARAFLSQLFPRYGHYCGPNWSSGKDGGSLLWDKRPIDWLDYCCYCHDIGYDSHDQAELLKADMAFLQCLERPHMATKGDAHVAHVYKTMCITGLKNMLIPYRTHLVKLRSGQPLINFEWLSKVKWRRWNLQKT

>Tpra_Tp57577_TGAC_v2_mRNA17181

THSVQTKAFALQAKHETFGIDPKDPTLSVQTKAFASQAKQESSGIDRKDPKLSGWPLSFLSLFPWANNAGEKFQRPTTINKALKRHAQNNQNVVGKDNLASPLRFRPYVCKVPWHTGVRAYFSQLFPRYGHYCGPNWSSGKDGGSLVWDKRPIDWLDYCCYCHDIGYDTHDQAKLLKADLAFLECLENRHVMRTKGDPHVAHLYKTMCINGLKSFLIPYRTSLVSLQQSGGTLIQFGWLSNLRWRSWNYQKE

>Egra_Eucgr.B03735.1

MNFEFLNRTPWFGRAHPDNDLGSSFGSSTVLMEQPKRKDGFNLKLWGWSILSVVPWALSSRDRTGMPPTVNRQLKRRAQSGGIAQNSSKVMAVRFRPYVSKVPWHTGPRALLSQLFPRYGHYCGPNWSSGKDSGSPLWDQRPIDWLDYCCYCHDMGYDTHDQAKLLEADLAFLECLEKPHYRTEGNAHVAHLYKTMCTTGLKNFLIPYRAHLVKLKSRQPLIDFGWLSNMRWRSGNIQKN

>Vvin_GSVIVT01037285001

MNFRFPSNVPWFGANSNNDLETAVKTAGVLSKSTKERAFFDVKLWGWSLLSIVPWAISARDKIWKLSTLNKELKKPSRRYRTVEYGSRPSPVRFRPYVGKVPWHTGARAFLSQLFPRYGHYCGPNWSSGKDGGSLLWDKRPIDWLDFCCYCHDIGYDTHDQASLLKADLAFLECLERPQMSTKGDPHVAQLYKTMCITGLKNVLIPYRRHLVDLRSEQSNILFGWLSNVKWRGWNPPGES

>Dcar_DCAR_024408

MNFGFLNNIPWFGVNSNSQNENTVLSSTSMVSVSSSRHASIDINENVAVSSSTSMVSVSSARHASIDIRQWGWSLISVLPLAFSANEKIKMPTTINKKLKGQAQSPSKYGVGGSSNVYSTPRFRPYVSKVPWHTGARAFLSQLFPRYGHYCGPNWSSGKDGGSPLWDKRPIDWLDFCCYCHDMGYDTHDQAELLKADLAFLECLERPHMATKGDVHVARLYKTMCISGLRGILVPYRKQLVRLQNQQLSINFGWLSSITRKGWNFQKS

>Gmax_Glyma.07G232000.1

MDFLGKIPWFNAQVNTDLATNSIPIETFTEQPKQELGNDPKLPFLSLFPWGNRAGEKFQRPSTINKELKRQARCGNGVGKDGEATPSRFRPYVCQVPWHTGVRAFLSQLFPRYGHYCGPNWSSGKDGGSLVWDRRPIDWLDFCCYCHDIGYDTHDQAKLLKADLAFLECLEKQHGSTKGDPHVAHLYKTMCVNGLRNFLIPYRRNIVNLQQFGQPMIQFGWLSNLRWGGWNFQKTHRLSSLGGSTVS

>Gmax_Glyma.20G041000.1

MFGCFLLFLLAGYKKIWFCGSYLCTLVTLVFSTMDLGFLGKIPWFNAQLNTDSGSNSVPIDTFTEQPKQELGNDSKLPFLSLFPWVNRAGDKFQRPSTINKELKRQARRRNGVGKDGEVNPLRFRPYVCKVPWHTGVRAFLSQLFPRYGHYCGPNWSSGKDGGSLVWDRRPIDWLDFCCYCHDIGYDTHDQAKLLKADLAFLECLEKHHGSTKGDPHVAPIYKTMCLNGLRNFLIPYRRNIVNLQQFGQPMIQFGWLSNLRWGSWNFQKTHRLSSVGGSTVS

>Stub_PGSC0003DMT400058494

MDFSFFGNFPLFKPHSANDMASTVASASTLMQTPKQNAQFDAKFWKWTVFSVLPWAKGAEGNIQMPTTVNKKLKRRRPFREGVDSLARTSTIRFRPYVSKVPWHTGPRAFLSQFFPRYGHYCGPNWSSGKDGGSPIWDRRPTDWLDFCCYCHDIGYDSHDQAELLKADLAFLECLEKPNMSTRGDPHAALLYKTMCTSGLRNILIPYRQQLITLQSKQLSFGFGWLGGIMEPAKCLKDRFVWLQK

>Slyc_Solyc01g008780.2.1

MDFSFFGNFPWFKPHSANDMASTVASTSTLMQTPKQNAQFWKWTVFSFLPWAKVAEGNIQMPTTVNKKLKRRPSREGVDSLARKSAIRFRPYVSKVPWHTGPRAFLSQLFPRYGHYCGPNWSSGKDGGSPIWDRRPIDWLDFCCYCHDMGYDSHDQAELLKADLAFLECLEKPNMSTRGDPHVALLYKTMCTSGLRNILIPYRQQLITLQSKQLCFGFGWLGGIMEPAKCLKDRFVWLQK

>Atri_evm_27.model.AmTr_v1.0_scaffold00063.74

MINNDGPLDRHYLRCWRREFRHFYLIDFARTLHSSAMNFNFLKILPLVHFHPAIVKEPEHKTPPTTIARKIENGKVIEASRNASTVINVWGRSLHSFIPWVYNLKRKEESQGPKAVEPRYTRPFFRPYVARVPYHKGARAFLSLLFPRYGHYCGPNWSSGKDGGSLLWDKRPIDWLDFCCYCHDIGYDTHEQEKLLKADLAFLECLEKPMMSTKGDVHVAYVYKAMCITGLRNMLIPYRKQLLKLQEKPLSIESLNIFRGKGDTFQHT

>Sbic_Sobic.002G401900.1

MEVTSSSSSPSAPPPGSSKPALRLNPAAVLLRRLPTPTPTTATPVTASEPPARPGGASNPLAAFLSSLIPFWRERRWGPPKQPAHPAASSAAARRAAEQEAEAEARQLVGCAVPLFRPYVAQLPWHGGTRAWLSKLFPRYGHYCGPNWSSGKEAGSVLWDRRPVDHLDFCCYCHDMAYDTHDQAQLLRADLAFLRCLEGSRRTPARDGIAAAVIYRAMCIFGLKTILIPYRTNLVRLQTGPNYADFFADFVKRVASSSGRPTGGEKQRL

>Zmay_GRMZM2G166971_T01

MEVTSSPSPSAPPPASSKPALQLNPAAVLLRRLPTPTPTAATPVTATAPPARPATASNPLAALLVPFWRGLRRAPKQPVHPASAAAARRAAEQQEAEAEAEARQLVGCAVPLFRPYVAQLPWHGGARAWLSKLFPRYGHYCGPNWSSGKEAGSVLWDRRPVDHLDFCCYCHDMAYDTHDQAQLLRADLAFLRCLEGSRHTPARDGIAAAAIYRAMCIFGLKTILIPYRTNLVRLQTGPNYADFFADFVKRVASSSGRPTGGEKQRL

>Osat_LOC_Os07g46420.1

MEAAASPSPPPSRASPLRLNPAAFLLRTTTTTTTVQPTTSADAPPPPPPTRQTAGVDRLISFLSSLIPRRGQRAKQPTSPPPTAAAAAAMRRAAEREAEAERQLVGCAVPLFRPYVAQLPWHGGARAWLSRMFPRYGHYCGPNWSSGKEAGSVLWDRRPADHLDFCCYCHDMAYDTHDQAQLLRADLAFLRCLQSSRQTPARDGIAAAAIYRSMCIFGLKTILIPYRTNLVRLQTGPNYADAFADFVKRVASSSGRPTGGNKQRM

>Mpol_Mapoly0002s0074.1

MKIDVLNLFPAINLPTFPSFGSQGVSQDPAGNVRTVTDVEETGCQLPPFVPYASKIPWHSGPRGLFSRMFPRYGNYCGPNWSSGREDGSLFWDKAPIDRLDHCCYRHDMGYDSYEQADLHRADLRFLGCLEKIDKQGHKAGDSPFAEAYRKMYILGLRNFLIPYRKFLLQKVDEKTRKRLTEEEIFGKDRRKNPMS

>Ppat_Pp3c9_17190V3.1

MKVDSLKFLNTAHSLIFAKDDQENQAITLPEAEDVQKQVEISQEGGCRVPPFVPYTATVPWHTGSRNLFSRFFPRYGNYCGPNYSSGRESGSLHWDKPPTDWLDYCCYRHDMGYDTLDQAKLHDADKKFLNCLQNIPESEKITSLGQTYRNLYVLGLERFLIPYRDFLVKKIDEGKRSRETPPEMIVQQQRNGANLNRSNIN

>Ppat_Pp3c15_13470V3.1

MKLDGSKFLNAAESLLFRKNGRTNQVLSNGILLSDAEAVGNHVQTSKQGGCPTPTFVPYTATVSWHTGARNLFSRFFPRYGNYCGPNYSSGRESGSLHWDKPPTDWVDYCCYRHDMGYDAYDQAQLLDADHHFLKCLQKIPETAKISPMGATYQNLYILGLQSFLIPYRGFLVKKIEEAKRRKVRGAVGIVKELRENGAPSMNRSDMNSWQVLTSLMNLTLTLL

>Mtru_XP_013442423.1

MNLGFLGDIPWVKPRSNQDSAIHLVQTTAFTSQVKHESSGIDIDPKFTPDSATHLVQKAQQESSVNDPKLLGWPLSFLSLFPWTNKDGEKFQRPTTINKELKRHAQNRENVVGKDNMATPLRFRPYVCKVPWHTGVRAFLSQLFPRYGHYCGPNWSSGKDGGSLVWDKRPIDWLDYCCYCHDIGYDTHDQAKLLKADLAFLECLENRHIMRTKGDPHIAHLYKTMCINGLKNFLIPYRTNLVSLQQSGRSLIQFGWLSNLKWRSWNYQKE

>Ntab_N90mRNA_175635

MDFSFLSNFMSLKPQSAKDMASTVVSTSTLMEVPKQKAQFDAKFWKWTLFSFVPWAKGSEGNIQMPTTVNKKLKRRRQSRESVNSLARTSTIRFRPYVSKVPWHTGPRAFLSQFFPRYGHYCGPNWSSGKDGGSLVWDRRPIDWLDFCCYCHDMGYDTHDQAELLKADLAFLECLEKPHMSTRGDPHTALLYKTMCISGLRNILIPYRQRLISLQANQLSFGFGWLSGIMEPAKCLKDRFIWLQK

>Ntab_XP_016464201.1

MDFSFLSNFLLFKPRSAKDMASTVVSTSTLMEAPKQKAQFDAKFWKWTLFSFVPWAKGSEGNIQMPTTVNKKLKRRRQSRESVDSLARTSTIRFRPYVSKVPWHTGPRAFLSQFFPRYGHYCGPNWSSGKDGGSLVWDRRPIDWLDFCCYCHDMGYDTHDQAELLKADLAFLECLEKPHMSTRGNPHTALLYKTMCISGLRNILMPYRQRLISLQANQLSFGFGWLSGIMEPAKCLKDRFIWLQK

>Knit_GAQ78528.1

MRFPSSLSLASFPPRNSDADGTDPPEDMRGPRHSNQSTRSGAPERAAKLAAEVVSEAETMTGCHLESFVPHEYTAQLDWHKGPRAALSRLFPRYGRYCGPNYSSGRDDGSKTWDQAPVDWLDNCCYRHDMAYDSHDQSVLLEADVQLLRCLRSRPRKADGKKVELVYPWGEAYEMACQQGLQHFLIPYRTVLIRLKERKQREEEKTRPAQHRRKDDRRPTLDL

>Cbra_GBG79578.1

MVTWPGVDPSIWFGVVRTQGVRWPWWSEEGDGDHGISRPQVGGGNTWRWLSWSADADVADPTSASDSASSSLSSMSSPWSLFSWDWITDRKGEAIGVRATSQLAALVSTSPSTPSSYSSSPLPWSLLSWDRITDWKRQAIGTGAVSQPALPALPACGHVSRREAGGGGSSGLTVSPKLRGVCAGCEFRDAGGGYDSTPFVPFLPYIPYTQKLPWHTGPRAVACRLFPKYGNFCGPNWSSGRDCGALRWDKGPVDWLDHCCLVHDIGYDSHSQVDLYKADCQLLVCLRSPPRGRDGKPFRLSLWGEFYRALCIRDAVPIKVIKTVSTIASGEGNVTTSARVRVANTPTGPRVFPCKVLAEAPPGFEDKPTTIGEATFVVTILPPQVLDVRP

>Gbil_Gb_15712

MNFDFLQNLWPPKFVLTPKQDAKVETASISRAEEKSDYEKSENSKLHQDRFGIWEHFLRMNKVETDSVPKTENYSNYGKTETSKPHRDRFGIWDHIQQINLPWNTKSDKQAKKTNANELEIVESGCAIPSFTPYMAKIPWHTGARAFLSQMFPRYGNYCGPNWSSGKDRGGLLWDKKPIDWLDFCCYCHDIGYDTHDQAEMLKADIAFLECLEKIRMPPKGNPQVAEAYKSMCIAGLRKVLIPYRKQVLKQMHGRQFLVSKINGLNGRKDTVQRQ

>Afil_Azfi_s0031.g024464

MATINTNISIAIATESPSPPTISTTKPNPNPNPTPPLLTSFAASASASASAPAPAPASSDHGDSNEPPLHPPFTPYAAHLPWHTGPRAFLSHFFPKYGHYCGPNWSSGRSHGPLLWNLRPIDSLDYCCFCHDIGYDTHDQARLYRADLDFLECLSRIPDSASAKKKKKKKLWNPPRHESPLAVFYRTACIIGLKTVLLPYRRMLLQKIEEEEEAAAAGVKKKKVVI

>Scuc_Sacu_v1.1_s0009.g004581

METSVAPKLLTGIARETPHPPSLHPSPNRSVSFSPPNPSAASSSDASGCRLPPFSPYTATVPWHTGVRAFLSHLFPKYGNYCGPNWSSGRSGGSLHWDKRPIDMLDHCCFCHDVGYDSHDQARLHKADLAFLECLSKMPPSGKAPPSGNVFVAEFYRSACITGLRTFLLPYRNFLLQRTVTKNDIVVDLKPGA

>Mend_WDCW_scaffold_2045767

MTSPNSKDDHEQHTTSTSDYPSIEVKNSHERSDSLAGSNKSTDGSSGSGDGGCLLPPFLPYTATVPWHSGPRALFSRLFPKYGHYCGPNYSSGREGGSLHWEKEPLDVVDYCCYRHDIGYDTESQAGLLKADEQLLKCLENAPLIASKRGEPSLSQAAEAYRVICIFGLRRFLIPYRTLTARQEQDYLLKVADKRRPDSPASRALKDSNEVLGDAEQQGLPPPAGLPLSNSGLGQRQSAASIGLQEHSWPSAQEKEGR

>Rsub_GBF94301.1

MATTRLARALFLVAVLAVHGAAAGRPAAARAAPASGSNANSTAPAAAGGRSAPATAGNVTASAAAGGRRPGAGRRGPGGERPVAPGGRPATAAAEMQSMPIYGNWCGPRWGSGQPIDAVDACCQKHDLCYDAHGYFDCACDHALLECFANVHVPASQERTRAFKNVAAVYFRNTLCRTGRNDWLWARDAGPLVAAKLRALKNATADAAAGAGAAVAAAPAGGAAAAAAPLVAHAANATLPPQLRAAARLAQAAAAAIKNVTTAHLATKAGHAVAA

>Ppro_GHP11153.1

MSAASSSSSSSVSPAQTTTQSSSSSSSENNNNREMDALSTFVPTMRVCGNYCGPGWCGGTYVQEGPQCPFDQLPTDCNDACCKVHDQCCREDALEHKGCDQALVQCITDCAPSFDPGGEDPDPGPYAPGPGTKCDEMTSNWVRRVMQSAINLDVGQCGGFATATVTDAPPGAHTHGAGAMPLTAGGYHAWQIMGKGVLSYMFAFTQTTSWSVLTNTGTVALVNRDNFEHMARNEPYNAAAPDLCCDSRNVKSTVYEQGVRIDPCELSGDGTCEVFVVARCDNSYTDCAGRYSFDFLADKVGEGGLREVRAQV

>Cric_Ceric.31G013200.1.p

MDLKPVGASMKAPSPPNPLSFPSRLGFLPSLVSSSTEEVPSLSEIPSSTTSSPSSSSLTSSADKEAVHELPPFIPYAAQVPWHTGARAFFSQIFPKYGHYCGPNWSSGRSSGSLFWDKPPVDWLDYCCLCHDIGYDSHNQAKLYNADLEFLECLQKIPPPVQKRLHHTTPLHPGSKYSDPIWAGFYRNMYISGLQKFLLPYRRMILKDMGTQVRKEDSI

>Cric_Ceric.06G048200.1.p

MSEPSPSPKSSSTRDVLLSSTSTSSSSPKKTALEEVNDGVHQIPPFSPYSAQVPWHTGIRAFLSNIFPKYGNYCGPNWSSGRSHGSLLWDKPPIDWLDHCCFCHDVGYDTHNQEKLYKADLEFLECLQKKPYPMHKQQRIVSLYPRTHEHNNRILAEFNRMLYIKGLQIFLLPYRRVLLKIMKESS

>Sfal_Sphfalx15G080800.1.p

MGVMMKVDAVKLLNSAQNLVLRKKESGPATGNEVSQNSTTIGLQLPNDEDSTAMLPDAEQVIKFDENQQAGAGPAGCALPEFIPYTQTLPWHSAPRNLFSRFFPRYGNYCGPNWSSGRESGSLVWDKKPIDWLDHCCYCHDKGYDSYNQADLYNADVEFLNCLQNIPEVERKNLPGAYRNLYILGLQRFLIPYRQSLLKRSTEKGSKQRQDLSHNPMQALDQNISKIESDRTAS

>Swal_JKAA_scaffold_2181091

MEVGSLQLKGGPRGLGRKLGARNCCGNADEDHGCAVTALFTPYQAKVPWHSGTRAALSKLFPRYGNYCGPNYSSGRDSGSLLWDKPPIDWLDYCCFVHDIGYDSYSQLDLYKADLAMLDCLNKMPVDDKPKSSQDVAREAYRRLAIIGLKNVLIPYRRALLRDQKLDRADELNESSSKE

>Aang_VJWM01000193.1

MPGAGQESVSRFATMVGTLLPTSIALPGMQRLESGVAQQAGSYSKPLAVALPQARQLDGLKPGGVSKAAASRATVSPARTTASGIQYLVRVASGWRSGSEEEKEEGDGSAESDSKPLERASKESDLKALFVPYSSKVPWHEGPRAFFCWFFPKYGNYCGPNWSSGRGQGSLTWDKPPLDHLDYCCYCHDMGYDSHDQRDLLKADLQLLRCLETMPERDRW

**Supplementary Data S2.** Promoter sequences of selected sPLA_2_ and PLA_2_-like genes analyzed in this study.

>AmTr_scaffold00010.89

CTATTTAATAAAATCTGAATATCCTCCTTGAGAATCACTCCATTTGATCCATTGTTCCTCTAAAGCTCAGCTCTCTATGTTGTGTTTTTCATTTTTAAAGACATAGAACCCCATCCCCCTCCTCAAAGACAAGGGCAAAGATGGCAAAAATAATTAACCCACAAATCAAATACACCCAAGACACCCTATTTAAAACCCATATAATCATTTGCCCAGATACTGAGAACTTGCCAAGAAATTAAGGCTCCTTTTTGAGTTACATGCTTCTTTCCATGTGCTATGTTGAACCCGGGAGTAGGGACTATTCCTTCTTTGTACTGGCCAATTCATTGCAATGTATTTTGCAATTAGGAGTCAAGTCAAGAGCCTTAATTTTCAAAGAAAAGGTCAAGAGCCTCAATCAGACCATAATATCTTGTATTTTAGTCGCCATGCTGGTCGTGCCTCCATCACCTTACCCCTAATGTTTACCATAACTTAATTCTATACATATTATTTGGTGAATGACCATTAAAAATTTATAGCTACGGTCGCTTCCTAATGAAACTTCATTTTTGTCCTCTTTTGTGTTTTCGTGAATGGCACGTATATTATGCTCCCTTGGGAGCATAGTAGCATAGTTTGTTTTTCTGCTCCCTCGCTCAAAGACCTACTTGAGAGGATTGTCTTTCAATCTGAAATTTTTTGAATTATTTAAAGATTACTAGATGATTATTCTCAAAAAAAGTTTTCTTGAAATTTGGTTCTCCTAACGCTTGGATCTCACAATCTAAAATTCGGATGGTCCCTAATGACTATGTTTTTTTAAAATGCATAGAGGGTACAATTAAGATTTTTTTTTTAAGAATAGATTGTACGTTGAAGAGCTCAAAACTTGGGTTCTTAAAATTAATTCTAGGTTATGGGTGGCCACAGGTCGGTTCAGGTCGTGTCCAGGACCTGATCCGGACCCAGAAAAAATGATATGAGACCAAGACCTGACCGGTCCCAAGTTCATCGGATCTAGTTCCAGACTGGGTCGGATTCGACCCAAAATCTAAAGATATCTATGTATTTAACTATATTTAAATATATTTCATATATTGAGACTTGAAAGATATTATCGGACCCGAACTCCCAGACTGGAATAGTGTTCGGATCCAAAATTTAGACCCGGACCCAACCTGCCGGGTCCAATTTTTAAACCCATATCTAGACCTAGATTGGGTCTATCCTTTGTTGCCAACCCCCTATTCTAAATGAGGTTTTACTTGAAGGGAGTAATCTTTTGGCTTTTGCAAAACAATGATGAAACTATGGGGAAAAGTCCAAAATAATAATAATAATAAATAAAAATATCTAAGGGCACTTGTGTTGGAGGTGAAATGTTTCGCAACCTACCGTATCCCAAAAAACATTGGGCAATTCTTTCCCATATTTCGCGTCACTTTTGTATGGTGTGACTACACATGTACCGCAGTGCTAACATCGTGATATTTCAACTCCAAGAAACAAATAAGAAAGTGGGAAAATCTTAGCCCGTAGATTGTTTTTCGAAATGATAAACGGTGAGATTTAACACATGGATGAACGATGACGACGGAGAACTACATCTAATCTCCACCATTCATCACCAAAAACAAGCAGTCTATGTCCCATTGTGTACCATACATAATACATCACTCATGCTAGGAAATGCACTACACACCGCTCATGTATGGTAGACTCTCCTGCAAAGCTCAATAATATCTATTTCAGATCCTCAACACCAAAAAGACAAAGTGGATATCACGAGCTATTACACGACAAAGACCTTCACCCCTCAAATTGATGCCCCTTTTACCCAAAATCGAAGGCTCTTCTAATCCAATCCAAGCCGTCAATTCCCAGAGAAGCCCATTTTTAAATCCGTGAAACCCAGGCTTCTGAACTTCAGCAAAGAGAGAGAAAGTTGCAGAGAGAGAAGCCCAAACTTTAAACACAGAGAGAGAGAGTTTTGAGCTTTTTCACCGACCATTAAA

>AmTr_scaffold00036.177

ACAATACAATAATGTGCTCCGCAATATGCTACTAAGATACAAGAGTCAACGTCAAATATGACCAAAATGATTTTTGTGTACAAGAAGGACATTTACGTTTAAAAAAATGAACTTTTTCCTTAGAGGGTATATGCATAAAAAATTCTTTTAAAAAAAAAAGCGAAAGAAAAAATTAGGTTGAAGTAAGTTGCATAGAAAATGTGGTGTTGAAGTTTCTCAATAATCATAAAAAAATCCAAGTCTTCTAATAGAGAGAGTGGAAGTAAGATATGCTCCTTATACATGCTCTTAAGTTAAGAGGGAATTTGAATTTCTTAATGAACAAGCGACTCAGTATGCACGTACTAATTTGTTGAGACGTAATAAATAAAAGAAATGTATATAATGAGACCAGGGGATTTGAGTTTTTTTATTTATTCTTAATGCAACGAGCGATTCGTTATGTACGATCCAATTTTGTGAGAAGTAATAAATAAAGTGATATATGATCATTTATTTATTATATAAAAAAGTCATAATTATTATTTGAAGAAACTATTAAAAATATATCTTAATTTTCATCTATTTAGAAAAAAAAAAAATTTAACTTTCATAAACCTCATCCACATCTTTTTTTTCTTTAATTTTGTTTTTTTTTTTTACCAAAATAACGTTCATGCATACGTAAAAACTAACTATGTTTTAGGGCATTTTAAGTACAGTAAAAACCAACTCATGTAATACCGCATATTAACCCATGTATTAGGCATTTTAAGTATGTAAAAACTAAACCATTTACATCGGCATTTTAAGTACCATAATTTTGGCCTTTTTTTTTACCAAAATAACCCCCACCATTTTTTTTAAATTTTGACTTTTTTTTACTAACCCATGTTTTAGGGCATTTTAAGTACCCTAAAAACTAACCCATGTAATACTACATACTAATTCATGTTTTAGGCCATTTTAAGTATCGTAAAAACTAACTCATGTAATACGGCATTTTAAGTATAGTAAAAACTAACACATTTAATATTTTAAGTACCCTAAAAACTAACCCATGTTATACGGCATTTAAAATACCGTAAACCTTTTTTTACTGTTTATATTTATCTCTTTCATCTTGTTTATATTTATCTCTTTTATATTTTAATTTTTTTTATATTTATCTCTTTTTTTTCTCTCTAATTCTTTTTAATATTTATCTCTTATTTCTCTCTAATACTTTTAATATTTATCTCTTATTTTTCTCTAATTCTTTTTTATCTCTTATTTCTCTATCTAAATCTTTTTTCACTCTAACTCTCTTTCTCTTTCTAAAGAGATTTGTATAATGTGTGCATACCTTACCATCCCCATAAAAACTCCCTTCAACCCTATCCCCTATGAAAATTAAGTAGAGAGAGAAAGAGAGTTAATGAGAGAAATGAGTGTTAGAATCCCTTGGAATCATGCTTGTTGAGTTGATGAAGTAAGTAGAGAGAGAAAGAGAGTTAATGAGAGAAATGAGCGTTGGAATCGCGTTTGATGAGTTGATGACCTAGTAGAGAGAGAAAGTGAGTTAGTGAGAGAAATGATTTAGAGAGAGAAAAAAAAATTATAGAAAAGAATTAGAGTAAGAAAAAAAAAAATCATAAAAAAAAATTAGAGAGAGGAAAGAGAAAAATATAAACATGATAAAAAGGTTTACGGAATTTTGAACGCCATACAATATAGGGGTTAGTTTTTATGGTACTTAAAATATGTAATACGACTATTATGAATGTTATTTTCGTAAAAAAACACTCAAAATTGAAAAAAAATAAAAATGTAGATGAGATTTATGAAAGTTTTTAGTTTTTTTTATAATAGATAAAAATTAAAGTGTATTTGTGATATTTTTTTTTTATTAACTACTCGAAATTAGGTCGTTAGGATAAATAAGAGGCACAAAATAAAAAATAAATTCTTTTTTTGAATGTTGTGCCACAATAGGATTTACTGTGTATAAATCATGAATACGTAACGTTGCGAGGTTGTAG

>AT2G19690

TCTATTTCAGCCGCCGGTTCAATTGTTTTTGACAGATAAACCGGTTTGGTTTATACCGATCCGGTTCGGTTATTGTCGTAATTATAAGGCAGTGTAGATGAAATTTGGAGGAAAAGATTCTGACGCTGGTAGTGGTGAGTGGTGAGCACGGCCAAAAGGTATGGCTTGCTTACGATCACCGATTCATTCTTCTTTTATTTCGTTTAAGATTCTGATATTTTGGTCTAAAATTCTTCTTCGCTCTTTTGGATTTTGGTTTTTAGGGGTTTAAAGTTTGTTTTTTTATGTGTTTGAGTCTCAGATCTAATTTGAAATCTCTTCACTGCGATAATGCTTTTTAGGTTTTGAATTGCTTAATTTTTCCATAATTCGGATTCGTTCGATTTGTTAGTCTGGAATTGAGGACTAGGGTTTTAGTGATTTAGCTTGATATGTGTGAAAAGCCTTATGCTTTTGGATGCCTTGCTTCTGAAATTACATTTTTTCAGTTTCCTGTTTTAGTTATTGAATTCTATCGTGGAGGTGTATAATGAACCATGCTATTGTATATGATGAGCTAATCTTTCTTGTTTAGGCTTAGTTTCTGAGACTCAATTGGTAAATGCTAAACTTGGTTTCTATGATTTTGTAGGATTTTGGTATTGAAAAATGGCATCAAAATTGGTTCAACTTCAATCAAAGGCATGTCAGGCTTCAAAGTTTGTGGCCAAGCATGGGAATTCGTACTACAAGCAATTGTTGGAGCAGAACAAGCAATACATCCAAGAGCCTGCAACTATAGAGAAGTGCAGTGAATTGTCTAAACAATTGCTCTACACTCGTCTTGCTAGGTCATTACCATTTTCTTCTCTCTTTATCTCATGGACATATTTTCAATGGCCATGCTCAAACTATCTTTTATGTATACTTTTGAAACATTAATCTTTAGAGAATGCTGATGAAATGTGCAAGTTGAATTTGCATTACTCGCGAGTAGGTAGCCTTTTTTGCCTGAAAAAAATATGCTTGAGAATTTGTTATCCTCTAGTAGTTTTTGTCCTCGATGGAATTGAGCTTGTAAATATCCTTTGATTAGGATAATATATGTGCCTGTCTTGTTTTTCCTGTCTAGATAACACATTATAAGCACCACATTGTGTTTTGCTATCAGTTGAAGAGTTCGGCTTTCTGTGATACGCAATCTTGCGTCTATAGCTAATACTCTTCTTACTAACTACAGCATTCCCGGGCGCTACGAAACATTTAGGAAAGAAGTAGACTACGCAAAGAACTTATTGAAGAACAGAGCGAATCTGAAGGTGGAAGATGCAGGAATCGCTGCATTGTTTGGCTTAGAATGCTTTGCTTGGTTTTGCGCAGGTGAAATCGTCGGCAGAGGATTCACTTTCACCGGCTACTACCCTTGAAATTGAGACAACAAACAACCAGGTCGCCGGAATATTCTTCCTGACGCTTGTAACTCCCTCCATATGATTCCTCACTTGAACTATATATACTTCCTCGAATCCATTTATATTTTCAGTTTTTTCCTGAAACAGAAGTGTTGGAGCTAAGGGAAAAAAAAACATTTTGGTAAAGCTCTTAATAAGTTGAGAAAAATGCATTTGATACAAGATTCTTCTTTAGATATTTATTTTATCCACAAAGTTTAATATCTGTTCACAATATTTAAAACATCAGAGAATTATTTTTTTTAATATATTAAGTGATTGTTAATTGTCGAATAACTTCAAAAATAGAGGAATATTTGAAAAAAATGTTATTTTGAAGAATTTATGATATTCACAAGAAAATGATTTTTGAGTAAAAAACTGTTATTTTTATTTTGTTTGAGAAACTGATATCATAGTTGAAGTAAAAATTTAATTTTGCAGCTGATAAATTCAAAAATAGAAATATATAATACATTTATTTGAGTGGACTTAAGATAAAATATTGAATTAACTTCAAATCTCAGCGAGAGAATATTCAAAAAAATAATAATAAAATTTTGGTATGTGCGAGAAAGCAGGAAGACCAATGGGATCTTAAGGAGACCCAATTGGAATCTTTAGCCACAATTTCGA

>AT4G29470

AAAGAAACGACATCGTTCCAATCGAAACGCATATTACGCCGTCGTTCTAACATAAAGAAGAGAAGTATTACGCAGTTGTTGTTGAACCAGTGGCCCGAAATCTCGAATCGACTCATCTCTCAGCTCACCGGTGCTGCATTATCAAAACTGCAGGAAGGTACGGCGAGTTTCTGGTCTCTGACCGATGTTTCTCAATTGGGTCGAGGCTTTTGCTCCTGCTTCTCTTGTATATGCTATGCTTTGTGGTTTGACTTTTAGATCTATATCTGTGATCTCTTCTTGCTTCGATAACGATTTGTTCGTTCTAGGAGTGACTGATTTGTAATTTTGATTTGCTTGATTGATTCACACTGTAGTTTGTTCGATCTATCGTTATGGAATATAGGATTTGGGGTTTTAGGCTTTTTAAGTCTTTAAGCTTGATTTGTGTGAATTCACTGAAACGTCTCTTGTTTGCTGTAGTAGGGAAATTTACAAAATTTTCAATGTCAAAATTTGATTAATTTAAATTTTATGATTCAAAGAATCTCTTTTTGTATTTGAAGGATTTGGTTGTGAAGATGGCATCGAAGTTGATACAAGTTCAATCAAAGGCATGTGAGGCTTCAAAGTTTGTGGCTAAGCATGGAACTTCCTACTACAGACAGCTGTTGGAGAAGAACAAGCAGTATATCCAGGAACCTGCCACTGTTGAGAAGTGCCAAGAGTTGTCTAAGCAGTTGCTCTACACCCGTCTTGCTAGGTCGATATCTCTCTTTTCCTTCTATATGAACATTTCCCTGGTCATAGTTACCTTATCTAGATTCTGGTTAGAAAGAGACTTTTGTCAATAAAGCATCTTCTTTGAGGTATAAGATAGCCAGGGAATGCTGAGCTCATACAAAACTTGGCTGTTCATGGAACTGAAAATGGCATACTATGAATCCTTTATTCTATCCTCTTATTTCTATGACCTCGTGATGCTATCTTTTTGTTGAGCTGTTTGTCTTGTTTAATCTGTTTTCATAAGTTGCAGGATTGTGTATTAAAGTAGTCAATCTTAAGTTTGTTCGATTATTAATCTCTAATAAGCCCATTTGCAACGATCATTCCAGTCTACTAAGTCTTAGATTATACAATAACCTGACTCTTAATCTTTTACGGTCCATATTTTCGGGTACAAAAGAGGTCTCAGTATATTGCCTTTTTCTTGATGACGGCTTTAGAGCATGTCCTGTTCCTTAATCTTGAAAAGTTTATACATATGCGAGTTCTATCACCACCTTTTGTTTTGCCATGAATTGAAGAGTTTTGGTGATTCACATTTTGCAATCTTGATTTTGCCCATAGTGTTAAGTATCTAACACTCTTCCACTCCTTATTACAGCATTCCCGGACGCTATGAAACCTTCTGGAAGGAAGTAGACTACGCAAAGAACCTATGGAAGAACAGATCCGGTCTGAAGGTAGAAGATGCAGGAATCGCTGCATTGTTTGGTCTCGAATGCTTTGCATGGTATTGCGCAGGTGAAATCGCCGGCAGAGGATTCACCTTCACAGGCTATTACCCATGAAGGAGAGTAACAATAACTTGAAGATTGTGTAATCCCCTCCGTTTTTAGTTTTCATGATTCTCTCTTGAATCAATTTTTTTTCCAGTTTATCCCTGAAACGGAGGCTTTGAAGCTGAGAAAAATCCATTTTGCTATAACTCTTTGATAAAGGTTGCTCTCAATAAGAACAAAATTTCCCAGGAAATTGCTTTCACTATAATGTTTTTGTCCTTGTTTAATATCAATATCAGCAATGATACAAAATTTGAAACCATATTATCATTTGCAATCTAATAAATATCGTTTCTAAAAGATTATGTAGATCTTCATGATACTCTGTTCTTAACATCCATAATTTTTATTTTTTGATAAAGAACGGCCATAAAATTTGGTGGTTGATTGGTAATCATGAAACGTCTTCTTTTTTTACATATGAGAAAGAGGAGGGACGAGAAAGCAGGAGGACCAAGAATGGGACCTTCCATTCCAAAGAGACACAATTATAATTCTTGGGGTCAGAATTTGAAAGAACAAAACAACAAACTTCTACTGGAGAAGGTAAGATAGAAAC

>AT4G29460

CCATAATTTTTATTTTTTGATAAAGAACGGCCATAAAATTTGGTGGTTGATTGGTAATCATGAAACGTCTTCTTTTTTTACATATGAGAAAGAGGAGGGACGAGAAAGCAGGAGGACCAAGAATGGGACCTTCCATTCCAAAGAGACACAATTATAATTCTTGGGGTCAGAATTTGAAAGAACAAAACAACAAACTTCTACTGGAGAAGGTAAGATAGAAACATGATCCGCGGTGGTGCTTTGACACATGTTGCTTTAGGCTTAACCGTCTTCCTCCTCCTTGCCGTCGTTCACAGCCAGGTTGATACATATCACTTACTGTTCTTTGTTCATGTTTGTGCGTTGTGGTTTATTCCAAATGATGGTTATTGCTAGTTCTTTCTGATGGGTTGGATCAAAATGTCAGGAAAAGTGCAGCAAAACCTGCATTGCACAGAAATGCAATGGTGAGATTTTCATTTTTCTTATGCATTCCTTTGAACATGGAGAACATTTCTGAAGCAAAACCTATCTCTGTTTCAGTTCTCGGTATTCGCTATGGGAAGTATTGTGGGATAGGTTACTTTGGATGTCCTGGAGAGCCACCTTGTGATGATCTTGATGATTGTTGTATGACTCATGATAATTGTGTTGATTTAAAAGGTAAACCAATCAAGAATATCTACGTAAAACTATTCTATAAACAGAGAAACACAAAGTGAAGTTAACATCTTTTAACTACTTTGTCCACTACAAATCTAATTGCACTCTGCATCTTGGATCTGAGAATGCTGCATTTGGTGCTTTTTTACAGGTATGACTTATGTTGACTGCCACAAGCAGTTCCAGCGTTGCGTAAACGAGCTTAAACAATCAATCCAAGAATCTAACAACCAAAAGGTTGGATTTTCCAAGGAATGCCCTTATTCAACAGTGATACCAACCGTGTACAGAGGAATGAATTATGGCATTTTCTTCAGTGGGATAGGTAACTAAATAAGCACTGGTTTATTAATAACACTATGAGAATCACTTATAGACTTCGGAAACCATAAGCTAGTGATGAAACCGAATTACTTATGTTACATTGTTTTGTGACATTTTGTATTATAGCACTTACTATACTGTCTTCTAATGTTACACTGCAAATAGCTTAGTTTAGCTAGTGTTAAAATCTGATAAGATGCGAATAATCTCGCGCAGGTAATATCATTATACCTAAGAAGCCAGCAAGTGCCGGGCCTGTCGTGGAGGTGGATCTGGCACGGAGTAAAGCGGACACAAAAGATGGCCTTGGAACAAATCAAGGCCCTCAAACAAAAGATGGCTCCAAAGTCTCTGTTCCGATGAACCCTTCTCCTTCTTGATCATTAGGTGACTCATAACTCTCAAGTTTTGTAGATTATTTAACTTTGTTGTCTTATGAAAAATGATCAAGATCTTGAATTCTCTCAGGTTCATGATTCCCATGTATTTTATACATTACATTAGTTTCTCTTTTACTCTAGATATGAGAGGATTCAAAACGAATTCATACTTATATAAGAAATCAAATGTAGAAAAGAAGAAAAAGTGTATATGGATTTTTTTTTTCTAATAAATCAAACATAAATTCTGTTGTTTCTTGATGTAAAAATCAGCAATGATACGAAAATCGAAACATATTAATCTGCAATCTATTTGTAACAGATTGCATTAAGAAAATCATTAGCCGTTGCGAATACATAGCTTAAACGTGTTCTGTTCCAAGAGGTAACAAAAATGATACTCTGTTTTTACCGTCCATGATAATGCTTCTTCGAGAAGGAACGTCCATAAAATATGATGATTTGATTGGTAGTCATGCAACTTCTTCATTTTTTAAATTCTTGTGTACGTAAAAGAGAGAGAGAGGGGAAGGACAAGAAAGCAGGAGGACCAACCAAAAATGGGATATTCCATTCCAGAGAGAGCCAAAAATAGTTTGGGACAGAATTTGAAAGAACACAAAACCCAAAAACTTCAACCAGAGAGCGAGATTTAGAAAG

>AT2G06925

TGAAGCTGAGTGGGTTTACTCAGGAGATAAGTAGTTGAGTGAGTGTACTCAAAGTTTAAAAGTTGGTTAAGTTGAATCAGGTTCGGAGGTGGAGTCAAGAACTAAGTCACGTCATTAGGGTCAAGGTGGAGAATTGTGAATAAGTGACCCCAATGCTATTAATATCCTAAGTATAGGAAAGACTCGAAACAAGAAAGAGGGTATTCGGTTTTGGGTTAGTCAAATTTAGCACATGGGACCCACACGTGAGTACGAATAAAGTTGAGAATCAGCTTTATAACCCGAGCTCTGTTTTCTTCTGTGATTGATTCGTTTTGTAACAGAAAGTAGAGAGAGAGAGAAGAGATAGATTCTTGTAACCGAGTAACAATCTTTGTGGCTTAGTGGATTCCGGAGAAAGCCTCCGGCGAGACGTAAGGTTCCGATTTGGAGTCTGAACTCATAAAATCGTCTGTCTTATTTCTTTCTTGTAATCAAACGAGTGACGAGAGTGATCGGGGAGCTATAGTGAGTTGCGAATCAAGTCCGATTTGTACAGAAATGCTAGTAATTAGAATTATTAATTAATTACTAATAATTAATACCAATGATATTCTTTGTAAATAAGTTCCAACTTGAAGATTTATTTCATAAAAGTGTTGCAAAAATGTATATATAGATTTGATTTGAAAGTTGCCTAACTTGATTTTAAGCAAACAATTTATTGTTCCATGTATTTTCTCCTAATCTAGTATTCCAATCAAATCATGATAAGCTCATGGCTAAGAATTGGATTGACATAATTTAAATAACAAATGTATAACTAACTACTCATTTGCTTTAAAAGTTTTCAGATGAAAGAGAAAAATCGATGCAAAAATTAAACAGTCTTTTAGGTCAACACTTTTAGGTTTCTCTCTTTGCATTTGTATATAGCCATATTGGAGAGAATGTCGTATATTGAATTTTTTGGTATATCTAACGTCTCAAAAGACCATTACTTACAAAAATATGCATGTCATTATTAATGTTATCACTTTATCATAAAGCACAAAAAGACCATATCGTGTAGACAAAAAAATGTAATAATAAGAAAAGACTAAGTTACAATTACTAAACTAAGAAAGATGAAGTAGCAAACAACTAAGTTGCAATACACAAAAATTTCTAGTTGTTATCAAATAATGACTACTAAAAAAATAGCAAAATAAGAAAAGTAAAATGAAAATAGTGTTTGATACATAAACATAAGTTTTTATATTTTTTTTCTTTTAAATGAAGATATTTAGTTAAGAGTGGAATAAATGGATGATTCAATGCAATGTGAAAATTTTAATTTTTGAACACTAAGTTCTGTTGTTTTTTAATGATATTAATAAGTTGTTCAAAAAAAATTGATATTAATTGAGAATGACATTGTAGCACTCACTAGTGTCATGATGTTTTATTCATATGTAGAGAGTCGGCCAACTTTCATTTATATGACTATATAAGATGTTATACAATAGTTTTAGGTAAACAAAACACTCAAGGAGATATATTAAAAAACTCTAAATATTCCAAATAAATAAATAGAATGAAGTATAGAGAAATTTCTTCCTAAAGTAAGAATTCATTAACGAAAAATCAAAAAGAAATATACACCGTGATCTGATTTAATCAAACGAGGTTTCCTAATTAGATACTACTGTCCACTTCATGATTGCCCTTTACCTACCAATTAACCCGGTTAAGCAATTTTGTCTGATAAAACTTTATTGTGTTATTATCAAAAAAGACTATTATAGTAATGTTGATTGTTGAAAAAATAAAGAAAATAATTGATCATGAGCTGTATTGCATTGTAGTTCAAGATATAATTAGTTTAATTATGTGAAGAAAAAAAAAATGTTATATATTGTTTGATAGTTCAAGTCTTCAAGATATTTATGTATGTGGGAAATATGGAAAATCACATTATTAGCCATACGAAACAAATGAGTCACGAGACATAAGAAGACAAAGAGACAGAGTCACAGACTATTCGTCTTCTACCTTCTTCTAACTCACTTCATTTTCACCAAAACCAACAAATATATTCTTCTCACTTTCCGAGCTTTCCAGTTCAACT

>LOC_Os02g58500

TTATTGCCAGCACATTTTGGAAAACAGAGATCGATAGCAAAAGGTCATATATCAAATATCGAGAGCATAACCGCCAAAGTCTCGGTGCAAAATTAATTAGCCATGCATTTCCCACATTCATATTATTGTTAATGAATCTAGATATCTAGATTCATTTACATCAATATGAATGTGGAAAATGCTAGAATGACTTATATTGTTAAACGGAGGGAGTAATTAATTTATGATGCCTACTTCTCCTTATGATTCATGCCATCCAAAAACCGAAAAAATGTTGCCTGTGGACTAAACAGATCTTGCAACAACTCAACATAACCTCAAACATGGCTAACATGTATGTATCGAAATGCCTTCTAAGATACTGAAAAGTCCAGATATTTAGGCCTATTAAAGTTTGAATCTTTTCATTCAGAGTAACACAATTACTAGAAATCATCCTGAGCTACAAATGTTACTCTGAAACTGCTGTCCAAAAACATACTGTACTACCTATAGCAGTTGCCTTAAAAAAAGCCAACTTATTTATGCCAATGTATCAGCGGAGAAATTGGATGTGGTATTAACAGTAAATCGATGTCCAAAACCCCTCACTCTCCCAGCATTTTCCATTGTCCAAGAATATTAGTGACCCAGTAGAAATTAGTGAAATTTGTGCTTGGGATTGAAGCGATTTGAGGAAATGCATACATGTTGTGATCAATACTAGGAGAATATCTGAGGCACCGTAAGGAACACATAAAAAAAAAAGTACCTTCCTACTCCAAAGTGGAGGTAGAGATAACCCTGGCACCAACTCTATCGAGCTGAGCAACAGTAGCTGTTGCTTTGAGATTGCCGGCATTGGCGAATGCCTCCACCATCCTCCGGCCAACCTCCTCGCCCTTCTCCTCCCCGTCAATGACAGCCACAGCAGTTGGCCCTGCTCCACTGATGGTGCAGCCCAATGCCCCAGCTTCCAACGCCGCGGCCTTGACCGCAGCCATGCCAGGAATCAGCGGCGCCCTGGTTGGCTCCACGATGCCGTCGGAGGACATTGCGGAGCCGATGAGGGTGGCGTCCCCTTGCAGCACAGCGGCGACAAGCGCGGCCGCTTGGCTGGAGTTGCGGACGTGCTGGTGGACGGCCACCTGTTTGGGCAGCGCGGCACGCATCTTGCTGGTGGGCGCCTCGAAGTCGGGCGTGACGAGGACGAAGTGGAGGCGGAGGGCAGGTGGGGAGGAGAGCGGGATGAGGTGGAAGGGGTCGTAGCTGCGGACGAGGACGAAGCCGCCGAGGATGGCCGGGGCGATGTTGTCGGCGTGGAAGCCACTGACGGCTTTCTCGGACTCGAGGCCCGCGAGGACGAGGTCATCTTGGTGTAGGAGGGAGCCGAAGAGGGCGTCAACGGCCTTGGCAGCGGCGGCGGCGGAGGCGGCGGAGGAGCCGAGGCCGGAGCCGAGGGGGAGGCCCTTGGTGAGGTGGATGGAGACGGCGTGGGACTTGACATCGAGGGCGCGAAGGGCAGCGATGGCGGCGACTCCGGCGCAGTTGCGGAGGGGGTCGCGGGAGAGGCGGTCGGCGAGGGTGGGGCGGGAGGGGGAGGTGACGGATGCGATGGCGACGGTGCCGGGGGGCAGGGAGGGGTCGAGGGTAGCGGTGACGGTGTCGCCGAGGGAGAGGGAGGCGTCGGCGACGGCGCAGCCGAGGAAGTCGAATCCCGGGCCGAGGTTGGCGACGGTGGCGGGCGCGAAGGCCGTGACGGAGTTGAAGGCCGGAGCGGGGTCGGCGATGGCGACGGCGACTTTGACTCTCCGAGAGACGCGGACAGATACGAGCCCAGGCAGCGTGTGGCGGGTGGAAGGGAAGCAGGGAGCCGGAGAGGGCGCCGCCGCCGCCGCCGCCGCCGCCGCCATTTGGTACTCGGTAGTGGTGGGGGGTTTGGGTGGTTGACGAGTGGGAGTCGAGTCGACCCGTGTGGTGGAGTGGGTTTAGGTAACTGAGACTGACAAGTATGGCCCACCAACCAATCGCAAATTCACCATCCCATCCAATCTTGACGACG

>LOC_Os03g50030

AGCTTATCAATTATCCAAAATAGTTAATGAGTATATATATCATTAACGAATTAAAAACCAATATAAAAAACTACAATAAAAACTAAAATAAACTATGAGCTGTGATAAACACAACAGAATTTTAAAGCTTGTTTAGAGAGTTTAAAATTTTGAGAAGCTAGTAAGAATCTGAAAAGGTTGAATTTTCCAGTATGACTTTTACTTCATTTTCTAAATTCTAGCTTGTAACAATCTAAAAAAATTGGAATTTTCAGCTTCTAACTTTTACTTTGTTTTTTAAATTCTACAACTATATATTACTAGAAAAAATACCCGTGCGTTGCAACGGGTGAAGTCTATTTTAATCTTATTATTGTTATATGGTTTAGTTAAGATGAAATTCACTGTGAGAATTCGCTTGGATATATATATATATATATATATATATATATATATATATATATATATATATATATATATATAGAGAGAGAGAGAGAGAGAGAGAATCATGGGCTGTAGTTAGGACTTCGGTCGTCTCAAGTTAGCATGCGAGTTTTTTTAAACATATTTTTTATATGATTACTTCTGTATTATCAAAAGTGAACGATCTTAAAAACCTACCCATACACGGATAACGTATCAAAATACCAGCAAAAATATCTTCAATTTTTATAATAGATAATATTTCAGAGACTGAATGACAATCTAAACTATTGAGTAAAGTCCATCACCAGTCCCTAAACTTGTACCGCTGTGTCATCCCGGTCTCTAAACTCGCAAATCGACCGTTCAGGTCCTCAAACTTGTTCGACTGTGTTATCCCGGTCCCTAAACTTGTAGATCACTCGTTTATGTCCTCCAACTTGTTCAGTTGTGTCACCCCGATCCCTAAACTTGGATTTGAATATCATCTGGGTCAAATAGGACGGTCTAAAGACTTTATATTTAAAAATAATTCATAACTTTTTCATGTGAACTCTAATGAAGATAAACTTTATATCAAACTTATAGCCCTCGACGCGATCTATAACTTTGTAGTTGAATTTTGATATAAAGTTTGTCTTCATTAGAGTTCACATGAAAAAGTTATGAATTATTTTTTGATATAAAGTTTTTAGACCGTCCTGCTTAGGGACCGGGGTGACACAACTGAACAAGTTGCAGGACCTAAACGAGTGATCTGCAAGTTTAGGGACCGGGATGACACAGTCAAACAAGTTTGAGGACCTGAACGGTCGATTTGCGAGTTTAGGGATCAGGATGACACAACGGTATAAGTTTAGGGACTGGTGATGGACTTTACTCTAAACTATTTAAAGGAGTTTTTAACTGTTGAAGATTCAAAGCTCATCAAGAGCTCTCTTAGCGCATTTTTTTTACTAGGACCTATTAACTTAACATTTTAAGTCAGTTTTTTATATGATTTATAAATTAATGGATTTAAAGTTTTAAGTTAATTGGTGAAGTCATATCTCTATCTTACATAAACAAAAATAATTTTTTCAACCCAGTTTTTGGCTTAATAGTGTATAAGAGTGACTTATAGCTTTAGAAGACCAAACAAAAAGCTATTATTTGTTTAGATTTGGAGTTTTCGACTCATAAGTTGATTTATAAGCATAAATAAGAGGGATTTGTTTAAACTGGATCTAAAAATACACCACGATTACAAGCGGCGTCCCGGTAGGCAAAACAACCTAAAAAGGCGCCTGCGCACATTGCCTTGCTGGACGACGCTGGTGCATGCACGCACGCACGGCATTGCAGAGCCCGTGACGTGAGGGCGAGCTAGCCAACAACGCTCACGAGCCGAATACAACCAAACGCCGCTCCGTCCGGCCACCTCCCCTTCCTCCTCCATCATCTCACCGGGTGCGCTTCCCCTCCCCTTCGTACAACGAAGATTTGCTATTCGCCAGGGCCCAACCACAGTTATAGCTCACGTAGCCGCTACAGGCTTCTTCGCCGCCTCGTCTCCTCCTCGATCTCCCTTCCTCCCTAGAAAATCTCTCGGATCTCGCCGAGAGCTAGCGGCTGGCTGGCGGCGGTGGCGCGGTGGCAGCAAAGTGTACGCTTTGCAGTTGCTGCCGGCGCGGCGCGGCAGCGGTGACGACGAAGAGGGAGGAGGAGAGAGGCTGTGTCGGGTTTGTGTTGTGTTTGTGTGTGTAGTGTGAGATCTGGAGACGGCCGGGAGG

>LOC_Os11g34440

GCGGGTGCGGGCGTGTGGCTGGGGGGGAGGTGGGTTGGGTTCGGGGGGGACACGTGGAGGGGTTTTAGTGGGTGGGAATGGTGTTTGCTGTGTGTGGACGGTGTGGGTATGATAAAGATAGGTTTAGGTTTTTTTTTTTTTTGCGCTCTTTGGTGATTTGGGGTTTTTGCTATCTCTCTTTTTTTAAAAAAAAGATAATATTGTAGTTCTGGTTAGCTGGAAGTCTGTTTTTAGATTTTCATTTAGTGCAAATGTATATGTATATTTTTTAAAAAGTTGTTTTATAACACTACAATTATAACAACTTTTATGTTGGTGGAGTTGTAATTTATGTTGTGATTGTCTTAAAACGCCATTGCAATATTGGCCATAAACTTTATATTATGTATAGTAGGATGTTTTCTTCATGCGTGCTTGTAATATACTCCATCCGTTTCACAATGTAAGGCTTTCTAGCATTGCCCATATACATATAGGTGTTAATGAATCTAGACACATACATGTGTCTAAATGAATGTGAGCAATGCTAGAAAGTCTTACATTGTGAAATGGAGGAAGTAGTAAGGCTCTCCTATTATTTTTGCTCCTTTTATCGTTAGATTGGCTTATGGCTTCTTCCTTGCTTGTTAATTTTCCATAACCTCAAGTAGACTGGGTTATTTATTTATATCTACCACTAAGAGGAGGAGGCAGGGCGAGGAGAAAGATGTGTTGGCCGTAAGGAAGAGGGAGTAAAAGGTGAAGTAGTCTATCGTCCTTGGAGGCAGTGCCGTGGACAGCGTGATGACGATAAGATGAGGCGGTTTGCAGCTTTTTTGTTTTCACAACAGGGGCGTGGGAGAGTAGGAGCCCAGGCCTATTTGTGAAAGAAGGTTTGCAACATCTCCATAAAAATGGAATGGCTCATGCATATTTGTTTCATGACGTGACACGATAATTTTGCAAGAAACTTTAGTGATTCAGTGTTCTAGATAAGATAGCTAAAACTAAGCCAACTATTGTTTTATAGCGTCCGTGTAAAACAATCTTCTCACCTCTAAAATCAGATATCCAACACCCCTAAGCTATTCAAACTATTCAATTTACCCCATGTTTAAAGTAGCTGTGTTTGCCTATATATCATTGACGTGGTGAAAAAGTCTACCACATCGGCTAACATCAATGTTTGGCGATGCCACAAACAAAGTTATGAGATAGTAAGAAGTAAAGAATGTCATATTTGAAAACAAAAATGAAAGAATATGCTATATGGACCGCACCATAAAAACACAGTTCTGAGTTTGTACTCTTCATATATCTTTTACTAAAGGACACAAGCTGACTTTTATAACAGAATTTTTACTAGGATGAATGAGCCAAAGTGGACACAAGATCTTGCCTGCCATCCACATGAAAAACAAAACTATTCCTCTTCTTCTTCTTGGATTAAAAGATGATGGATGGGCCCAGGAAAGAAACCTGGCCTAGAAAGGGCCGGCCGGGACCGGAGCATCCGATCTCCTGTCAAAACCGACGCCCATCATGATCCATGAACAATGCTACCCTAATCAATCAACATGCATATGCAAGCATGGCTAATGGTCCCACGGAGAGATCGATCACCAACCCGACCGGCTGACCCCACATCCATGCATGCAATGATGCAAATCAGCATATACATGAATACATCCGTCCATGCGGTTGAGCTAACAAAATGATGAAACGAGATGATCGCCAGATTAATCCATTGTTTACATGCAGCCAAAGTACAATGACAGTTGACAACCTTCAATCTGCAGGCAATAATTTACACTTTTCTTTTAAATAAACCTTTTTTTTCCTCTTAGAAATTAAACCGAAGCTGTGAGCAAGAGCACATCATGGTCAGTTCTTGGAAGTCTTTTGTTTGACACACACTTGAATTGCTCCCATTTTTTCCCAACCAATTTGCCACCTACCATGCCTATCTGCACGCACCCGTCGCGCGGTTTATTAGCCTCTTTTCTCCTGCGAATCACGGCGAGCTGATCAGCGATTAGGCGAAGCAAACGTACCAAATTATA

>LOC_Os03g15460

TGCTGGGGCAAACCCGCAAAATCAATTCCGTCTTAACTGATCCAAAGACTTCAATTCATCAGTCATCACACAACATTAGCTCTCTGTTTTCTCAACTCCCTGCATACATGGCTGTGTTTAGATCCAAAATTTGGATCCAAACTTCAATCTTTTTCCATCACATCAACCTTTTTTTTGAACGAATCAATGAGCGCCAATTTCATTGAATAGAGAAGGAGAACTTTAGAAAGCGAAAGGAAAAAAAGAAAGAAGCTAAGACCTTTGGTCCGAGCTAACTTAAAAAAAAACCAGCTATTAAAACTTCTCGCTCCTGCTAAAGCCTAAGTTTTGGCCTCTTCCTTGATCTTGGCTAAAAGAGAGGTAGCTGAAAGCTCCTTCTGTTGCCATCACATCAACCTGTTATACACACACAACTTTTCAGTCACATCATCTCCAATTTCAACCAAAATCTAAACTTTACGCTGAACTAAACACAACCTATATTCCCACACGTGTATCAGATTATAGTCAACAGCACAAGAACATATAATCACTCTAACATGAGTGTACTAATACATATATAGTACTAGTTTAACTCTAGCTGGTAGATAGAGATCGAACGAAACATGAGATGGGATTTACACGAAACTGTGAGTTGGGTCGAAGCATCTCCGGTCTCCACCTGAACTATGCTCGTAAACTTGCTTTCAACTAGTATTGATATTGGGTGGCTGGGTGAAACGTTCCATTCGTTCTAGAGGAGCCTGTACTCTAGGCCCAAACAATTTGAAATGTGCAGCGGTTGGGTGGGCTCTGCGGTTACCGGGTCATTTCTTCAGAGAGACGCCCAAACAGCCACAGTTTGATACCGCTTCTTGTAGCATTTAAGTAACCAAGGCTGGTGCAGGCTGGGCTTTCCATATGGATGTGGTCAAACACTGAAATATGTCTCATTTTTCTATATGTTGTTTGGTGTGCCTTGAGAAGTTGAGATGGCAAAAAGGATAGAGATCCTGAACTCTGATCATATTAACATTTGCATGCCAAATAGACGTATAGGTCAGAAGTTACAAAAGAACTGCAGCCACTTGTCTGGTGTTCTCCTGTGACGGGCTGACGGCAACTCTTGAACTGCATTTGCAAGTAATGGCATTGTCATATGCCTTCAATAATAATATTGGCTCAGTGAGATTGGAGTGTACCTATCACTGCAACCAACTTTGCTATCCTAGTATTTTAGTGTACTAATCAGGATTAGTCTCATTTAATTGGGATATTACACGGTTACATTTTTTTAAGAAAAAACAAGCGTTTGCCCCTGCAGTGTTCACTTTTCAGTTTGTAGAAATGATACTGCTCCTTTTGCTTTAAGATACTAGTATCAAGTGCAACTACGATCAGCCAACCACCACAACGAAGGGATAGGAAACGGCTCGGTGATCTGATCAGGGTGTTGTTGTCTTCATATCTTGGTAGGACAGGCACATCCGAAGCTTCTTCACCTCAAGTTCGCAGCTCTCACCAACACAAATACTATAATATCTCTTCAGAAAATTTATGTACTTTTCATCACTCATCGCAGCTGCTAGTATAAATAATCAGCTCAGCTGGTGCCTGTTCCGGAAGCAGATCATCACCTCTCCACCCGTTTCTTTCTCGGCCCATCCAACACGCGCTCTTGCTTGAACGCGCAAAGAGAAACACGACGGGCGAGGCGAGCGAGATGAGGTTCTTCCTCAAGCTCGCTCCTCGGTGTTCCGTGCTGCTTCTCCTCTTGCTGGTGACGGCGTCGCGGGGGCTTAACATCGGCGACCTGCTTGGCAGCACGCCGGCGGTGAGAACCAGCGTGGAGCTAGCTGACCAAGTCTGATTTTTTGCAGTTACTTGTTATTGATCAGTTGATTGATTTTCGCCTTTGGCTTTGTTTGTTTTCGGCGCAGAAGGACCAGGGATGTAGCCGGACGTGCGAATCCCAGTTTTGCACAAGTACGTAGTACGCCACTCCTCTTACTAATAATCTGACAGGCTGATACTGCCTACAGAGCAGAGAAATCTTTGCAGTTCTGGGGCTTCTCTTCCCCCTGCTTCTGAAATGAAAAAAAAAAAAAACAAGCAGAGCATATGATGCTAGCCAAGGCATTAGATGCAGACTTCTTGTCTTTGAATCCTTCCGTGTCACCGTTAATTTGACTGAACATGATATGCATTCTTGAGTTGGTGTTCCGGCCGTCCGGCAAAGGGAGCTGACACGCATTTTGCCTTTTTTTGCTGGGTGTCCGTGATGCAACCGACGACCTCTGAAAGTTGCACCTCTGCTGAGGTACGGCAAGTACTGCGGGATCCTCTACAGCGGGTGCCCCGGCGAGAGGCC

>VIT_211s0103g00190

TTCCCCTAACTGAGGATAAATGACATTCCTTACACACACATTGATTAAGCATACATTCATCTCATGTACTTTATCTAACTTGTATATGGTCATTTGAATAGGTACCTCACTTATGATGATTGTAAAACAAAATTATACAACAATTTTTCTTCTTTTTTAAAACCAAACCAATGCAAACATGGTTAATCGACCTATTGTCAGATATTCATGAAATGATGTATGAAATTTAAGTTATTGTATTAGAGTATTACACTAACTGTACAAGACAAATTTACTAATTATACAAGGGTGTACAAGACTACTTTTTGTTAAGGATTTCAAGTGATTTTGAAAAACTTTCCCATTTTTTTCCACTCGAAGATTTTTTTCCCCACTCAAATTCTCTCACTCAAGAATTGTTTCGAATTTTATTTTTAAATTTCATTATCAAAATTTTGTAATTGCATATTTTGTTTTTGTAGTTTTAGCAATGTTCTTTCTTTCCACTATTTTTTTTTTTTGTGAAATTTTATCCAAATGAATCTATATTTAAAATTATAATCATATTTATCAAATTTTTTACTAAGATTATCTTTAATTCTTTTAATATATTTATACTCATTTATTTAAAAAATGAAAAACTTATTTATTTTTATTTTTTTGTAAACATGTTCTATTACCTTTCAAGTAAAAAAATAAGATAAGTTTCAAAGTCAATAAAAAAAAAATTAAATACCACTTTCAATAATTTTTAGATAAAATTTTATATAATATATTTTATTTGAAATTTAATTTCTAAAGTTTAATTAAAAAATTGTGTTGACAATTTTCTTGTTGTGAGTCAAAATACTTTGCATTAGTCTATCTAGATAAGAAAAATTATTAATATAATATTAGAACTTCTTATCTTAGTTATTTTTTTCAATAAATTTATCTTTTTAAAAATTTTAAAATAAAAACATTAAAAATTTAAAAGCTAAAAGGATATATATATATATATATATATATATATATATATATATATATAATAAAGTGAAGTGATAGTCAATTTTTAAATTTTTTAAAATATGGTTAATGTAGAAAATATAAAAAATAAGAGAAAATATAAATGAAAGGGTAATATAAATTTAAAATAAAAAATGAAAATTAAACTAAAAGATAAATACAAAAAGATAAAAAATATTTGGATGAAAAATCTCAAAAATTATATCATTGCTTATAATAAGAGCAAAAAGATTCAATATCTTTAAAAATGAAAAACAAAAAAACATTATTGCTTTTTATTTGACATAAAATAAAAATATAGATTGGAAGAAAAAGAAAGAAAAAAAAGTTAGAAATGAGTAATACTTAATAGGAAATAGAAAAAGAAAAAAAAAACACACAAAAATTGAAATTCACACTCATTTGTGTCTATGTAAGGGTTAACAGTATTTTAGGGATTAATGAAGGACAAGATCCTTCATCTTAATTTTTATACTTTGTTTGGGTATTGTGTTTAAATATGCATGATATATAGACCAAATATTTATTTTTGGACCAGTTGGCACAAAACACAATTCTCCCAAATTAAAAGGGAAGAGTGCAGAAACATGTTGCTTTTGCAGGTCACAATAATTGATATGAGTAGCATTGACCAACAAACACCCCATGGTGAGGATAGACTACAGAGGAGGGAAGAATTGGATGAATGATGAGTGAGTTGGTTCAAAGGCCATGGTCACGAGATGGAACCCACAACTACCAATATATTTATAATGAAATTAAGGTGAAAAAAACTGGCTGACAACCAGTGTGAACAACACTCCAAAAAACAAAGGTCACGAGCCATTACAAGACAAACGCCTATTAACCCACCCCTAGGCTTCTATACACCGATCTCTCCCCCTCACACTCCCCCATCCATTCTTTTTCTCTATAAATCAGAGCCCTTTTTCCGAAACCCCAAGCTCCATTTTTCAGTGCCTCTCTTCTCTCCTCTTCTCTTCTCTCAATTCTCTGCTGCTTCTGCAGAATCAACA

>VIT_211s0016g02570

CAAAATGCATAGGGTATACTTCATACAAAAAGACAGAAGAGACATAAAAAAAACATCCCTGCGACTTCACGTGTGGAGGGAGTTGTTGAGAGTCGAGGGCTTTTTCCCGTTTGTTGAATTTATTGGGCCTACGTCTCTAAGCATTTTCAAACCAAAAGTCAACTTGTCCACAACGCTCACAGACGTGGCCCCCCAACACTACCCCCGGAATGGATACGATAACATCCCTTCAAAAATTGTAACATTATGTGGCAAGCTCACACAGATTGCCACATGGGCGGGACTCCATATGTATAGTACTTCCCCAGCTGCACCCTCTTGAACTACTAAATTAAAGCTACTGGACACTGTAATGAATTCAATTGTATAGCTGGCTGCCGTGATCCCCGCTGAACACCATGGATCAGTAATCAGTATAAGGTCCACATTTGAACGTAATCAGCAAGGCTAGTCATCATGATGGTAAGGGGTAAAGACTTGTATTTCTAATTTTAATGGCAAGATAATGTTAAGTAATATGCATATTTCAAGTCCAGGAAAATGTACTTCTTCAAAGCCATCATGGGGCTGCTGGACTATCACAGGCGGGATTGTAAGTTTGTAAACAATCTGGGTATTCGGAGTTACGACGGTATGATCGGGTCTATACGTTTTTTATGGAATGAAAAAGACAAATATGATAGTATCCTAACTATAGAATGTGACCTTAATCAAAATACATACATTTGTCTCTTACAAATTTTTTTATTAATCTATATATAAAGTGTCTTCTACTCGACCCATTTCTCCAAAACAATTCACAATATGTTTGATAGTGATTATGAAAAGTGTTTTTAAGTTTTGTAACACTTAAATTTTTTTATTTTTTAAATATTAGAAATGTTAAAAATATTTTTCAAAATCACTGTCAAACGCATTATATTAAAGCCCATAAAAGTGTTTTTTAAAAAACATTATTAAATAAGATTTTAAAGGATGTTAAAACTTATTTGACAATAATTTTAAGAAGCATTTTTATATTTTCTAATATTTGAAAATAACCCTTTTAATAAAAAAAATTAAGTATTGAAAATATTAGGATCACTGTCAAACGAGTTCTTAAAAACTCGTCATAATACTTGAAAAGATCTCTTTATAAAAAATATAGTAAAAAATCACTTTTAAAAAATTAGAGAATCAATTTCAAGTTATTTTTAGATAACTTCTCAAATAATAACGAACTATCAAAAGCAATTTTTTTTTTTTTAATTCACATCAAAACACTTCCAATAAAAAGTGTGTGACTATTAAAATCACTTTCAAGAGAATTCTTCAACTTCAAAACACTTGAAGATTAAAAAGTGTTTCTAATAAGAAGATTTTATTACATACAATAACTTTCCTTAAGACTTGGTTATTTGACCCAATGGAACAATCCTCCATATATAGGCACCCATGATCAATTTGAAAGAATGAAGATTTTATTACATATTTGTAAGGTTTTAATAGCTTTGAAGGCTCTCAAAGGCTTGGTTGCTTCATCGGAGGAGACATTGTCTATTTATAGGCATCGAAAGAAGGCTCTCAAGGCTTGGTTGCTTCACCAGATGAGACCATCGTCTATTTATAGGCACCAAAAAAGACGGCTAATAGTTTCATGAGATTTCTTTTTACAACATTTTATACAAAATAAAATTATAGAAGGCCCAGGAATTAAAACTGAAAAAATACATTGGCCCATGATGGCAATTCCATCTGGACTCAACTGAACATAAAATGGGCCTAGCTCGGGAGGCCCATGTGAACCTTCTAATTAAAGCACATTCCTCTGGGTCCATTGGGCTGCCCATTTTCATATGGGCCTAGCACTTAGCTTACAGGACGAAGATGTTGGAAAGACATTGAGGTAATTTCCACTTTTCTTTTCTTTTTCTAATTTTTTTTTCCCTTCCTCATTTGCTGCGACGACGAGACAGGAGCTGGACGTCGAAGAGAATTTTTGTCTTCTGAGCCGAGAGAGA

>A. thaliana TAIR10|AT4G29070|Chr4:14321812..14323307

TGATGGACTGCAAGAATGCTCTTTCAGAGAGTGAGGGCGATATGGTCAAAGCTCAAGAGTACCTCCGCAAGAAGGGACTAGCGAGCGCAGATAAGAAAGCAAGCAGAGCCACATCCGAGGGAAGAATTGGTGCTTATATCCACGACAGCAGAATCGGTGTCCTCTTGGAGGTTAATTGTGAGACTGATTTTGTCTCACGTGGCGACATTTTCAAGGAACTTGTTGATGATCTGGCGATGCAGGTAACTCCAAGTTTCTAGCTGAAACATTTTCTTCGTTTTGCTATAACTGAGAATATAATTTGACCTGACATGTAGGTGGCTGCGTGTCCTCAAGTAGAGTATCTTGTAACAGAAGATGTTTCAGAAGAAATTGTGAAGAAAGAAAAAGAGATAGAGATGCAAAAGGAAGATCTTTTGTCGAAACCGGAACAAATAAGGGAGAAGATAGTGGACGGTCGGATAAAGAAAAGGCTAGATTCACTTGCATTGCTTGAGCAACCATACATTAAAGACGATAAGGTGATAGTTAAGGATCTTGTAAAGCAGAGGATTGCAACCATTGGAGAAAACATCAAAGTGAAGAGATTTGTGAGGTACACTCTCGGAGAAGGCCTTGAGAAGAAAAGCCAGGACTTTGCTGCTGAGGTTGCTGCCCAAACTGCAGCTAAACCGAAAGCTAAAGAAGAGCCAAAAGCTGAAGAGGCCAAGGAAGCTGTTGCAAGGTGAGAAATTTTTTATAGTCTTTGCTCTTCTTGACATGAATTCTTATAATGTTGGCTATAGAAAGATTCAAACTTTTTGCAATACGGCACTGCAGCCCACCAACTACAGTGGTTTCAGCTGCTCTTGTAAAGCAACTGCGTGAAGAGACAGGAGCTGGAATGATGGATTGCAAGAAGGCATTGGCGGCGACAGGAGGAGATCTTGAGAAAGCACAAGAATTCCTCAGAAAGAAGGGTCTCTCATCAGCTGATAAAAAATCAAGCCGGCTTGCATCAGAAGGGAGAATCGGTTCGTACATCCACGATTCTCGGATTGGAGTTCTGATAGAAGTGAACTGTGAGACTGACTTTGTCGGAAGAAGCGAAAAATTCAAGGAATTGGTTGATGATCTTGCAATGCAAGCAGTGGCTAATCCACAGGTAAGAAAATGTCCTCAGCTGAAACAATGGCATAAATTGGTTGTTGGTCTCTGACCTGAAATGTCTACGTGCAGGTGCAATATGTTTCAATAGAGGACATTCCAGAGGAGATAAAGCAGAAAGAAAAAGAGATTGAGATGCAAAGAGAGGATCTTTTGTCGAAACCAGAGAACATAAGGGAGAAGATTGTGGAAGGAAGAATCTCAAAGAGGCTTGGAGAATGGGCATTGCTTGAGCAGCCTTACATCAAAGACGACAGTGTTCTGGTGAAGGATCTGGTGAAGCAAACAGTGGCTACTCTTGGGGAAAACATCAAAGTCAGAAGGTTTGTGAAGTTTACTCTTGGAGAAGATAACTGATGAGACAGACGAAGTAAAGTTTTCAAATAAGGGAGCTCTGTTTTGTGTCATGGAGTTTAAAGTAAAAGTCTTCCTTTTTGTTTTGTAGACAAAATCTTCAGTATTCACATTTTGATTAGTTTTTAATCAAAACCTGTAATTTTTTGTTCATGAAAATATGAGATTTGCATTATATAAATGTGCATTTAGTGACAATGTGACATTATACACTAAATTCGGAGAAAGATTGGTTATAATGAAATTTTAAACCTTATTTTAAATAGAAGTTTCAAATGAATTTCCCTGAAGAAGTGAAACTAAATAAAATAATTTTTTGTTTCCTTTTGGATATTATATCTAAATATAAAAATCAAACCGACCGAATTACTACATACACTACAATAATGTTTTTTTCCTTTTATAATTTGTGGGCTAAAAAAGGTCGGTCGGCCCATTTTGAGCCCAATAAAATTTAGGGATAGATAAAGACATAAAGTAACGAACGATATTCCGAGGTTGTCATTTGCTAAGGAAAAAAAAAACGAAAACGTGTGTCTGTCTCTTCTCGTAGCGTCTCTCAAGCTCAGGTTCGTGAACATTCCAATCTCTCAATGCAAAAGCTCATTCGAATTATCCTTCTCTGTTATTTTCGAGATCCGATTTACTCTTCTTTACTAGTCAATTCTAGGGTTTTGAGTATTGATCTTTCGCAATTTCGAAATTTTCCCAATTTGAAAAGATAAAAATCTTCCCTTTTTGTCTGGATCACACTTGATTGCTGAAGTCATTGCATGCACTGTTTTGTTGTTTACAG

>O.sativa v7.0|LOC_Os07g46420|Chr7:27699931..27701407

TAGGGGACAGGTACCTAACACATTTGCTTGTACTGTGGCTTCATATTGGTGCAAACATTTATGTGAAAACTGATTTGAGCTTGCTTCCTTTGCAGTATGCACTCCGCAAAGTATATCATGAAAGTCCAAGGCTTGTCTGTGTTCTATATACTTCTCCAACATGTGGTCCTTGCAGAACCTTAAAGCCAATTCTAAGCAAGGTTTGTTCTTGAAATTGAAATTGGGCTCTAGTCGTCACAAGTCATCACTCTAGACAAATAAAATGCCTTGATATACAGCTATCTGATTGGCCCTCCAGTCCATCGATCATTATGCATTCATATTGACAGGCCATGTTAACGTTCATTTCTCTGATCTACCAATATGACCAATCAAGTTCATTTCTCTGATCTACAATATCGAATCATTAAAGAAAATCCATTTCAAGGTGCTAACTTTGTGATGTTCTTGTGGTCTGGACAACACAGGTTTTCAGTGCTTCTTGATAAACCTTGTAGAACTGATATTTATAGTTGCTTGACTAACATTTGATTGCCGCACCATTCTACAGAAGTTCCGTTCTGTTAGTCATGCTTGTGTTAGTTTTGTTGTTAACGTTGCTAGTTGCTACTTGGCACTTGCTAATATTGATATCTTTTTCAGGTTATAGATGAGTACAACGAACACGTTCATTTTGTCGAAATTGACATCGAGGAGGATCCTGAAATAGCAGAAGCTGCAGGCATCATGGGAACACCATGTGTTCAATTTTTTAAAAATAAAGAAATGCTCAGGTTGGCTATCTTGGATTTGTTTATCTGCTACTAGTCTATATAAACATCAGTCACTTCATTGCTGCCTAATTAATATGATGCAATGGAATCCCTTCTGATCGTTTTTAACCTTGGACTATGCAGGACTGTCTCTGGTGTTAAAATGAAGAAGGAATATAGGGAGTTTATCGAGTCAAACAAATGAGCTGAACCCAACATACGATTCAAATTTTAAAGTGTAATAGTCTTCTTTTGCCCATACTGACTATTTCTAGCTGCCACCGTGGTGGCCAATTTTTGGTCAAAAATTTTCAGATGGGCCGGGTGTAGTGCGGTTCAAGAGGAAATTAGCCGGTCGCTCCTATTGAATTCAATCAATGTAACAGAATATATTCTTTTGCTGTACAACAACGTATTGGTCGTTGTCTTGACACCGTGCAAACTCCTCCCTGACAATATATACGTCTTAAAGATTTTGGACAGTGTGTGATCTATATGCTGAACATAATATTCATATGCTTATAGTGAGCTTGCTGGAAAGATACCCTGACATAAAACTATTAAGGCATTGTTTTTGGAACAGGGAAATTTTACATTAAATTTATAGAAAATGGATAGGTTCATGTGAAAATCCAATGAAATTAATATGTTTGAAACGAGCCTTTTCTTTGCGTAGCAGAGCAGAGGATGGTTGTTTGGGAAAGCTTCTGTGGCATTGCAGTTTCTTTCAAAATCTATAACTCTTCTTAAACAGTCACTGTTCACTATATTCTCAGAATCAGAATCTACTACAGAATCTAAAAATGACCAGCTCAAAAAACTATAATTTGAAGATAAAGCTAAAGACTCCAGAAAATAAGTCCGCAGGGCCGGCCGAACACTCAGGAGAACACCACGTTTTTCCAGTCTCATCTCATCCTTACGCGCCGACCGGGCGGCCGACTCCGCCGCCGCCGCCGCTCCAAACCGGCAACCAAAAACGAGCGGCGCACGGCAAAAGCAATCACAAATTCACAATTCACAGCTCACCTCGGCGAGGCGCCAAGCCAAGACGAGACGACGAGTCACGCCACCACGCGTCACCCTTCCGCCGCGCCGGTGCCTCCTCCAGAATCCTCCTCCGTTTTATCCCCACCCAAAACCGGCGAACCACCCGCCCGCCCAATCCCCGCCTCAAATAACCCCCCCTCGCCGTGGCCCCCACCCCCAACAAACTACCAAAAACGAAAAAAAACGTAACAAATCCGCACAGCCACACGCCACGCCACGCCACGGCCACCGCCGCCGCGCGACGCGACGCGAACCCGGAGAAAACGGGGGCGAAGGAATCCGCCACCGCA

>V.vinifera v2.1|VIT_206s0009g03260|chr6:16485011..16485896

AAGGTGGATTAAAGATATTTTTGTATTATGTTTGATATTCTTTTATGGGCTTTTTAAAGAATGTGCTAAACTTAGACTTGATTTTAATCGAGGGTGATAAACACATGTTTTTGGAATAAAGTTTAATCGATTGTCACTTAATATCAAGTAAACTTATTAATAATTTCCTCAAGTGATTTGAAAAAACCTGATTTTCTCTTTTTTTGGCTTTAAAAGTGGATGAATGACACTTTTATATCATATTTAATATTATTTCACAAGCTTATTAAAAAATGTTTAGAGTTTGATTTTTGAATTTAACTTTTGGGTTGCATTTTCCTTTTGATAACATCTCATACCCACTAATCTATATAGAGAGGGCTAGAGTGCTTTTTAAAAGCAATGAACTCGTCGAAGAGATTACGAGAGTTTTGCCTTGTATTTTTTTAGAAATATTTGGTTAAAATGGATGAAATAATCTTCAAATCGGAAATCTAACCATTCCCATGTTTTTTTTGGAAAAGGAATCAATTTTTGACATAAATACCCTTTTCTATTTTAAGTATTTATATGTAGGTTTTAAAAGAAATGGTTATTTTTATAAGAAAATGATGAGGGTATTTTTGTCCAAATGATGCTAAAAAAGGGTGAGAGGTTAAATTTCAAAAATCGGGTTTTGAGAGGCCTTTTTTATAGTGAGTAAATATACAAGTCGGAGGATTGAAGCCTAAAAACTTTGCGTATTTTGTGGGTAACTTTAAAGAAACATAGGTTGGCATGCCTAACCTATTATTATCTTATCCAACAATAATGCTGTCATAAAACCCCATCCCAAACACACGCCCAAAATTCTCTCATAAAAAATAAAACCAATAAAAAAATGACACGCAGGCACACGGCGGACCAAGGAATCAAAGAGGATTATATACTTGCATAAAAACAAAATGAGTCTATTTCTATGTAAGGAGTGGCGGCCCTTTCTTCTAATCCTGGCTTCTCAGTCCACAAGCCAACCTCCTCTCCTCTCCATAAGCTACCTCTCTCTCTCTCTCTCTCTCTCTCTCTCTCTCTCTCTCTGTGCTCAAGATCACATCCCTCAGGTCAGTCCCTTCTCATCTTTGGGTTTTCTTCTTTTCTGCTTATTTGTCTTCTTGGTCCTCCTCTGTTTGGTTGCCTAGACTCCTCTAGAAAAAAAGAAGAGAGGAAGAACAATCGAAGAAAGACAATTTGGTGTCATGTTTTTGCTTGCGCAAGTGGTTGCTTCATAAACTGTCAATTGATTTTGCCTTTAATCACAAAATGGAGACCAATAACACAACAAAAAAAAAAAAACTCCTTTGATTGTTTGCAGTTTAAATATAATGTTTTGGAACGATGGTAAGGTTTGAGTATCATAAGAAGGAAATTCTGGATGCGGATGAATATTTTTAGTGTTTTCTCCGTTTTAAGAGTACAAAGCAAGTTGTTGGATTTTCAAACATGTCCCTGTTTCCAGAAAAAAAAAAGCTGCAGAGAAATTGAAATATGTGGGTTTTCTGTGTGGTTTCTTTTTTAGGATTGTAAATACACCTCTCGATGAAGCCAAGTAGTTGTTGTAATAGACCTGGTGAAGTCAATCTTTTATTTTCTTGGTTTTAAATAAGAAGAATATTTAGGATTTTTAAAAATTTGTTTTAATTAATTTTCCTACATTTTGATTGCAATCATATGGATTTTATTTTCTCTTTTTGTGATTCAAATGGTGAAATCTTCAGTTCACTGTGAATATTATACTTATTGTAGTAACACAAACAATGATTTAGGTGTTGATCCAATTTAATAGGGTTGTATATACTGCTTCTGGGTTTGATATGATTATAGGTTTTGAGACCTGTGATAAATATTTATGGTTCTGCTCACCTTTTCCCAAGGTTGTGTGCTTTTCTTGACAGAGCAATGTGGTTTTCATGACAGGTTCATTAGCTTGATGATGCTGTGGTGATCAAACTGTGTGATTTAATGAAATATTGTGTTCATTAGTG

>A.trichopoda v1.0|scaffold00063.74|AmTr_v1.0_scaffold00063:3500829

GTTCATATCTAAATGATTATAACAAAGAATCAAATGGTGACCGTTTAACTCAGAAATCCAAGTGACTTCGAGGACATACTCTTATAAGAGTAATTCAAGATCATGTTGAGTGGCTTAGAAGGATTTGAAAATCCAATAGTTTTCCATGCATCACGGTCTGAAGGATACCGTACTGACCCCCTTAAGCATCACGACACTTTAAATACCATGATGCTCAAGGCTACTAGAAATTTAGGCATCACGGTATTTACAGTGTCGTGATGCTTGGGGAGTCATACCTTTTAGATAGTGATACTTGAAAAATCCAATAGCTTTTCACTCATCACGGTCATTAAAGTTCCATTATGAGGCTTTTTAACATCATGAGGCTTTTAAACATCTTGATGCTTAAATTTCCAGTAACTCTTGAGCATCACGGTGTTTAAAGTGTCGTAATACTTAGGGGACTCATCACGGTATCTTTTAGACCATGGTGCTTGAAAATCTACTAAATTTCTAGTAGCATGGTCCGAAGAGCACCATGACTACTTCCCTAAACATCACGGCATTGTAAACGTCGTGATGCTCAAAGTTACTGAAATTTTACCGTGATGCTTGAAATCTTCAGAATTTAAAATCTTTGTCAGATGGTAACATCATTCATAGAGATGAACCATAGCATGGCTAGATGGTAAAAAGGCAATCCTCATTCATAGAGATGAACCATAGCATGTCTAGAAGGTAAAAAGGCAATCCAAGCTATCGACTTAGCAATGTTAAACGCAATGCACCATGCGCCTACCTGAGAACCCAATACCAATAAAGAGAGAGCAAGAGAGAGGTAGAGAGAGAGTACCAACTACAAAATATAAATTAGAGATCACAAAACTATATATAAAGCACCTAATATCGAAATAACATAATTGTGTACAACATGCCGCAAAAACGTACATTAACAGGCACTACTTACCTCTTTACGTCATGAAATATAATAATGAATATTTACAGATGCCACAATATAAATACAAATCTAGAATTTATTTATGTAATATTTTTAAATGAAAAAAATTCCAATAACCAACCAACTGCATCAAGTACTATGGAAAAAACTACACAAAAATTCATACAAAGTATTACCATCGCACATCCAAGGGATTAAAGGTTTGAAGATTTTGAAGACATACCTATAGAATGGTGTTGGTTTTAGTGTATATATTTTATGCTCATAGATTGGGAAATAGGTTTCAATGACCTTGCTTAGAGATTGAGAAATGGAAGGTGAGGATATCAAATAGATGAGAGAGAAAGAAGGCTAAGGAAAAGAGAGGTGGGGTTAGTGACAGCTGGGAATATAGGGTTTTGAGAAGAAGAAAATTGAGGGAAGGAAGGAAAAAAGTACAAGAATGTTAGTTTAGAAGTAATAACATATGTTCTATAACCACTATTACTACTAATATGATAACGACATTTTAAATATCATGATACCTCTTATGGTATTTACGGTATTTTAAATAGTGTGATGGCTTAAAACATTAAGTCCATCACGACATTGATTGTACTGTGATGCCTTCATAGTTGTATGACTACTGTGAATGCTATTTTAGTCAAAATAACATGAGAAGTGAAAAAAAATAATAAAGACGAGGTTTAAAAAAAGTATCTAGTTTTTAATGAACTAAAAATTTAACTATATTTTTGATAATTTTTCAAATATCTACTATAAAAGTAGTTGTAAGGTGCCTAAGTGAAATCCAAACTCAAGGTAACCGGTTCACCAATATACTATTTCTGTAAACCGCCTTTATCGGATAAATGAGAGAATGGGTACCTAGTACCAAACAAAATTCCCGCGAGAACGTATTAAATGAGCTGAAAATCCCAGTTACAAGTCTGTGGACTTTGCGGAGCCCACCACTACACTCTATCCTCGGGGCCACCTCCCCTCCCACTTTCTCTCTCTACGTTTCCTCTTCTCTCTCTCTCTCTGCGCCGATTCCTGTCCATCAAATAATTGATGATCC
